# Effect of a Personalized Diet to Reduce Postprandial Glycemic Response vs a Low-fat Diet on Weight Loss in Adults With Abnormal Glucose Metabolism and Obesity: A Randomized Clinical Trial

**DOI:** 10.1001/jamanetworkopen.2022.33760

**Published:** 2022-09-28

**Authors:** Collin J. Popp, Lu Hu, Anna Y. Kharmats, Margaret Curran, Lauren Berube, Chan Wang, Mary Lou Pompeii, Paige Illiano, David E. St-Jules, Meredith Mottern, Huilin Li, Natasha Williams, Antoinette Schoenthaler, Eran Segal, Anastasia Godneva, Diana Thomas, Michael Bergman, Ann Marie Schmidt, Mary Ann Sevick

**Affiliations:** 1 Institute for Excellence in Health Equity, Center for Healthful Behavior Change, Department of Population Health, NYU Langone Health, New York, New York; 2 Division of Biostatistics, Department of Population Health, NYU Langone Health, New York, New York; 3 Department of Nutrition, University of Nevada, Reno; 4 Department of Computer Science and Applied Mathematics, Weizmann Institute of Science, Rehovot, Israel; 5 Department of Mathematical Sciences, United States Military Academy, West Point, New York; 6 Division of Endocrinology, Diabetes and Metabolism, Department of Medicine, NYU Langone Health, New York, New York; 7 Diabetes Research Program, Department of Medicine, NYU Langone Health, New York, New York

## Abstract

**Importance:**

Interindividual variability in postprandial glycemic response (PPGR) to the same foods may explain why low glycemic index or load and low-carbohydrate diet interventions have mixed weight loss outcomes. A precision nutrition approach that estimates personalized PPGR to specific foods may be more efficacious for weight loss.

**Objective:**

To compare a standardized low-fat vs a personalized diet regarding percentage of weight loss in adults with abnormal glucose metabolism and obesity.

**Design, Setting, and Participants:**

The Personal Diet Study was a single-center, population-based, 6-month randomized clinical trial with measurements at baseline (0 months) and 3 and 6 months conducted from February 12, 2018, to October 28, 2021. A total of 269 adults aged 18 to 80 years with a body mass index (calculated as weight in kilograms divided by height in meters squared) ranging from 27 to 50 and a hemoglobin A_1c_ level ranging from 5.7% to 8.0% were recruited. Individuals were excluded if receiving medications other than metformin or with evidence of kidney disease, assessed as an estimated glomerular filtration rate of less than 60 mL/min/1.73 m^2^ using the Chronic Kidney Disease Epidemiology Collaboration equation, to avoid recruiting patients with advanced type 2 diabetes.

**Interventions:**

Participants were randomized to either a low-fat diet (<25% of energy intake; standardized group) or a personalized diet that estimates PPGR to foods using a machine learning algorithm (personalized group). Participants in both groups received a total of 14 behavioral counseling sessions and self-monitored dietary intake. In addition, the participants in the personalized group received color-coded meal scores on estimated PPGR delivered via a mobile app.

**Main Outcomes and Measures:**

The primary outcome was the percentage of weight loss from baseline to 6 months. Secondary outcomes included changes in body composition (fat mass, fat-free mass, and percentage of body weight), resting energy expenditure, and adaptive thermogenesis. Data were collected at baseline and 3 and 6 months. Analysis was based on intention to treat using linear mixed modeling.

**Results:**

Of a total of 204 adults randomized, 199 (102 in the personalized group vs 97 in the standardized group) contributed data (mean [SD] age, 58 [11] years; 133 women [66.8%]; mean [SD] body mass index, 33.9 [4.8]). Weight change at 6 months was −4.31% (95% CI, −5.37% to −3.24%) for the standardized group and −3.26% (95% CI, −4.25% to −2.26%) for the personalized group, which was not significantly different (difference between groups, 1.05% [95% CI, −0.40% to 2.50%]; *P* = .16). There were no between-group differences in body composition and adaptive thermogenesis; however, the change in resting energy expenditure was significantly greater in the standardized group from 0 to 6 months (difference between groups, 92.3 [95% CI, 0.9-183.8] kcal/d; *P* = .05).

**Conclusions and Relevance:**

A personalized diet targeting a reduction in PPGR did not result in greater weight loss compared with a low-fat diet at 6 months. Future studies should assess methods of increasing dietary self-monitoring adherence and intervention exposure.

**Trial Registration:**

ClinicalTrials.gov Identifier: NCT03336411

## Key Points

**Question:**

What is the effect of a precision nutrition intervention aimed to reduce the postprandial glycemic response to foods on weight loss in adults with abnormal glucose metabolism and obesity compared with a low-fat diet?

**Findings:**

In this randomized clinical trial that included 204 adults who received web-based group behavioral counseling, there was no significant difference in percentage of weight loss at 6 months between participants who followed a personalized diet compared with those who followed a standardized low-fat diet.

**Meaning:**

This study found that a precision nutrition intervention targeting a reduction in postprandial glycemic response did not result in greater weight loss compared with a low-fat diet.

## Teaser

This randomized clinical trial compares personalized vs standardized caloric-restricted weight loss interventions in terms of percentage of weight loss among adults with high postprandial glycemic responses and obesity.

## Introduction

There is considerable debate regarding the best diet for weight loss. A commonly held view suggests that obesity develops from repeatedly high postprandial glycemic response (PPGR) that produces a downstream biological cascade of events resulting in weight gain.^1^ Because carbohydrates primarily influence PPGR, diets aimed to minimize PPGR (eg, low-carbohydrate diets) have been hypothesized to induce weight loss in behavioral interventions. However, when compared with low-fat diets, the effects of low carbohydrate and low glycemic index or low glycemic load diets on weight loss are mixed.^2,3,4,5,6,7,8^

Growing evidence shows that interindividual variability in PPGR after the same meals may be attributed to an individual’s physiological characteristics and lifestyle behaviors.^9,10^ Specifically, the gut microbiome has been shown to contribute extensively to an individual’s PPGR.^10,11,12^ Collectively, factors such as dietary adherence and the gut microbiome are not considered in one-size-fits-all dietary recommendations (eg, low-fat diets); therefore, a personalized approach may be more advantageous for weight loss.

In 2015, Zeevi et al^10^ developed a supervised learning model termed *gradient boosting regression* on a training data set (n = 800) using input data from clinical and microbiome measurements to estimate the incremental area under the curve from postprandial glucose curves.^13^ The model was evaluated on the training data set using leave-out-one cross-validation and also on an independent test data set (n = 100) yielding similar Pearson correlation coefficients that used features of the gut microbiome.^10^ In a 6-month randomized clinical trial in people with prediabetes,^14^ the algorithm-tailored diet reduced PPGR more successfully than among individuals who were prescribed a Mediterranean diet. Despite these promising results, to the best of our knowledge, no studies have used this precision nutrition approach to promote weight loss in adults with prediabetes and moderately controlled type 2 diabetes.

The primary objective of the Personal Diet Study was to compare 2 caloric-restricted weight loss interventions in adults with abnormal glucose metabolism and obesity in terms of percentage of weight loss at 6 months: (1) a one-size-fits-all low-fat (hereafter termed *standardized*) diet or (2) a personalized diet developed by the machine learning algorithm to estimate and minimize PPGR to meals (hereafter termed *personalized*).^14^ We hypothesized that the personalized group would experience a significantly greater percentage of weight loss compared with the standardized group at 6 months. We also examined between-arm differences in body composition, resting energy expenditure (REE), and adaptive thermogenesis (AT) at 3 and 6 months.

## Methods

**Figure 1.**
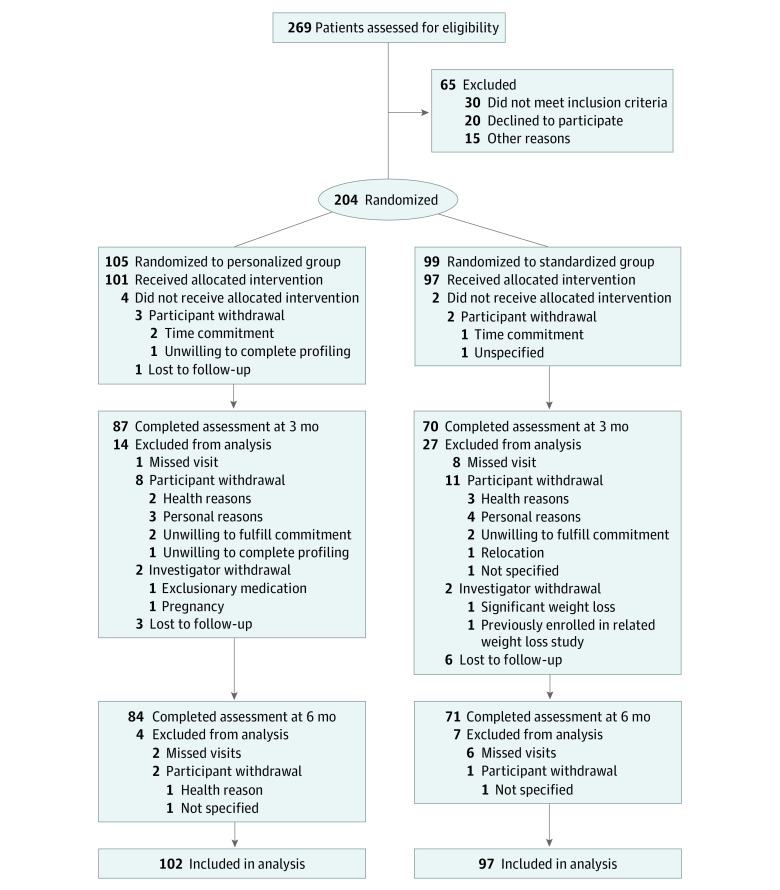
Study Flowchart

The study was approved by the institutional review board of NYU Grossman School of Medicine. All participants provided written informed consent before the baseline assessment. The study protocol is included in Supplement 1. This study followed the Consolidated Standards of Reporting Trials (CONSORT) reporting guideline for randomized clinical trials (Figure 1).

### Research Design

The Personal Diet Study was a 2-phase, parallel-group clinical trial consisting of 6 months of active intervention followed by 6 months of maintenance and observation. Participants were recruited primarily from NYU Langone Health and its affiliates between February 12, 2018, and October 28, 2021 (eFigure 1 in Supplement 2). Participants were randomized, with equal allocation, to the personalized or standardized groups. Randomization was performed by the statistician (H.L.) not involved in intervention delivery or data collection in block sizes of 4, and cohorts of 10 to 20 participants were assembled. Race and ethnicity data were collected because they are potential determinants of weight loss success. The collection of blood and stool samples was required for the machine learning algorithm and generation of personalized PPGR in the personalized group. Because of limited resources, stool samples were only collected in the personalized group, which requires the study coordinator (M.C.) to randomize participants before their baseline visits to prepare for the collection kits. However, owing to required shipment to Israel for processing, reprogramming of the Personalized Nutrition Project (PNP) smartphone application (app), and development of the estimated scores, participants were blinded to their randomization group until week 5 of the intervention. Measurements occurred at baseline and 3 and 6 months. Given COVID-19–related recruitment delays, 12-month measurements were not performed in all participants, and the data were not included owing to the small sample. Modifications to the study because of COVID-19 are presented in the eMethods in Supplement 2 (section 1.2).

### Recruitment

Eligible participants were aged 18 to 80 years, had a body mass index (calculated as weight in kilograms divided by height in meters squared) of 27 to 50, and had prediabetes or moderately controlled type 2 diabetes (defined as a hemoglobin A_1c_ level ≤8.0% while managed with lifestyle modification alone or lifestyle modification plus metformin) but were otherwise healthy. Participants were excluded if they were receiving medications other than metformin or had evidence of kidney disease (assessed as an estimated glomerular filtration rate of less than 60 mL/min/1.73 m^2^ using the Chronic Kidney Disease Epidemiology Collaboration equation) to avoid recruiting patients with advanced type 2 diabetes. Additional details regarding exclusion criteria have been reported elsewhere.^15^ A detailed description of recruitment procedures is presented in the eMethods in Supplement 2 (section 1.1).

### Intervention

Participants in both groups were encouraged to lose 7% of their baseline weight. To achieve this objective, participants in both groups received social cognitive theory–based behavioral counseling and educational content based on the Diabetes Prevention Program. A total of 14 group counseling sessions were delivered by 2 registered dietitian nutritionists (M.L.P. and P.I.) via WebEx. The sessions occurred weekly for the first month and then biweekly for the next 5 months.

Participants were instructed to self-monitor their diet, physical activity, and body weight using the PNP smartphone app. The PNP app was preprogrammed with the weight loss goal (7% of body weight loss), caloric goal (deficit of 500 kcal/d), and physical activity goal (30 min/d). Participants received real-time feedback from the PNP app on calorie intake and macronutrient distribution and feedback reports (eMethods in Supplement 2 [section 1.3]).

### Study Groups

Participants in the standardized group were directed to follow a low-fat diet and were advised to review the PNP app to make sure they were within their targets for caloric intake and intake of total fat (<25% of total energy) and saturated fat (<7% of total energy). A personalized algorithm was used to generate the estimated PPGR from participants randomized to the personalized group using the same model developed by Zeevi et al.^10^ Features used to generate the estimated PPGR included anthropometrics, blood tests (eg, hemoglobin A_1c_ level), lifestyle features derived from questionnaires, and microbiome abundances (species estimated by MetaPhlAn2 [Metagenomic Phylogenetic Analysis]).^10,16^ In addition to feedback on energy and macronutrient composition of meals and snacks, participants received feedback on PPGR in the form of meal scores that were color coded using a traffic light motif. Green indicated foods or meals with good or excellent PPGR; yellow, medium PPGR; and red, bad or very bad PPGR. Participants were instructed and counseled by a registered dietitian nutritionist (M.L.P. or P.I.) to make different food choices or substitutions to change a yellow or a red score to a green score. Details regarding the development of the estimated PPGR and feedback provided to participants have been reported previously.^15^

### Outcome Measurements

All measurements were performed in a fasting state at the NYU Langone Clinical and Translational Science Institute in Bellevue Hospital located in Manhattan before the COVID-19 pandemic. After the pandemic, we adapted our protocol for remote data collection.

#### Percentage of Weight Loss

The primary outcome was the percentage of weight loss at 6 months. Percentage of weight loss was calculated as the difference between baseline and 6-month body weight, divided by baseline body weight and multiplied by 100. Before the COVID-19 pandemic, body weight was measured at the Clinical and Translational Science Institute to the nearest 0.1 kg using a compact foldable scale (Stow-A-Weigh Scale-Tronix; Welch Allyn). After the pandemic, body weight was collected remotely using a Bluetooth-enabled scale (Renpho) (eMethods in Supplement 2 [section 1.2]).

#### Body Composition, Resting Energy Expenditure, and Metabolic Adaptation

Before the COVID-19 pandemic, the secondary outcomes included changes in body composition and AT. Body composition, which includes fat mass, percentage of body fat, and fat-free mass, was assessed using bioelectrical impedance analysis (InBody 270 body composition analyzer; InBody, Inc). Resting energy expenditure was measured using open-circuit indirect calorimetry using a metabolic cart with a flow-dilution canopy hood (Quark RMR; COSMED). Details regarding these study procedures were previously published.^15^ Adaptive thermogenesis was calculated from the difference between measured REE and estimated REE. Estimated REE at 3 and 6 months was determined using a linear regression model, including the independent variables of age, sex, fat mass, and fat-free mass at 3 and 6 months.^17^ The estimated REE equation is detailed in the eMethods in Supplement 2 (section 1.4). After the pandemic, these outcome measures were dropped from the study; thus, these data were available on only a subset of participants.

#### Dietary Intake, Physical Activity, and Adherence to Counseling Sessions and Self-monitoring

Dietary intake was collected using a single dietary recall at each point in a subset of participants using an automated, self-administered, 24-hour dietary assessment tool, versions 2018 and 2020 (eMethods in Supplement 2 [section 1.5]).^18^ Physical activity was measured using a digital device (Fitbit Alta HR; Fitbit, Inc) at baseline only and was reported as steps per day. Counseling session adherence was measured as the percentage of sessions participants attended. Adherence to daily dietary self-monitoring was assessed 2 ways: (1) percentage of days on which more than 0 calories were logged and (2) percentage of days on which at least 50% of their target caloric intake was logged.

### Statistical Analysis

Analysis was based on intention to treat. The project was powered to test the hypothesis that, at 6 months, weight losses in the personalized group would be greater than weight losses in the standardized group.^19^ The sample size calculations were based on the assumption that a clinically meaningful, minimum 5% weight loss would be seen in the personalized group. With a sample of 164 participants (82 per group), type I error α = 0.05, and a power of 80%, we could detect a between-group difference in weight loss as small as 2%. To account for an expected loss of about 20% of participants to dropout, we decided to recruit 200 participants. Changes in the outcomes over time in the personalized and standardized groups were modeled and compared using piecewise linear mixed models, in which 2 periods (0-3 and 4-6 months), group, and group × period interactions were modeled as the fixed effects. When the difference in changing rates between 2 periods was not significant, linear mixed models were used instead, in which period (0-6 months), group, and group × period interactions were modeled as the fixed effects. In all models, participant identification was treated as a random effect to model the within-participant correlations. Age, sex, race and ethnicity, and metformin use were included for adjustment in all models. Race was self-reported with participants selecting from one of the following: (1) African American, (2) Alaska Native/Asian, (3) American Indian, (4) Asian, (5) Native Hawaiian or other Pacific Islander, (6) White, (7) unknown, and (8) other. Ethnicity was self-reported as Hispanic or non-Hispanic. Sex was self-reported, with participants indicating male or female. Subgroup analyses included comparisons of (1) weight loss within participants enrolled before and after the COVID-19 pandemic (March 2020) and (2) those who completed the study and those who dropped out. For subgroup analyses, unpaired 2-tailed *t* tests were performed for continuous variables and χ^2^ tests for categorical variables. Statistical analyses were performed using SAS, version 9.4 (SAS Institute Inc) at a significance level of α = .05 in a 2-tailed test.

## Results

A total of 204 participants were randomized and 199 received the intervention (Table 1). The flow of participants through the trial is presented in Figure 1. The mean (SD) age of participants was 58 (11) years; 133 (66.8%) were women and 66 (33.2%) were men. In terms of race and ethnicity, 49 (24.6%) were African American; 36 (18.1%) were Hispanic; and 108 (54.3%) were White. The mean (SD) body mass index was 33.9 (4.8); the mean (SD) hemoglobin A_1c_ level was 5.8% (0.6%). Overall, 155 of 204 randomized participants (76.0%) completed the 6-month assessment. Those who completed the study were older (mean [SD] age, 59 [10] vs 54 [12] years) and had a lower body weight (mean [SD], 92.8 [16.4] vs 99.5 [18.2] kg) and lower fat mass (mean [SD], 37.4 [9.3] vs 42.7 [12.0] kg) at baseline compared with those who withdrew from the study (eTable 1 in the Supplement 2). Retention rates at 6 months were not significantly different between the 2 groups (71 of 97 [73.2%] in the standardized group vs 84 of 101 [83.2%] in the personalized group; *P* = .21).

**Table 1.** Baseline Characteristics

Characteristic	Participant group		
	All (n = 199)	Standardized diet (n = 97)	Personalized diet (n = 102)
**Demographic**			
Sex, No. (%)			
Female	133 (66.8)	58 (59.8)	75 (73.5)
Male	66 (33.2)	39 (40.2)	27 (26.5)
Age, mean (SD), y	58 (11)	59 (11)	58 (11)
Race, No. (%)			
African American	49 (24.6)	25 (25.8)	24 (23.5)
White	108 (54.3)	55 (56.7)	53 (52.0)
Other^a^	40 (20.1)	17 (17.5)	23 (22.5)
Missing	2 (1.0)	0	2 (2.0)
Ethnicity, No. (%)			
Hispanic	36 (18.1)	17 (17.5)	19 (18.6)
Non-Hispanic	163 (81.9)	80 (82.5)	83 (81.4)
Highest level of education achieved, No. (%)			
High school	29 (14.6)	14 (14.4)	15 (14.7)
Associate degree	14 (7.0)	8 (8.2)	6 (5.9)
Technical degree or certificate	13 (6.5)	6 (6.2)	7 (6.9)
Bachelor’s degree	50 (25.1)	25 (25.8)	25 (24.5)
Master’s degree	63 (31.7)	28 (28.9)	35 (34.3)
Doctoral or professional degree	20 (10.10)	13 (13.4)	7 (6.9)
Missing	10 (5.0)	3 (3.1)	7 (6.9)
Income per year, No. (%), $			
<10 000	1 (0.5)	1 (1.0)	0
10 000-19 999	4 (2.0)	2 (2.1)	2 (2.0)
20 000-29 999	4 (2.0)	2 (2.1)	2 (2.0)
30 000-39 999	10 (5.0)	5 (5.1)	5 (4.9)
40 000-49 999	9 (4.5)	5 (5.1)	4 (3.9)
50 000-74 999	39 (19.6)	20 (20.6)	19 (18.6)
75 000-99 999	30 (15.1)	14 (14.4)	16 (15.7)
≥100 000	76 (38.2)	37 (38.1)	39 (38.2)
Missing	26 (13.1)	11 (11.3)	15 (14.7)
**Anthropometric**			
Body weight, mean (SD), kg			
Both sexes	94.3 (17.0)	93.1(16.0)	95.4 (18.0)
Men	106.0 (16.5)	103.0 (14.7)	110.0 (18.2)
Women	88.7 (14.3)	86.5 (13.3)	90.0 (14.7)
BMI, mean (SD)			
Both sexes^b^	33.9 (4.8)	33.2 (4.46)	34.6 (4.92)
Men	34.0 (4.8)	33.5 (4.6)	34.8 (5.1)
Women	34.0 (4.9)	33.1 (4.7)	34.6 (4.9)
Waist circumference, mean (SD), cm			
Both sexes	108.0 (11.6)	108.0 (11.6)	109.0 (11.6)
Men	116.0 (11.0)	114.0 (9.8)	118.0 (11.8)
Women	105.0 (10.2)	104.0 (10.6)	106.0 (9.9)
Hip circumference, mean (SD), cm^c^			
Both sexes	117.0 (10.3)	115.0 (10.5)	118.0 (9.9)
Men	115.0 (9.9)	114.0 (9.8)	117.0 (9.8)
Women	118.0 (10.4)	116.0 (10.9)	118.0 (9.9)
Body fat, mean (SD), %^d^			
Both sexes	40.8 (7.9)	40.0 (8.12)	41.6 (7.7)
Men	33.3 (6.3)	33.6 (6.1)	32.9 (6.7)
Women	44.5 (5.7)	44.3 (6.2)	44.6 (5.3)
Fat mass, mean (SD), kg^d^			
Both sexes	38.5 (10.3)	37.4 (10.3)	39.5 (10.1)
Men	35.2 (10.3)	34.4 (9.2)	36.3 (11.8)
Women	40.3 (10.0)	39.4 (10.7)	40.7 (9.3)
Fat-free mass, mean (SD), kg^d^			
Both sexes	55.8 (12.1)	56.0 (11.8)	55.7 (12.4)
Men	69.0 (8.6)	67.1 (9.1)	71.7 (7.2)
Women	49.4 (7.5)	48.4 (6.0)	50.1 (8.4)
**Metabolic**			
REE, mean (SD), kcal/d^e^			
Both sexes	1770.0 (390)	1800.0 (406)	1750.0 (377)
Men	2170.0 (338)	2130.0 (356)	2220.0 (312)
Women	1580.0 (237)	1570.0 (245)	1590.0 (234)
Respiratory quotient^e^			
Both sexes	0.83 (0.11)	0.83 (0.11)	0.84 (0.11)
Men	0.84 (0.12)	0.84 (0.13)	0.85 (0.09)
Women	0.83 (0.11)	0.82 (0.09)	0.83 (0.12)
Hemoglobin A_1c_ level, mean (SD), %	5.8 (0.6)	5.8 (0.6)	5.8 (0.5)
Metformin use, No. (%)	42 (21.1)	23 (23.7)	19 (18.6)
No. of steps per day, mean (SD)^f^	6244.7 (3640.6)	6236.9 (3403.4)	6252.5 (3895.6)

Abbreviations: BMI, body mass index (calculated as weight in kilograms divided by height in meters squared); REE, resting energy expenditure.

^a^ Includes Asian, Native Hawaiian or other Pacific Islander, unknown race or ethnicity, and race or ethnicity not reported.

^b^ Significant between-group differences in BMI were based on a 2-tailed *t* test at *P* < .05.

^c^ Includes data for 97 individuals in the standardized group and 101 individuals in the personalized group.

^d^ Includes data for 74 individuals in the standardized group and 81 individuals in the personalized group.

^e^ Includes data for 73 individuals in the standardized group and 81 individuals in the personalized group.

^f^ Includes data for 54 individuals in the standardized group and 54 individuals in the personalized group.

### Primary Outcome

**Figure 2.**
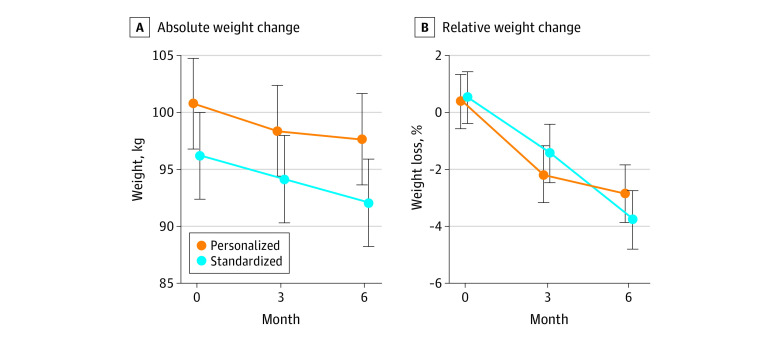
Body Weight Change Between Personalized and Standardized Arms Absolute and relative body weight change between participants randomized to the personalized group compared with the standardized group were assessed at baseline (0 months) and at 3 and 6 months. A, Data are reported as mean (95% CI). B, Estimates are from the linear mixed model and not the mean; therefore, the values are slightly different from 0.

The mean relative weight loss and percentage of weight loss from 0 to 6 months are shown in Figure 2, with highly variable weight loss within the standardized and personalized groups (eFigure 2 in Supplement 2). Weight change at 6 months was −4.31% (95% CI, −5.37% to −3.24%) for the standardized group and −3.26% (95% CI, −4.25% to −2.26%) for the personalized group, which was not significantly different (difference between groups: 1.05% [95% CI, −0.40% to 2.50%]; *P* = .16) (Table 2). However, the standardized group had significantly greater weight loss from 4 to 6 months compared with the personalized group (difference between groups: 1.66% [95% CI, 0.13%-3.18%]; *P* = .03). We found no significant difference in weight loss outcomes between the 2 groups among participants who enrolled before or after the COVID-19 pandemic (eTable 2 in Supplement 2).

**Table 2.** Results of Linear Mixed Regression Analyses: Total Change Over Time in Primary and Secondary Outcomes^a^

Outcome by analysis point, mo	Participant group				Difference of change	
	Standardized diet		Personalized diet			
	Estimate (95% CI)	*P* value	Estimate (95% CI)	*P* value	Estimate (95% CI)	*P* value
Body weight, kg						
0-3	−2.04 (−3.13 to −0.94)	<.001	−2.43 (−3.42 to −1.43)	<.001	−0.39 (−1.86 to 1.08)	.60
4-6	−2.12 (−3.25 to −1.00)	<.001	−0.71 (−1.72 to 0.29)	.17	1.41 (−0.10 to 2.93)	.07
0-6	−4.16 (−5.25 to −3.08)	<.001	−3.14 (−4.14 to −2.13)	<.001	1.02 (−0.45 to 2.50)	.17
Body weight change, %						
0-3	−1.98 (−3.04 to −0.91)	<.001	−2.58 (−3.57 to −1.60)	<.001	−0.61 (−2.06 to 0.84)	.41
4-6	−2.33 (−3.46 to −1.19)	<.001	−0.67 (−1.70 to 0.35)	.20	1.66 (0.13 to 3.18)	.03
0-6	−4.31 (−5.37 to −3.24)	<.001	−3.26 (−4.25 to −2.26)	<.001	1.05 (−0.40 to 2.50)	.16
Fat mass, kg						
0-3	−1.54 (−2.40 to −0.68)	.001	−1.40 (−2.17 to −0.61)	<.001	0.14 (−1.02 to 1.30)	.81
4-6	−0.93 (−1.91 to 0.04)	.06	−0.26 (−1.15 to 0.63)	.57	0.68 (−0.64 to 2.00)	.31
0-6	−2.48 (−3.43 to −1.51)	<.001	−1.65 (−2.53 to −0.77)	<.001	0.82 (−0.48 to 2.13)	.21
Fat-free mass, kg						
0-3	−0.91 (−1.46 to −0.36)	<.001	−0.30 (−0.81 to 0.20)	.23	0.60 (−0.14 to 1.35)	.11
4-6	−0.39 (−1.02 to 0.24)	.22	−0.21 (−0.78 to 0.37)	.49	0.18 (−0.67 to 1.04)	.67
0-6	−1.30 (−1.92 to −0.68)	<.001	−0.51 (−1.08 to 0.06)	.08	0.79 (−0.05 to 1.63)	.07
Body fat, %						
0-3	−0.74 (−1.37 to −0.11)	.02	−0.87 (−1.45 to −0.29)	.003	−0.13 (−0.98 to 0.72)	.76
4-6	−0.51 (−1.23 to 0.22)	.17	−0.13 (−0.79 to 0.53)	.70	0.38 (−0.59 to 1.36)	.44
0-6	−1.25 (−1.96 to −0.54)	.001	−1.00 (−1.65 to −0.35)	.003	0.25 (−0.71 to 1.21)	.61
Respiratory quotient						
0-3	0.04 (−0.01 to 0.09)	.14	0.01 (−0.04 to 0.06)	.57	−0.02 (−0.10 to 0.04)	.48
4-6	−0.07 (−0.13 to −0.01)	.02	−0.003 (−0.06 to 0.05)	.92	0.07 (−0.02 to 0.15)	.11
0-6	−0.03 (−0.09 to 0.02)	.26	0.01 (−0.05 to 0.06)	.67	0.04 (−0.04 to 0.11)	.26
REE, kcal/d						
0-3	−95.4 (−155.1 to −35.6)	.002	−34.6 (−89.6 to 20.4)	.22	60.8 (−20.3 to 142.0)	.14
4-6	10.5 (−59.3 to 80.3)	.77	42.0 (−21.5 to 105.5)	.19	31.5 (−62.8 to 125.9)	.51
0-6	−84.9 (−152.5 to −17.3)	.01	7.44 (−54.3 to 69.1)	.81	92.3 (0.9 to 183.8)	.05
AT, kcal/d						
0-3	−51.1 (−108.2 to 56.0)	.08	−14.6 (−66.5 to 37.3)	.58	36.5 (−40.5 to 113.7)	.35
4-6	22.8 (−45.1 to 90.8)	.51	54.1 (−6.7 to 115.0)	.08	31.3 (−59.9 to 122.5)	.50
0-6	−28.3 (−92.8 to 36.2)	.39	39.5 (−18.3 to 97.4)	.18	67.8 (−18.7 to 154.4)	.12

Abbreviations: AT, adaptive thermogenesis; REE, resting energy expenditure.

^a^ All models were adjusted for age, sex, race and ethnicity, and metformin use at baseline. Significance was set at 2-tailed *P* < .05.

### Secondary Outcomes

There were no significant differences in changes in fat mass, percentage of body fat, fat-free mass, respiratory quotient, and AT from 0 to 6 months between the 2 groups (Table 2). The linear mixed regression model indicated a significant difference in the change in REE from 0 to 6 months between the 2 groups (92.3 [95% CI, 0.9-183.8 kcal/d; *P* = .05), with a greater reduction in the standardized group compared with the personalized group.

### Adherence to Counseling Sessions and Self-monitoring

Participants in both groups demonstrated good attendance records at the WebEx counseling sessions, with no statistical difference between groups (mean [SD] percentage of sessions attended: 67.7% [29.6%] in the standardized group vs 71.3% [28.3%] in the personalized group; *P* = .40). Participants in the personalized group demonstrated higher adherence to self-monitoring by percentage of days with more than 0 kcal/d logged (mean [SD],54.3% [35.5%] vs 41.1% [38.5%]; *P* = .01) and logging at least 50% of daily caloric targets (mean [SD], 42.0% [34.2%] vs 29.9% [33.6%]; *P* = .01).

### Dietary Measures

Baseline dietary intake for the combined groups were as follows: mean (SD) energy intake was 1826 (778) kcal/d, with 44.3% (11.0%) of energy from carbohydrates, 36.0% (9.2%) of energy from total fat, and 19.7% (6.8%) of energy from protein. Dietary intake at baseline was not significantly different between the 2 groups (eTable 3 in Supplement 2). In addition, there were no between-arm differences in the changes in dietary intake measures from baseline to 6 months (eTable 4 in Supplement 2).

## Discussion

In our Personal Diet Study, we investigated the effects of a standardized low-fat diet vs a personalized diet on percentage of weight loss in 200 adults with abnormal glucose metabolism and obesity by leveraging a predictive machine learning algorithm developed by Zeevi et al.^10^ We hypothesized that the personalized group would experience greater weight loss at 6 months compared with the standardized group. Despite participants in both groups losing weight, there was no significant difference in mean percentage of weight loss, which is similar to prior evidence in low-fat and low-carbohydrate diets.^20^ Overall, weight losses did not reach a threshold considered to be clinically meaningful (eg, 5%); however, moderate weight loss of 3% to 5% has been reported to improve health outcomes.^21^ The between-group differences in secondary outcomes were all nonsignificant aside from REE, which may be the result of a greater decrease in fat-free mass.^22^ It should be noted that the between-group differences in fat-free mass were nonsignificant.

To our knowledge, no other study has implemented a machine learning algorithm to estimate PPGR in the context of a behavioral weight loss intervention, although the development of machine learning for precision nutrition is growing.^23^ The Food4Me European study^24^ used personalized feedback from baseline dietary intake data plus phenotypic and genotypic data. Despite showing improved dietary behaviors (ie, Healthy Eating Index), the investigators found no significant difference in body weight at 6 months when compared with a nonpersonalized diet group.^24^ In addition, the study by Ben-Yacov et al^14^ in 2021 found no significant difference in body weight between a postprandial targeting diet and a Mediterranean diet at 6 months. The application of a precision nutrition intervention for the purpose of weight loss may require different estimation features from the machine learning algorithm compared with those targeting a reduction in PPGR. Future studies are needed to develop and test a weight loss–specific algorithm that incorporates key characteristics central to body weight regulation as well as features of the energy balance model, including appetitive hormones (eg, leptin, glucagonlike peptide 1), total energy expenditure, and fat mass.^25^

The dietary intake data indicate a greater decrease in energy intake in the personalized group compared with the standardized group, although these findings do not support the changes in body weight observed in the standardized group. We assumed the 2 groups would differ in dietary composition, especially dietary carbohydrate intake, given the impact of carbohydrate intake on PPGR. Evidence in the study by Ben-Yacov et al^14^ among adults with prediabetes using the same machine learning algorithm found that participants randomized to a postprandial targeting diet (similar to our personalized group) significantly decreased carbohydrate intake and increased both protein and fat intake compared with those randomized to a Mediterranean diet. It is unclear whether our null findings in dietary composition are owing to methodological limitations or adherence to the interventions.

Participants in the personalized group were directed to record planned meals into the PNP app, review meal scores, and adjust dietary intakes accordingly based on predictive feedback to reduce PPGR. The complexity and burden imposed may have elicited a negative response with lower-than-expected self-monitoring adherence levels; therefore, participants in the personalized group may have experienced limited exposure to a key element of the intervention (ie, meal scores). However, acceptability of the interventions was not different between the 2 groups (eTable 5 in Supplement 2). Adherence to self-monitoring has been shown to be associated with successful weight loss.^26^ Future studies should consider collecting qualitative data to identify and mitigate barriers to self-monitoring, enrolling participants deemed adherent to self-monitoring after a run-in period, and using self-monitoring incentives.

### Strengths and Limitations

Our study has several strengths, including randomization, blinded ascertainment of outcomes, testing of an innovative and personalized intervention, repeated major points of data collection, and good adherence (71.3%) to remote counseling sessions. Despite these strengths, our study has several limitations. The COVID-19 pandemic presented a limitation to our study, resulting in discontinuation of secondary outcomes (body composition, REE, and AT) and removal of the 12-month measurements. Weight gain and poor dietary behaviors as a result of self-quarantine measures during the pandemic have been reported previously^27,28^; hence, such factors may have affected our study outcomes. To generate meal scores for the personalized intervention participants, stool samples were shipped to and processed in Israel, as well as reprogramming of the PNP app, which delayed appearance of meal scores in the PNP app. Consequently, delayed exposure to personalized counseling may have limited intervention effects. Our study sample was well educated, had an underrepresentation of men (one-third of the sample), were all English speaking, and were recruited from a single health care system, thus limiting generalizability. To minimize participant burden, dietary intake data were limited to 1 automated, self-administered, 24-hour dietary assessment collected at each measurement point in a subset of participants, which limited our ability to capture day-to-day variability in dietary intake. We were unable to detect changes in physical activity during the intervention because the use of wearable monitors to objectively capture physical activity levels was limited to baseline data only. Furthermore, participant retention was low, particularly in the standardized group. Finally, although our post hoc analyses showed no difference in weight loss before or after the COVID-19 pandemic, there may have been socioenvironmental barriers present that were not captured (eg, limitations in food supply, challenges with grocery shopping before the availability of COVID-19 vaccines).

## Conclusions

Precision nutrition directly addresses metabolic heterogeneity and may serve as a treatment for obesity and other metabolic diseases.^29^ Despite the innovative nature of this randomized clinical trial, we found that there were no significant differences in percentage of weight loss between a standard low-fat diet compared with a precision nutrition diet aimed at reducing PPGR. Given that our study is fully in line with the mission of the Nutrition for Precision Health initiative and the 2020-2030 Strategic Plan for NIH (National Institutes of Health) Nutrition Research, future interventions should examine ways to increase dietary self-monitoring adherence and intervention exposure and consider the development and testing of a weight loss–specific predictive algorithm.^30^

## Notes

Supplement 1.
Trial Protocol

Supplement 2. **eMethods.** Study Procedures and Measures **eTable 1.** Baseline Demographics, Anthropometrics, and Metabolic Variables Between Study Completers and Dropouts **eTable 2.** Group Comparisons of Weight Loss Within Participants Enrolled Before and After the COVID-19 Pandemic **eTable 3.** Baseline Dietary Intake Between the Standardized and Personalized Groups **eTable 4.** Changes in Dietary Measures From Baseline (Month 0) to 3 and 6 Months **eTable 5.** End-of-Study Questionnaire **eFigure 1.** Schematic of Study Design **eFigure 2.** Weight Loss Variability Between the Standardized (Top) and Personalized (Bottom) Groups
eReferences

Supplement 3.
Data Sharing Statement
