# Supplementary material for: Effect of a Personalized Diet to Reduce Postprandial Glycemic Response vs a Low-fat Diet on Weight Loss in Adults With Abnormal Glucose Metabolism and Obesity: A Randomized Clinical Trial
Source: JAMA Netw Open. 2022 Sep 28;5(9):e2233760. doi: 10.1001/jamanetworkopen.2022.33760 (PMC9520362; doi:10.1001/jamanetworkopen.2022.33760)
Supplement: Supplement 1. — Trial Protocol [file jamanetwopen-e2233760-s001.pdf]

NYU School of Medicine
NYU LANGONE MEDICAL CENTER

Personalized Technology-Supported Counseling to Reduce Glycemic Response in Dietary Weight Loss: The Personal Diet Study

**Principal Investigator:**	Mary Ann Sevick, ScD<br>Department of Population Health<br>NYU School of Medicine<br>180 Madison Avenue<br>New York, NY 10016<br>646-501-3427
**NYULMC Study Number:**	ss17-00741
**Funding Sponsor:**	American Heart Association<br>*122 East 42nd Street*<br>*New York, NY 10168*<br>*(212) 878-5900*
**ClinicalTrials.gov Number**	NCT03336411

Version: **9.15.20**

CONFIDENTIAL
This material is the property of the NYU School of Medicine and Langone Medical Center. Do not disclose or use except as authorized in writing by the study sponsor

Statement of Compliance

This study will be conducted in accordance with the Code of Federal Regulations on the Protection of Human Subjects (45 CFR Part 46), any other applicable US government research regulations, and institutional research policies and procedures. The Principal Investigator will assure that no deviation from, or changes to the protocol will take place without prior agreement from the sponsor and documented approval from the Institutional Review Board (IRB), except where necessary to eliminate an immediate hazard(s) to the trial participants. All personnel involved in the conduct of this study have completed Human Subjects Protection Training.

Table of Contents

PROTOCOL SUMMARY 1
SCHEMATIC OF STUDY DESIGN 3
1 KEY ROLES 4
2 INTRODUCTION, BACKGROUND INFORMATION AND SCIENTIFIC RATIONALE 8
2.1 BACKGROUND INFORMATION AND RELEVANT LITERATURE 8
2.2 RATIONALE 11
2.3 POTENTIAL RISKS & BENEFITS 12
2.3.1 Known Potential Risks *12*
2.3.2 Known Potential Benefits *14*
3 OBJECTIVES AND PURPOSE 14
3.1 PRIMARY OBJECTIVE 14
3.2 SECONDARY OBJECTIVES (IF APPLICABLE) 14
4 STUDY DESIGN AND ENDPOINTS 14
4.1 DESCRIPTION OF STUDY DESIGN 14
4.2 STUDY ENDPOINTS 6
4.2.1 Primary Study Endpoints *7*
4.2.2 Secondary Study Endpoints *7*
4.2.3 Exploratory Endpoints *7*
5 STUDY ENROLLMENT AND WITHDRAWAL 8
5.1 INCLUSION CRITERIA 8
5.2 EXCLUSION CRITERIA 8
5.3 VULNERABLE SUBJECTS 9
5.4 STRATEGIES FOR RECRUITMENT AND RETENTION 9
5.4.1 Use of DataCore/Epic Information for Recruitment Purposes *10*
5.5 DURATION OF STUDY PARTICIPATION 11
5.6 TOTAL NUMBER OF PARTICIPANTS AND SITES 12
5.7 PARTICIPANT WITHDRAWAL OR TERMINATION 12
5.7.1 Reasons for Withdrawal or Termination *12*
5.7.2 Handling of Participant Withdrawals or Termination *12*
5.8 PREMATURE TERMINATION OR SUSPENSION OF STUDY 12
6 BEHAVIORAL/SOCIAL INTERVENTION 12
6.1 STUDY BEHAVIORAL OR SOCIAL INTERVENTION(S) DESCRIPTION 12
6.1.1 Administration of Intervention *13*
6.1.2 Procedures for Training Interventionalists and Monitoring Intervention Fidelity *13*
6.1.3 Assessment of Subject Compliance with Study Intervention *13*
7 STUDY PROCEDURES AND SCHEDULE 13
7.1 STUDY PROCEDURES/EVALUATIONS 13
7.1.1 Study Specific Procedures *13*
7.1.2 Standard of Care Study Procedures *14*
7.2 LABORATORY PROCEDURES/EVALUATIONS 14
7.2.1 Clinical Laboratory Evaluations *14*
7.2.2 Other Assays or Procedures *14*
7.2.3 Specimen Preparation, Handling, and Storage *14*
7.2.4 Specimen Shipment *15*
7.3 STUDY SCHEDULE 15
7.3.1 Screening *15*
7.3.2 Enrollment/Baseline *16*

7.3.3 Intermediate Visits *17*
7.3.4 Final Study Visit *17*
7.3.5 Withdrawal Visit *18*
7.3.6 Unscheduled Visit *18*
7.4 Concomitant Medications, Treatments, and Procedures 18
7.5 Justification for Sensitive Procedures 18
7.5.1 Precautionary Medications, Treatments, and Procedures *18*
7.6 Prohibited Medications, Treatments, and Procedures 18
7.7 Prophylactic Medications, Treatments, and Procedures 18
7.8 Participant Access to Study Intervention at Study Closure 18

8 ASSESSMENT OF SAFETY 19

8.1 Specification of Safety Parameters 19
8.1.1 Definition of Adverse Events (AE) *19*
8.1.2 Definition of Serious Adverse Events (SAE) *19*
8.1.3 Definition of Unanticipated Problems (UP) *19*
8.2 Classification of an Adverse Event 19
8.2.1 Severity of Event *19*
8.2.2 Relationship to Study Intervention *20*
8.2.3 Expectedness *20*
8.3 Time Period and Frequency for Event Assessment and Follow-Up 20
8.4 Reporting Procedures – Notifying the IRB 21
8.4.1 Adverse Event Reporting *21*
8.4.2 Serious Adverse Event Reporting *21*
8.4.3 Unanticipated Problem Reporting *21*
8.4.4 Reporting of Pregnancy *22*
8.5 Reporting Procedures – Notifying the Study Sponsor 22

8.6 Study Halting Rules 22
8.7 Safety Oversight 22

9 CLINICAL MONITORING 23

10 STATISTICAL CONSIDERATIONS 23

10.1 Statistical and Analytical Plans 23
10.2 Statistical Hypotheses 23
10.3 Analysis Datasets 23
10.4 Description of Statistical Methods 23
10.4.1 General Approach *23*
10.4.2 Analysis of the Primary Efficacy Endpoint(s) *24*
10.4.3 Analysis of the Secondary Endpoint(s) *24*
10.4.4 Safety Analyses *24*
10.4.5 Adherence and Retention Analyses *24*
10.4.6 Baseline Descriptive Statistics *25*
10.4.7 Planned Interim Analysis *25*
10.4.8 Additional Sub-Group Analyses *25*
10.4.9 Multiple Comparison/Multiplicity *25*
10.4.10 Tabulation of Individual Response Data *25*
10.4.11 Exploratory Analyses *25*
10.5 Sample Size 25
10.6 Measures to Minimize Bias 26
10.6.1 Enrollment/Randomization/Masking Procedures *26*
10.6.2 Evaluation of Success of Blinding *26*
10.6.3 Breaking the Study Blind/Participant Code *26*

11 SOURCE DOCUMENTS AND ACCESS TO SOURCE DATA/DOCUMENTS 26

12 QUALITY ASSURANCE AND QUALITY CONTROL 27

13 ETHICS/PROTECTION OF HUMAN SUBJECTS 27
13.1 Ethical Standard 27
13.2 Institutional Review Board 27
13.3 Informed Consent Process 27
13.3.1 Consent/Assent and Other Informational Documents Provided to Participants 27
13.3.2 Consent Procedures and Documentation 28
13.4 Participant and Data Confidentiality 28
*13.4.1 Research Use of Stored Human Samples, Specimens, or Data**Error! Bookmark not defined.***
13.5 Future Use of Stored Specimens .... **Error! Bookmark not defined.**
14 DATA HANDLING AND RECORD KEEPING 29
14.1 Data Collection and Management Responsibilities 29
14.2 Study Records Retention 29
14.3 Protocol Deviations 30
14.4 Publication and Data Sharing Policy 30
15 STUDY FINANCES 31
15.1 Funding Source 31
15.2 Costs to the Participant 31
15.3 Participant Reimbursements or Payments 31
16 STUDY ADMINISTRATION 31
16.1 Study Leadership 31
17 CONFLICT OF INTEREST POLICY 31
18 REFERENCES 32
19 ATTACHMENTS 36

List of Abbreviations

AE	Adverse Event/Adverse Experience
AGE	Advanced Glycation End products
AUC	Area Under the Curve
BIA	Bioelectrical Impedance Analysis
BMI	Body Mass Index
CBC	Complete Blood Count
CFR	Code of Federal Regulations
CGM	Continuous Glucose Monitoring
CRF	Case Report Form
CSOC	Clinical Study Oversight Committee
CTSI-CRC	Clinical and Translational Science Institute-Clinical Research Center
CVD	Cardiovascular Disease
DCC	Data Coordinating Center
DHHS	Department of Health and Human Services
DSMB	Data and Safety Monitoring Board
FFR	Federal Financial Report
FWA	Federalwide Assurance
GCP	Good Clinical Practice
GFR	Glomerular Filtration Rate
GI	Glycemic Index
GL	Glycemic Load
GV	Glycemic Variability
HbA1c	Glycosylated Hemoglobin
HIPAA	Health Insurance Portability and Accountability Act
ICF	Informed Consent Form
IRB	Institutional Review Board
ISM	Independent Safety Monitor
MOP	Manual of Procedures
N	Number (typically refers to participants)
NIH	National Institutes of Health
OHRP	Office for Human Research Protections
OHSR	Office of Human Subjects Research
PNP	Personalized Nutrition Program

PI	Principal Investigator
QA	Quality Assurance
QC	Quality Control
RAGE	Receptor for Advanced Glycation End products
REE	Resting Energy Expenditure
SAE	Serious Adverse Event/Serious Adverse Experience
SCT	Social Cognitive Theory
SOP	Standard Operating Procedure
sRAGE	Soluble Receptors for Advanced Glycation End products
TP	Treating Physician
US	United States
T2D	Type 2 Diabetes

Protocol Summary

Title	Personalized Technology-Supported Counseling to Reduce Glycemic Response in Dietary Weight Loss: The Personal Diet Study
Short Title	The Personal Diet Study
Brief Summary	The aim of this 2-phase, randomized clinical trial will be to examine the effects of two behavioral weight loss interventions on weight loss. This study will be conducted in 210 overweight or obese pre-diabetic and early-stage diabetic individuals recruited from community-based settings. Phase 1 will include 6-months of active intervention. Phase 2 will consist of 6-months of maintenance and observation. Measurements will occur at screening, baseline, 3, and 6 months, and 12 months for a subgroup of participants for which data collection will end in October 2020. Participants will be randomized with equal allocation to 2 groups: (1) a standardized behavioral weight loss intervention with a one-size-fits-all regimen that includes counseling about restriction of calories and calories from fat, and physical activity, delivered using mHealth technology, (hereafter *mHealth*), or (2) all of the elements of *mHealth*, plus personalized dietary recommendations to minimize glycemic response to meals (hereafter *personalized-mHealth*). Participants will be required to attend 6 separate visits over both phases of the study. All data collection and measurements for the last 3 cohorts of this study will be conducted remotely starting October 2020.
Phase	1
Objectives	**1. Primary objective.** At 6-months we will examine the impact of *mHealth* and *Personalized-mHealth* on weight loss‡.<br>**2. Secondary objective 2.a-2.c**: We will describe differences between *mHealth* and *personalized-mHealth* participants in terms of **2.a** body composition, and **2.b** metabolic adaptation at 6 months, and 12 months for a subgroup of participants for which data collection will end in October 2020, and **2.c** weight regain at 12 months for a subgroup of participants for which data collection will end in October 2020.<br>**3. Secondary objectives 3.a-3.d:** We will describe the mediating effect of **3.a** self-efficacy on the relationship between randomization assignment and weight loss/regain at 6 and 12 months. We will describe the mediating effect of changes in **3.b** GV (6 months only), **3.c** RAGE/AGE/S100A8/A9 (6 and 12 months), and **3.d** inflammatory markers (6 and 12 months) on body composition and metabolic adaptation within and between treatment groups.
Methodology	2-Phase, 2-group Randomized Clinical Trial
Endpoint	Primary endpoint: weight (0, 6, 12 mos)<br>Secondary endpoint: body composition (0, 6, 12 mos); metabolic adaptation (0, 6, 12 mos)<br>Mediator endpoint: self-efficacy, glycemic variability, circulating mediators of inflammation, adiopokines, WBC, platelets
Study Duration	34 months
Participant Duration	Total time: 10 hours

‡In March 2020, all in-person research visits were halted due to COVID-19. Data collection for the primary outcome was transitioned to remote collection via participants' home scale. This will be taken into account during the final analysis and is not anticipated to have a significant effect on the primary outcome.

Duration of behavioral intervention	6 months
Population	males and females, 18-80 yo, healthy overweight or obese pre-diabetic or early T2D individuals, BMI 27-50 kg/m^2
Study Sites	NYU's Clinical and Translational Science Institute (CTSI)
Number of participants	280 total participants (210 randomized) projected
Description of Study Intervention/Procedure	Behavioral weight loss intervention with personalized dietary recommendations based on machine learning algorithm that integrates gut microbiota, dietary intake, physical activity and various blood parameters to predict postprandial glycemic response
Reference Therapy	Behavioral weight loss intervention with a one-size-fits-all approach
Key Procedures	Blood draws, height, weight, REE, BIA, waist, hip, and neck circumference, stool samples, CGM, Fitbit, PNP
Statistical Analysis	Statistical modeling will be based on baseline, 36 and 12-month outcome variables using linear mixed models. 12 month outcome variables will be used for exploratory analysis in a subgroup of participants. Descriptive analysis of all data collected will be performed using appropriate graphical and numerical exploratory data techniques. An "intent-to-treat" approach will be use dot address specific aims. Fixed effects will include: recruitment site, presence/absence of T2D, time and intervention. Regression diagnostics will be used for model assessment, and post hoc multiple comparisons tests (i.e., Tukey or Tukey-Kramer) will be applied to adjust for pairwise comparisons. Primary and secondary analyses will us Stata and SAS, and MPlus in mediation analyses.

CONFIDENTIAL
This material is the property of the NYU School of Medicine and Langone Medical Center. Do not disclose or use except as authorized in writing by the study sponsor

Schematic of Study Design

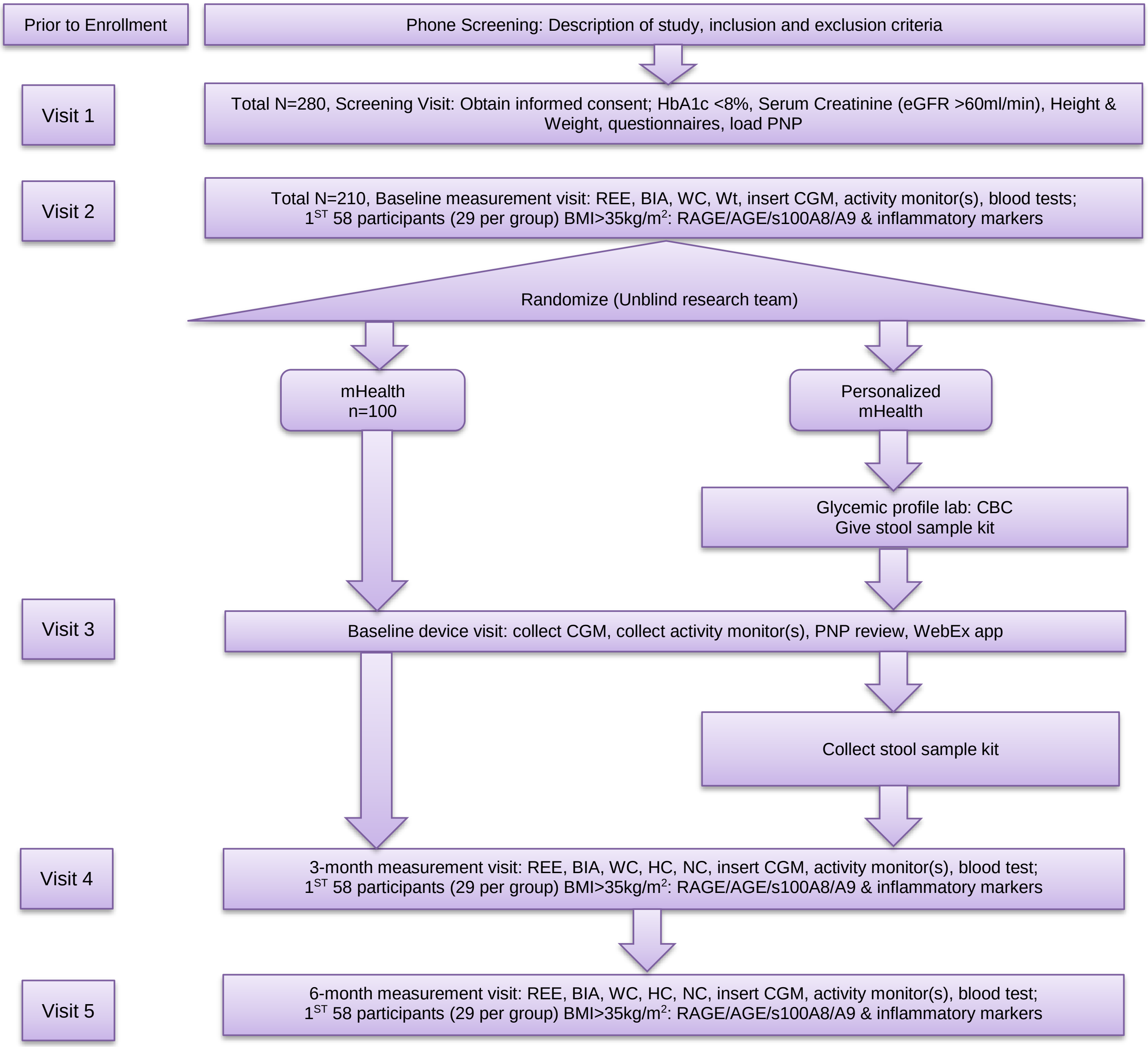

CONFIDENTIAL
This material is the property of the NYU School of Medicine and Langone Medical Center. Do not disclose or use except as authorized in writing by the study sponsor

1 Key Roles

Mary Ann Sevick, ScD (Principal Investigator)
Department of Population Health
NYU School of Medicine
180 Madison Avenue, New York, NY 10016
646-501-3427
mary.sevick@nyulangone.org

Dr. Sevick is a Professor in the Department of Population Health in the School of Medicine at New York University, and Director of the mHealth Unit. Over the past 25 years, Dr. Sevick has had experience with a variety of large clinical trials involving behavior change strategies including the Women's Health Initiative, the Activity Counseling Trial; the Arthritis, Diet & Activity Promotion Trial; the Reconditioning & Exercise for COPD Trial; and Applications for Lifestyle Exercise study. Her primary interest is in the area of chronic illness and she has recently been involved, as Principal Investigator, in several studies to examine adherence to disease management regimens. Dr. Sevick was PI on the ENHANCE Study (NIH-R01-NR008792), a clinical trial to evaluate a lifestyle intervention paired with technology-based self-monitoring in those with type 2 diabetes – the behavioral intervention on which the proposed study is based. Dr. Sevick recently migrated the ENHANCE intervention approach (which used PDAs) to one that employs mobile technology and has implemented this in the ongoing Diabetes Healthy Hearts and Kidneys Study (R01-DK10049). These studies uniquely prepare her to conduct the proposed study. Dr. Sevick has extensive experience implementing clinical interventions that employ technology in clinical populations. She will head the Steering Committee, and Measurement, Recruitment, and Intervention Subcommittees, and will directly supervise the project manager and staff.

Michael Bergman, MD, (Co-Investigator)
NYU School of Medicine
Diabetes & Endocrine Associates
530 1st Avenue, Suite 5E
New York, NY 10016
212- 481-1350
Michael.bergman@nyulangone.org

Dr. Michael Bergman is a Clinical Professor of Medicine at New York University School of Medicine. He is a senior Endocrinologist, with 39 years of experience managing patients with diabetes. In addition to being a master clinician, he is Acting Section Chief of the Endocrinology, Diabetes, and Metabolism at the Manhattan VA New York Harbor Healthcare System. He also is Director of the NYU Diabetes Prevention Program. Dr. Bergman will guide the investigative team in developing the recruitment strategy, and assist with interpretation of clinical data. He will provide medical oversight to the study.

Huilin Li, PhD, (Co-investigator)
Department of Population Health
NYU School of Medicine
650 1st Avenue, RM 547
New York, NY 10016
212-263-8977
huilin.li@nyulangone.org

Dr. Li is an Assistant Professor of Biostatistics in the Department of Population Health at New York University School of Medicine. She has extensive expertise in statistical methodology developments and biomedical applications, including genetic analysis, microbiome data analysis, cancer epidemiology analytical methods, spatial analysis and survey methodology. Particularly relevant to this application, Dr. Li collaborates on studies of the mechanisms by which diabetes accelerates atherosclerosis via RAGE and currently is the leading biostatistician for the NYU Microbiome Lab Group (PI: Dr. Martin Blaser) on studying the function of the human microbiome in infectious diseases and obesity. She is current co-I on Dr. Sevick's Diabetes Healthy Hearts

and Kidneys Study. Dr. Li will provide support for data management, study design, and data analyses; be responsible for interim analyses, necessary modification of design, preparation of required documentation and analysis summaries for progress reports and publications.

Antoinette Schoenthaler, EdD, MA (Co-Investigator)
Department of Population Health
NYU School of Medicine
180 Madison Avenue, New York, NY 10016
212-263-4205
antoinette.schoenthaler@nyulangone.org

Dr. Schoenthaler is an Assistant Professor of Medicine, with content expertise in Health Education and Behavior and a research trajectory pertaining to racial disparities, adherence, and cardiovascular health. Her research focuses on adherence decision-making with regard to prescribed medications and lifestyle behaviors, with emphasis on psychosocial factors such as depression, self-efficacy, and intrinsic motivation. Dr. Schoenthaler has expertise in the administration of treatment fidelity measures, assessment of psychosocial measures and lifestyle changes. She leads motivational interviewing trainings and coaching sessions for community health workers and staff members. She will guide the behavioral component of the study, train the interventionists in behavioral methods, and implement methods to assure intervention fidelity.

Lu Hu, PhD, RN
Department of Population Health
NYU School of Medicine
180 Madison Avenue, New York, NY 10016
646-501-2602
lu.hu@nyulangone.org

Dr. Hu is a fellow at the Center for Healthful Behavior Change and was involved in the development of this intramural application and in the design of the 3 program projects. She is a nurse by training, with expertise pertaining to the use of mobile technology in the clinical management of patients with chronic disease. She will assist the investigators with implementation of recruitment, measurement, and intervention protocols. She also will assist the PI in the development of the larger P01 application. She will be fully covered by internal resources and no salary support is required.

David St. Jules, PhD, RD
Department of Population Health
NYU School of Medicine
180 Madison Avenue, New York, NY 10016
646-501-2624
david.stjules@nyulangone.org

Dr. St. Jules is an Assistant Professor at the Center for Healthful Behavior. He has expertise in clinical nutrition and nutritional epidemiology. He was involved in the development of the dietary measurement and validation components of this application. He will guide the feeding study component of the study, including generation of standardized menus, oversight of the metabolic kitchen meal preparation, and quantifying adherence to feedings.

Collin Popp, PhD, RD
Department of Population Health
NYU School of Medicine
180 Madison Avenue, New York, NY 10016
collin.popp@nyulangone.org

Dr. Popp is a fellow at the Center for Healthful Behavior Change. His background is in physiology, exercise physiology and physical activity, and is also a Registered Dietitian. He will assist the investigators with implementation of recruitment, measurement, and intervention protocols.

Lisa Ganguzza, MS RD CDN (Project Manager)
Department of Population Health
NYU School of Medicine
180 Madison Avenue, New York, NY 10016212-263-4171
lisa.ganguzza@nyulangone.org

Lisa is a licensed Registered Dietitian with a BA in Communication, and a MS in Clinical Nutrition. Lisa will be responsible for overseeing research staff on screening, recruitment, and consent.

Mary Lou Pompeii, RDN CSR CDN CDE
Department of Population Health
NYU School of Medicine
180 Madison Avenue, New York, NY 10016
646-501-3468

Mary Lou is a research dietitian with training in behavioral methods. She is working with PI on other diet-related studies, and will contribute to recruitment, study visit, and intervention questions.

William Coleman, MA (Research Data Associate)
Department of Population Health
NYU School of Medicine
180 Madison Avenue, New York, NY 10016
646-501-3463
William.coleman@nyulangone.org

William is a research data associate at the Center for Healthful Behavior Change. His academic background is in general psychology (BA/MA), including research methods and experience. He will assist staff with mobile technology set-up and monitoring, basic web development, data management processes, and descriptive data analysis.

Margaret Curran, BS (Research Data Associate)
Department of Population Health
NYU School of Medicine
180 Madison Avenue, New York, NY 10016
646-501-3459
Margaret.curran@nyulangone.org

Margaret has a BS in psychology with a concentration in neuroscience with training in clinical nutrition. She will be responsible for screening, recruitment, and consent. She will attend CTSI-CRC baseline measurement visits, during which he will collect and process stool samples for shipment, administer questionnaires, obtain height and weight, coordinate collection of blood, perform indirect calorimetry, and instruct participants in wearing the Fitbit and use of PNP app to record meals, stress, sleep, diet and medications. Margaret will also be aiding the research dietitian in participant contact and performance feedback.

Paige Illiano, RD
Department of Population Health
NYU School of Medicine
180 Madison Avenue, New York, NY 10016
646-501-3470
Paige.Illiano@nyulangone.org

Paige Illiano is a Registered Dietitian with training in behavioral methods who will serve as the study interventionist. Paige will work with the PI to develop and refine intervention materials. Paige will conduct the one-on-one sessions with participants following randomization, and will train participants in use of the PNP

app. Paige will also assist in recruitment efforts, attend CTSI-CRC measurement visits, and review participant entries in the PNP app.

Department of Population Health, NYULMC: The Department of Population Health, within NYU School of Medicine, aims to integrate, support, and advance NYULMC's contributions to population health research and related disciplines, providing a vibrant departmental home for the "bedside-to-population" as well as the "population-to-discovery" domains of translational research. It provides an academic base for efforts to integrate research into NYU's expanding health care delivery system that transcends any particular school, department, or division. The Department is a research and training hub that brings together researchers in nursing, medicine, psychology, epidemiology, biostatistics, health services and policy, behavior change, comparative effectiveness, medical ethics, prevention, and related disciplines, affording a unique and collaborative environment focused on improving the health of populations. The Department was initiated in January 2012. A core focus of the Department is improving health outcomes through innovative interventions as well as in enhancing the impact of interventions already known to be effective through their more effective implementation and dissemination. Collaboration with key public sector stakeholders and with community partners is central to the Department's mission. As researchers, faculty are engaged in dual roles: building cutting-edge science in their areas of inquiry, and providing collaborative consultation to other investigators throughout the NYULMC academic community as well as across the University. Resources within the Department for this proposal include the use of over 5,000 square feet of dedicated office space located on the newly renovated 6th floor of the NYULMC Translational Research Building, telecom (phone, fax and LAN connections), administrative assistants, and grant, regulatory (IRB) and finance administrators.

Center for Healthful Behavior Change, NYULMC: The Center for Healthful Behavior Change (CHBC) is located within the Department of Population Health. The mission of CHBC is to become a national leader in translational behavioral medicine, research, training, and education. The CHBC works toward this mission through the development, implementation, and dissemination of innovative evidence-based behavioral interventions in routine clinical practice and community-based settings with the long-term goal of disseminating effective strategies nationally and internationally. The Center is comprised of core research faculty members with expertise in various fields relevant to translational behavioral research. Faculty engage in research pertaining to heart disease, hypertension, chronic kidney disease, diabetes, cancer, health disparities research, community-based participatory research, health psychology, behavioral informatics, and health education and counseling.

Unit for Behavioral Informatics, NYULMC: The Unit for Behavioral Informatics (UBI) Research is a new unit within the CHBC, initiated in August 2013 under the direction of Dr. Sevick. UBI is the NYULMC academic home for applied researchers who integrate behavioral sciences, health sciences, health service delivery, and health information technology for the purpose of understanding, preventing, diagnosing or addressing behavioral risk factors for and consequences of chronic disease. Initiated in August of 2013, the overarching goal of the Unit is to provide training opportunities and infrastructure support for programs of research involving the use of technology in health behavior research, and translation of behavioral intervention research into clinical practice as well as community-based settings. UBI consists of 3 program areas: Behavioral Management, Measurement, and Education. The Behavioral Management Program aims to develop and evaluate tailored, eHealth applications to address behavioral risk factors for disease including diet, physical activity, addiction, violence prevention, and adherence to chronic disease self-management regimens. The Measurement Program aims to develop technological applications for and programs of research pertaining to collection of ecologically valid data (e.g. measurement of behaviors, behavioral risk factors, and related psychometric [e.g. pain] and biophysiologic [e.g. blood pressure] outcomes. The Education Program aims to develop, test, and implement of technology-based instructional programs (e.g. virtual worlds), and videos (e.g. childbearing & childrearing, prevention of vector-borne diseases, water sanitation) for populations with limited access to health care or disparate health outcomes.

NYU Langone Medical Center Endocrinology Faculty Group Practice (NYULMC Endocrinology FGP): The Department of Medicine of NYU Langone Medical Center is among the longest established in the U.S. and is the largest academic department in the NYU School of Medicine. It supports and oversees 10 subspecialty division inclusion the Division of Endocrinology, Diabetes and Metabolism

This material is the property of the NYU School of Medicine and Langone Medical Center. Do not disclose or use except as authorized in writing by the study sponsor

Smilow Research Building and Laboratory Space: This space contains multiple bay areas set up by Principal Investigator individual team, along with 8 individual walled-off rooms designated for equipment and animal surgery on site. Core equipment within Smilow includes services such as confocal microscopy, Nikon inverted microscope, bacterial shakers, dark room and X-omat for film development, facilities for ChIP and Affymetrix arrays, Biacore for surface Plasmon resonance studies, and beta/gamma counters. All of the needed equipment and expertise for the outlined studies is available at NYU. The laboratory has >10 Dell / Macintosh computers with scanner, printer, CD ROM, as well as connection to the Internet; additional computers are attached to specific equipment. Dr. Schmidt has personal laptops for use in preparation of grants, manuscripts, and correspondence. Equipment includes HPLC (Agilent), FPLC, PCR machines, Vmax ELISA reader, phosphoimager, Speed Vac, UV-VIS spectrophotometers, spectrofluorimeter (PTI), centrifuges, cold room, tissue culture facility, freezers/refrigerators, Beckmann Coulter DTX 880 fluorimetric plate reader, luminometer, centrifuges, electrophoresis systems, gel drier, facilities for the use of radioactivity, Amaxa electroporator. Microscopes include Zeiss AXIOSKOP microscope with video attachment and image analysis software, 2 Leica microscopes with attached video camera and image analysis software. An hypoxia environmental chamber is available (Biospherix). A dedicated room has been set up for onsite animal terminal sacrifice work or survival surgery as indicated; this includes Leica surgical microscope, Isoflurane machine, and the equipment needed for animal warming. Two isolated heart perfusion systems with ADI systems for cardiac function monitoring are available. There is a separate room for tissue processing equipped with facilities for fixing, embedding and cutting sections (Tissue Tek system) for light and electron microscopy, as well as a cryomicrotome).

Division of Biostatistics NYULMC: Dr. Li's office is in the Division of Biostatistics, Department of Population Health, NYU School of Medicine at 650 First Avenue, a short walk from Dr. Sevick's office. Dr. Li has access to state-of-the-art computing facilities that include a central server (Dell PowerEdge 2500) and a Sun Server. Statistical software available either on the network or the desktop includes SAS, SPSS, S-Plus (including Spatial Stats and Environmental Statistics Modules), R, PASS and Matlab.

2 Introduction, Background Information and Scientific Rationale

2.1 Background Information and Relevant Literature

Obesity is a common, costly condition and a risk factor for metabolic diseases and their complications. Data from the National Health and Nutrition Examination Survey from 1999-2010 show that 2 in 3 US adults are overweight or obese, 1 in 3 US adults is obese, and 1 in 20 are considered to be extremely obese [1]. Obesity and related metabolic diseases (especially diabetes, cardiovascular disease, hypertension, and non-alcoholic hepatosteatosis) are major contributors to disability, death, and health care costs. The annual health care costs of obesity-related illnesses were estimated in 2012 to be $190.2 billion[2].

Visceral adiposity correlates with insulin resistance, which is a precursor for the development of T2D[3]**.** Once T2D develops, obesity complicates the management of the disease by increasing insulin resistance and glucose intolerance. This, in turn, reduces the effectiveness of diabetes drugs for controlling glycemia, with poor glycemia management increasing the risk of downstream vascular complications.

Weight loss reduces the incidence of T2D and has multiple benefits in those with diagnosed T2D. The Diabetes Prevention Program demonstrated that over an average follow-up of 2-8 years, intensive lifestyle intervention (targeting a weight loss of at least 7% and 150 minutes/week of moderate intensity physical activity) reduced the incidence of T2D by 58%, compared to 31% in those receiving metformin[4]. The Look AHEAD study, in overweight or obese individuals with T2D showed that a modest weight loss of 5-10% was associated with significant improvements in CVD risk factors at 1 year, with larger weight losses resulting in even greater benefits[5]. The lifestyle intervention reduced hepatic steatosis and incident nonalcoholic fatty liver disease at 1 year[6] and improved fitness and glycemic control at 4 years. On longer-term follow-up, the Look AHEAD intervention was associated with a 31% reduction in the risk of advanced kidney disease, a 14% reduction in the risk of diabetic nephropathy, a reduced incidence of depression and sleep apnea, and reductions in required diabetes medications[7-10]. Other studies have shown weight loss in those with T2D improves glycemic control[11, 12], fitness[13], and erectile capacity[14].

CONFIDENTIAL
This material is the property of the NYU School of Medicine and Langone Medical Center. Do not disclose or use except as authorized in writing by the study sponsor

Minimization of postprandial hyperglycemic peaks may be a valuable adjuvant to dietary weight loss interventions. The causes of and intervention targets for preventing and managing obesity are topics of considerable debate[15, 16]. According to the *Insulin-Carbohydrate model* (see Figure 1 below) hyperglycemic excursions result in an exaggerated insulin response, and a subsequent drop in blood sugar that stimulates hunger. Also according to this model, the exaggerated insulin response suppresses fat mobilization, leading to a vicious cycle of fat storage and insulin resistance[17]. Proponents of the *Insulin-Carbohydrate model* suggest that minimization of postprandial hyperglycemic peaks may be a valuable adjuvant to dietary weight loss interventions[16]

In addition to being beneficial for weight loss, minimizing glycemic excursions may reduce downstream disease risk. Some (but not all[§]) clinical trials have shown that lowering postprandial hyperglycemic peaks with medication reduces the risk of progression to diabetes, hypertension, and cardiovascular events in those with impaired glucose tolerance[19, 20] and reduces carotid intima-media thickness both in those with impaired glucose tolerance[21] and T2D[22].

Figure 1. Putative relationships between postprandial glycaemia and risk factors for obesity, diabetes and cardiovascular disease. FFA, free fatty acid[18].

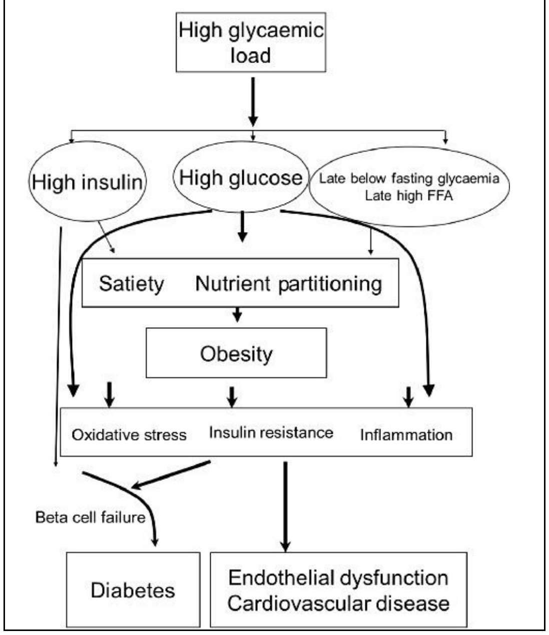

Postprandial glycemic response is driven by carbohydrates and varies considerably with the type of food consumed. In 1980, Jenkins et al. introduced the concept of glycemic index (GI), a numerical food classification system for describing the speed with which the carbohydrates are broken down during digestion and absorbed into the bloodstream[23]. The GI is the incremental area under the blood glucose response curve (AUC) to 50g of a particular food, following a 12-hour fast, divided by the AUC of a test food (usually bread or glucose) and multiplied by 100. The GI allows for the ranking of foods in terms of expected postprandial glycemic response. Glycemic load (GL) is based on the GI, but also accounts for serving size. GL is calculated by multiplying the available carbohydrate in a serving of food by the food's GI, and dividing by 100. Low-GI foods (meats, milk, beans, nuts, whole grains, most fruits and vegetables), and low-GL meals featuring low-GI foods are expected to result in more limited postprandial glycemic responses; whereas high-GI foods (sugars, refined grains, and white potatoes), and high-GL meals are expected to result in high postprandial glycemic responses.

Evidence of the efficacy of low-carbohydrate and low-GL diets for weight loss is mixed. Blaak et al. summarize multiple studies investigating the influence of GI and GL on satiety, blood glucose, insulin response, and body weight control[18]. They found that, while there is mechanistic evidence linking postprandial glycemia to weight, the results of intervention studies that manipulate GI or GL for the purpose of weight loss are mostly negative[18]. Jensen et al., in their summarization of dietary weight loss interventions concluded that, in comparison to higher carbohydrate/lower protein or lower fat diets, carbohydrate restricted diets did not result in greater weight losses. They also noted that there was insufficient evidence to comment on weight loss interventions involving complex versus simple carbohydrates; glycemic load dietary approaches; Mediterranean style, vegetarian, and other dietary pattern approaches; meal replacement; and very-low-calorie diets[24]. However, as Freedhoff and Hall note that…[25]

[§] Contrary to these findings, the Heart2D study (in which T2D patients with a history of myocardial infarction were randomized to insulin targeting postprandial versus fasting/interprandial hyperglycemia) showed that reductions in GV did not reduce cardiovascular events. However, as with ADVANCE, ACCORD, and VADT, given the pre-existing cardiovascular disease in Heart2D and the possible role of metabolic memory, it may have been too late to modify the course of the disease. The proposed study will focus on those with recently diagnosed T2D.

This material is the property of the NYU School of Medicine and Langone Medical Center. Do not disclose or use except as authorized in writing by the study sponsor

"…nevertheless, and hearteningly, anecdotal long-term diet success stories abound for most dietary approaches, and focusing on mean bodyweight trajectories masks the high individual weight loss variability within each diet group. The question is: why are some individuals more successful than others?"(p.850)

One-size-fits-all weight loss interventions that focus on glycemic load may fail to manage postprandial hyperglycemia because individuals vary greatly in their glycemic response to food[26-28]. Recent studies have shown that the composition and function of the intestinal microbiota are critical factors in glucose homeostasis[29]. In a series of mouse gut microbiota studies, Turnbaugh et al[30] demonstrated that the obese microbiome has an increased capacity to harvest energy from the diet. Others have shown the composition and function of gut microbiota to be associated with glucose intolerance[31, 32], insulin resistance[33], and T2D[34, 35] and Vrieze et al[36] showed that transfer of intestinal microbiota from lean humans to those with metabolic syndrome increased insulin sensitivity. A recent NIH Working Group Report suggests that the microbiome may play an important role in the etiology of obesity but notes that clinical studies must be performed[37].

Despite its importance, until recently no methods were available for using gut microbiome data to predict glycemic response to food. Dr. Eran Segal recently devised the first personalized algorithm for predicting glycemic response to food. The algorithm is based on a multi-dimensional profile, integrating data regarding microbiota composition, blood tests, questionnaires, and a one-week monitoring of food intake, glycemia, sleep, and physical activity in 800 participants of the Personal Nutrition Project (hereafter PNP; www.personalnutrition.org). The investigators validated these predictions in an independent 100-person cohort. In a 3-month randomized trial in people with prediabetes, Segal's group then used this algorithm to tailor dietary recommendations and successfully reduced GV. In this study, we will use the PNP algorithm to tailor intervention counseling according to the individual participant's predicted glycemic response to specific foods. We hypothesize that weight losses will be greater in a personalized low glycemic response diet than a standardized weight loss intervention that features counseling regarding restriction of calories and calories from fat, and increased physical activity.

A personalized low glycemic response diet may better support weight loss maintenance. Changes in body composition and metabolic adaptation are associated with weight loss and result in substantial decreases in energy expenditure. These changes make sustained weight loss difficult[38]. A diet that minimizes postprandial glycemic response is theorized to help preserve lean body mass and limit the degree of metabolic adaptation occurring during weight loss[39], which could facilitate weight loss maintenance.

A personalized, low glycemic response diet may contribute to self-efficacy and subsequent weight loss success in ways that cannot be achieved through standard behavioral weight loss counseling alone. Decades of research suggest that behavioral methods are essential for engaging people in lifestyle behavior change, including weight loss. The behavioral component of both *mHealth* and *Personalized-mHealth* will be based on Bandura's Social Cognitive Theory (SCT) [40, 41]. SCT focuses on the role played by self-referent thought in the maintenance of behavior change. Within SCT, behavior change and maintenance are strongly influenced by the individual's perceived self-efficacy, which is defined by Bandura as a belief in one's capabilities to successfully overcome the demands of a situation in order to achieve a desired outcome. Self-efficacy influences multiple aspects of behavior change including: adoption of a new behavior, the inhibition and disinhibition of existing behaviors, as well as adherence to self-care and self-monitoring regimens. Self-efficacy influences motivation to engage in behavior change. Those with high levels of self-efficacy tend to exert more effort, approach more challenging tasks, and persist longer in the face of obstacles and barriers. We assert that metabolic profiling to tailor dietary counseling may contribute to self-efficacy in ways that cannot be achieved through standard behavioral methods alone. Compared to *mHealth* participants, *personalized-mHealth* participants who are armed with food-specific recommendations tailored to their metabolic profile will be more likely to succeed. An enhanced sense of mastery will result in greater levels of self-efficacy and improved adherence to lifestyle change, and enhance the success of weight loss maintenance.

New technologies permit intervention in the context within which behavior occurs, and enhance access to behavioral weight loss counseling. When health care professionals counsel patients about lifestyle, they typically do so during limited encounters in clinical practice settings and depend upon patient recall of contributing behaviors and contextual factors. However, recalls often are biased, behavior is context-dependent, and interventions delivered in research and clinical practice settings may not translate to the real-life situations experienced by patients. Efforts to engage patients in lifestyle behavior change are less likely to be effective if delivery of the intervention is not temporally consistent with the occurrence of the target behavior.

This material is the property of the NYU School of Medicine and Langone Medical Center. Do not disclose or use except as authorized in writing by the study sponsor

Technology-based self-monitoring of lifestyle behavior and, immediate feedback about dietary intake (one-size-fits-all or personalized), *delivered in the context in which behaviors occur* is consistent with the theories of Ecological Momentary Intervention (EMI, i.e., interventions that occur in the natural environment, at specifically identified *moments* in everyday life such as mealtimes) and Ecological Momentary Assessment (EMA, i.e., real-time collection of data regarding the targeted behavior rather than reliance on recall)[42]. Recent reports indicate that EMI and EMA methods are efficacious for addressing a variety of health behaviors[43] and that EMI and EMA methods are feasible in a variety of patient populations[44]. In addition to providing in-the-moment intervention, mHealth has the capacity to transform the health care system by making health care services, including behavioral interventions, more accessible to patients and by permitting feedback to be tailored to real-time dietary choices (e.g., via the PNP app as described below).

The roles of receptor for advanced glycation end product (RAGE) and inflammation on study outcomes. Advanced glycation end products (AGEs) are the products of an irreversible, non-enzymatic bonding of carbohydrates such as glucose to proteins, lipids and nucleic acids, and have been implicated in aging, as well as in the development and progression of metabolic disease and its complications[45]. AGEs form and accumulate at an accelerated rate in the presence of hyperglycemia, including acute glucose fluctuations (i.e., GV), which are associated with oxidative stress[46].

The harmful effects of AGEs appear to be partly mediated through their binding to the receptor for advanced glycation end products (RAGE), which generates oxidative stress and inflammation, and mediates the tissue damage observed in the macro- and microvascular complications of metabolic disease[47, 48]. RAGE may serve as a “brake” to weight loss and predispose participants to weight regain via metabolic adaptation. RAGE also may negatively impact downstream weight loss/maintenance through the mediating effects of inflammation on energy metabolism.

Finally, soluble RAGEs (sRAGE) serve as endogenous RAGE ligand-sequestering molecules, interfering with the ability of the RAGE ligands to activate the cell surface receptor – blocking the ability of RAGE to brake energy expenditure, thereby facilitating weight loss. Consequently, we expect that higher GV may be associated with higher AGEs, and may be associated with lower sRAGE, and increased leukocyte RAGE activation (i.e., increased levels of proinflammatory RAGE ligands) and circulating mediators of inflammation, [e.g., total leukocyte counts as well as the proportions of monocytes and neutrophils as examined human subjects[49].

2.2 Rationale

Mobile technology empowers clinicians to deliver targeted, in-the-moment, in-context behavioral interventions. They also permit remote delivery of counseling in a manner that is convenient for patients and has great potential for dissemination. Our group has developed an innovative weight loss intervention involving; (1) cloud-based self-monitoring of lifestyle behavior (diet and physical activity) followed by automated feedback, (2) theory-based behavioral counseling delivered via WebEx group sessions, and (3) standardized education to minimize calorie intake and enhance energy expenditure. Our preliminary data indicate that the intervention results in clinically significant weight losses in patients with type 2 diabetes (T2D) and chronic kidney disease.

Mechanistic evidence links postprandial glycemic response to weight gain. Recent data also show that postprandial glycemic response is highly individual, and that the composition and function of the intestinal microbiota are critical factors in glucose homeostasis. Dr. Segal has developed and validated a machine learning algorithm that integrates gut microbiota, dietary intake, physical activity, and various blood parameters to accurately predict postprandial glycemic response. He further demonstrated that this algorithm can be used to personalize dietary counseling and reduce glycemic response. In the current study, we will compare standardized and personalized counseling approaches on weight loss, weight maintenance, body composition, and metabolic adaptation.

This 2-phase, randomized clinical trial will be conducted in 210 overweight or obese pre-diabetic and early-stage diabetic individuals recruited from community-based settings. Phase 1 will include 6-months of active intervention. Phase 2 will consist of 6-months of maintenance and observation. Measurements will occur at screening, baseline, 3, and 6 months, and 12 months for a subgroup of participants for which data collection will end in October 2020. Participants will be randomized with equal allocation to 2 groups: (1) a standardized behavioral weight loss intervention with a one-size-fits-all regimen that includes counseling about restriction of calories and calories from fat, and physical activity, delivered using mHealth technology, (hereafter *mHealth*),

or (2) all of the elements of *mHealth*, plus personalized dietary recommendations to minimize glycemic response to meals (hereafter *personalized-mHealth*).

We will evaluate the impact of the interventions on Phase 1 weight losses, Phase 2 weight maintenance, and body composition and metabolic adaptation throughout. We will explore the mediating roles of glycemic variability (GV), and self-efficacy in all participants. In a subset of the first 58 participants (n=29 *mHealth* participants and n=29 *personalized-mHealth* participants) having a BMI≥35 kg/m2, we will examine the mediating effects of inflammation, and receptor for advanced glycation end products (RAGE/S100A8/A9) on outcomes at 6 and 12 months.

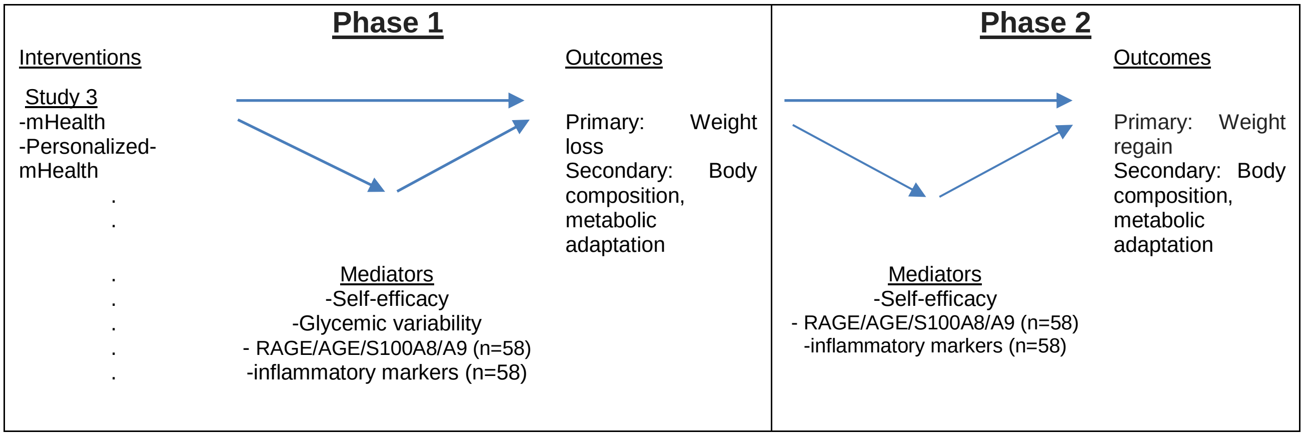

2.3 Potential Risks & Benefits

2.3.1 Known Potential Risks

- Risks of blood testing[**]. Blood testing will be performed for screening and baseline. Bruising, bleeding, and minor tenderness at the puncture site sometimes accompany blood tests. Occasionally, but rarely, infection and fainting may occur. In very rare cases, nerve damage may occur as the result of a blood test. By the end of the 12 months, a maximum total of 57.5 mL from the mHealth will be drawn (an additional 62 mL if in the subgroup of 58 people) if all tests are completed. By the end of the 12 months, a maximum total of 60.5 mL from Personalized mHealth will be drawn (an additional 62 mL if in the subgroup of 58 people) if all tests are completed. Our calculations indicate that the mHealth group (37.0 mL) and Personalized mHealth group (40.0 mL) will both be below the cut-off point for an 8-week window (see footnotes below for more detail).

Group	Time point	Total amount (mL)	Time period drawn
mHealth	Screening	6.5	0
Personalized mHealth	Screening	6.5	0
mHealth	Baseline	15.0	2-3 wks
Personalized mHealth	Baseline	18.0	2-3 wks

[**] In March 2020, all in-person measurement visits were halted due to COVID-19. All measurement visits after this point were conducted remotely, and blood testing was removed.

mHealth sub group 58	Baseline	37.0††	2-3 wks
Personalized mHealth sub group 58	Baseline	40.0**	2-3 wks
mHealth	3-month/ 6 month	18.0	3 mos + 2 wks/ 6 mos + 2wks
Personalized mHealth	3-month/ 6 month	18.0	3 mos + 2 wks/ 6 mos + 2wks
mHealth sub group 58	3-month/ 6 month	30.5	3 mos + 2 wks/ 6 mos + 2wks
Personalized mHealth sub group 58	3-month/ 6 month	33.5	3 mos + 2 wks/ 6 mos + 2wks
mHealth sub group 58	12-month	15.5	6 mos
Personalized mHealth sub group 58	12-month	15.5	6 mos

- Risks of finger stick testing. The last 3 cohorts (starting October 2020) will conduct finger stick testing to test HbA1c for eligibility. Subjects may have to prick their finger 2-3 times in order to get an accurate reading. Bruising, bleeding, pain, redness, and minor tenderness at the finger stick site sometimes accompany these tests. Occasionally, but rarely, infection may occur.
- The risk of fasting. Fasting for 8-12 hours could cause dizziness, headache, stomach discomfort, or fainting.
- CGM: Subjects may experience some mild or moderate symptoms associated with the Sensor application or the adhesive wipe (Skin tac) used to keep the Sensor in place. These include erythema, edema, rash, itching, bruising, pain, bleeding, and induration. Infection, inflammation, or bleeding at the sensor insertion site is possible risks of inserting sensor to the skin. It is rare that subjects will require treatment other than over-the-counter treatment. Most cases are caused by a reaction to the device adhesive. A microscopic piece of the sensor membrane may occasionally leave underneath the skin after the sensor is removed. This poses no health or safety risk and will dissolve on its own. The participant may experience discomfort when the CGM is removed. To avoid discomfort, participants will be shown how to remove the CGM.
- The risk of hypoglycemia (low blood sugar): Symptoms of hypoglycemia include sweating, jitteriness, and not feeling well. Occasionally, there is the possibility of fainting or seizures (convulsions). Because participants are not taking diabetes medications or are taking only metformin, it is highly unlikely that participants will experience hypoglycemia.
- The risk of hyperglycemia (high blood sugar): Hyperglycemia usually does not cause many obvious symptoms, but participants may become thirsty, or have higher levels of sugar in their urine. In severe cases of hyperglycemia, diabetic ketoacidosis (DKA) or coma may occur. Because the participants we have recruited early-stage diabetes and the body continues to produce enough insulin to maintain glucose homeostasis, it is highly unlikely that participants will experience hyperglycemia.
- The risk to privacy and confidentiality**:** A variety of measures are used which will reduce information security risk. First, all study staff will be trained in the NYULMC Research Practice Fundamentals, which

†† A maximum of 37.0 mL (6.5 + 30.5 [15.0 + 15.5]) will be drawn within an 8-week period (screening + baseline) in the mHealth sub group of 58 (BMI ≥35)

** A maximum of 40.0 mL (6.5 + 33.5[15.0 + 18.0) will be drawn within an 8-week period (screening + baseline) in the personalized mHealth sub group of 58 (BMI ≥35).

include training in issues of confidentiality. All study staff will be required to sign a confidentiality agreement. Data will be maintained in separate files for identified and de-identified data in locked file cabinets in a locked office. Access to these data will be restricted to the PI (Sevick), the medical co-Is (Dr. Michael Bergman), the project manager, and study staff responsible for gathering data and maintaining research files. Data will be entered into a centralized database maintained on a secure server. Data in these files will be linked to participants only through their ID number. All data collected will be used expressly for the purpose of the proposed study

2.3.2 Known Potential Benefits

At the conclusion of the study, we will share with participants those foods that resulted in the largest postprandial hyperglycemic excursions. Participants may benefit from the knowledge they gain regarding their personal glycemic response to foods. However, there are no guarantees that participants will benefit from participation in the study.

3 Objectives and Purpose

3.1 Primary Objective

To determine the impact of *mHealth* and *Personalized-mHealth* on weight loss at 6-months[‡‡].

3.2 Secondary Objectives

To determine the differences between *mHealth* and *personalized-mHealth* participants in terms of **2.2a** body composition, and **2.2b** metabolic adaptation at 6 months, and 12 months in a subgroup of participants for which data collection will end October 2020, and **2.2c** weight regain at 12 months in a subgroup of participants for which data collection will end October 2020.

To determine the mediating effect of **2.2.d** self-efficacy on the relationship between randomization assignment and weight loss/regain at 6 and 12 months. To compare the mediating effect of changes in **2.2e** GV (6 months only), **2.2f** RAGE/AGE/S100A8/A9 (6 and 12 months), and **2.2g** inflammatory markers (6 and 12 months) on body composition and metabolic adaptation within and between treatment groups.

4 Study Design and Endpoints

4.1 Description of Study Design

We will conduct a 2-phase, 2-group randomized clinical trial in overweight or obese patients recruited from the community. Patients must possess a smart phone or be willing to use a study loaner smart phone with data plan to record diet and physical activity, sleep and wake times, stressful events, and weekly weights. Phase 1 will involve an active intervention phase of 6 months duration, followed by another 6-month observation/maintenance phase. Participants will be randomized, with equal allocation to the *mHealth,* or the *personalized-mHealth* group. As discussed in more detail below, *mHealth* participants will receive 6 months of group behavioral counseling regarding physical activity and a one-size-fits-all, low-fat, calorie-restricted, diet. *Personalized-mHealth* participants will receive dietary counseling to minimize specific foods predicted by the PNP algorithm to result in a high glycemic response. In Phase 2, a subgroup of *mHealth* and *personalized-mHealth* participants will receive an additional 6 months of maintenance/observation, during which time they will be encourage to continue to self-monitor but no additional behavioral counseling. *mHealth* participants will receive immediate feedback on calories consumed and the extent to which they had been able to remain under

[‡‡]In March 2020, all in-person research visits were halted due to COVID-19. Data collection for the primary outcome was transitioned to remote collection via participants' home scale. This will be taken into account during the final analysis and is not anticipated to have a significant effect on the primary outcome.

This material is the property of the NYU School of Medicine and Langone Medical Center. Do not disclose or use except as authorized in writing by the study sponsor

their recommended ceiling. *Personalized-mHealth* participants will receive additional tailored feedback about predicted glycemic response.

Participants will use smart phones to connect to intervention applications. Participants will either use their own smart phone, or a study loaner smart phone with service plan. Participants will be trained in the use of the technology during a pre-intervention, one-on-one training session. To minimize participant burden of attending face-to-face group sessions, SCT-based counseling will be delivered with group sessions conducted via WebEx™ using their smart phone. WebEx™ is a communications application on the NYULMC Cisco Server, which is a highly secure, HIPAA-compliant, fully virtualized, behind-the-firewall conferencing program. WebEx™ allows users to sign-in securely, and join meetings from mobile devices without requiring VPN access to the corporate network. WebEx™ can accommodate a nearly unlimited number of participants, but we will limit the group size to 10.

Self-monitoring will be carried out using the PNP app, which will be downloaded onto the participants' smart phones (to allow use when at home and on-the-go). For the duration of the intervention, participants will be directed to record everything that they eat or drink, hunger at the time meals are consumed (via visual analog scale), physical activity, stress, medications, sleep time and wake time. The PNP app will be pre-programmed with a: (1) weight loss target (-7% body weight); (2) hypocaloric energy target (-500 kcal/day, based on basal energy expenditure measurements from indirect calorimetry and a physical activity factor of 1.4 (lightly active); (3) dietary fat target of 25% of kcal; (4) physical activity target of 30-minutes per day. The PNP app interface will allow participants to review their records in relation to targets in real-time. The flow of study activities by randomization group, as they are experienced by the participants, appears in Table 1 below.

CONFIDENTIAL
This material is the property of the NYU School of Medicine and Langone Medical Center. Do not disclose or use except as authorized in writing by the study sponsor

**Table 1. Flow of participant activities**		
**Timing**	**mHealth**	**Personalized-mHealth**
4-6 weeks pre-intervention	Obtain verbal consent and screen participants to determine eligibility	
3 weeks pre-intervention	**(1) Screening visit** (non fasting)<br>✓ Obtain signed informed consent via RedCap e-consent framework<br>✓ HbA1c (<8%)<br>✓ Serum creatinine (eGFR >60 ml/min)<br>✓ Height and weight (BMI 27-50 kg/m^2)<br>✓ Email questionnaires via Redcap or provide via paper/pencil (personal habits, medical history, sociodemographics, self-efficacy)<br>✓ Obtain availability for intervention sessions via Redcap survey<br>✓ Load Personalized Nutrition Program (PNP) app onto phone and train participant in its use<br>✓ Participant enters dietary intake for 1 week into PNP app<br>**For the last 3 cohorts, measurements will be done remotely. Hba1c will be tested via finger stick test, height and weight will be self-reported, and questionnaires will be done over WebEx or telephone.**	
2 weeks pre-intervention	**(2) Baseline measurement visit** (fasting)<br>✓ Staff reviews PNP entries with participant, exclude from further participation those who enter <2 meals/day, retrain as needed<br>✓ Resting energy expenditure<br>✓ Bioelectrical impedance analysis<br>✓ Waist, hip, and neck circumference<br>✓ Collect questionnaires (if completed via paper and pencil)<br>✓ Insert CGM<br>✓ Provide activity monitor(s)<br>UNBLIND RANDOMIZATION ASSIGNMENT to determine whether testing glycemic profiling labs<br>Blood testing (for all participants):<br>Fasting glucose & insulin, plasma metabolomics<br>In first 58 participants (29 per group) having BMI≥35 kg/m2:<br>RAGE/AGE/S100A8/A9 and inflammatory markers<br>✓ **In the last 3 cohorts, study measures will be conducted remotely. No blood testing, bioelectrical impedance analysis, or resting energy expenditure will occur.**	
		✓ Glycemic profiling labs (CBC)<br>✓ Provide stool sample kit with directions for collection
1 week pre-intervention	**(3) Baseline device visit** (non fasting)<br>✓ Collect CGMs<br>✓ Collect activity monitor<br>✓ Load WebEx app onto phone and train participant in joining WebEx teleconference calls via their smart phone<br>✓ 24 hour recall<br><br>**In the last 3 cohorts, no in-person baseline device visit will occur. All training will be done remotely at the screening or baseline visits, and**	

	**study materials will be mailed to and from particicpants.**	
		✓ Collect stool sample
Phase 1 Intervention	6-month active *mHealth* intervention<br>✓ One-on-one phone call session with study dietitian to review staying under recommended ceiling for calories and fat.	6-month active *personalized-mHealth* intervention<br>✓ One-on-one phone call session with study dietitian to review to discuss personalized meal and snack plans and the use of real-time feedback on predicted glycemic response.
3 months from start of intervention	**(4) 3 month measurement visit** (fasting)<br>✓ Blood testing Glucose, Insulin, and HbA1c<br>✓ Weight, waist, hip, and neck circumference<br>✓ Resting energy expenditure<br>✓ Bioelectrical impedance analysis<br>✓ Self-efficacy questionnaire<br>✓ 24 hour recall<br>✓ Insert CGM<br>✓ Provide activity monitor(s)<br>✓ Provide postage-paid return box for return of CGM and activity monitor to investigators<br>✓<br>✓ **In the last 3 cohorts, study measures will be conducted remotely. No blood testing, bioelectrical impedance analysis, or resting energy expenditure will occur.**<br>In first 58 participants (29 per group) having BMI≥35 kg/m2: RAGE/AGE/S100A8/A9 and inflammatory markers	
6 months from start of intervention	**(5) 6 month measurement visit** (fasting)<br>✓ Blood testing Glucose, Insulin, and HbA1c, plasma metabolomics<br>✓ Weight, waist, hip, and neck circumference<br>✓ Resting energy expenditure<br>✓ Bioelectrical impedance analysis<br>✓ Self-efficacy questionnaire<br>✓ 24 hour recall<br>✓ Insert CGM<br>✓ Provide activity monitor(s)<br>✓ Provide postage-paid return box for return of CGM and activity monitor to investigators<br>✓ In first 58 participants (29 per group) having BMI≥35 kg/m2: RAGE/AGE/S100A8/A9 and inflammatory markers<br>✓ **In the last 3 cohorts, study measures will be conducted remotely. No blood testing, bioelectrical impedance analysis, or resting energy expenditure will occur.**	
Phase 2 Maintenance (subgroup of participants; data collection ending October 2020)	✓ Participants continue to self-monitor diet and physical activity using their cell phone for the next 6 months and receive immediate feedback about targets but no further intervention contacts by dietitian. Participants will receive seasonal newsletters by mail until 12 months.	✓ Participants continue to self-monitor diet and physical activity using their cell phone for the next 6 months and receive immediate feedback about targets and predicted postprandial glycemic response, but no further intervention contacts by dietitian. Participants will receive seasonal newsletters

This material is the property of the NYU School of Medicine and Langone Medical Center. Do not disclose or use except as authorized in writing by the study sponsor

		by mail until 12 months.
12 months from start of intervention (subgroup of participants; data collection ending October 2020)	**(6) 12 month measurement visit** (fasting)<br>✓ Weight, waist, hip, and neck circumference<br>✓ Resting energy expenditure<br>✓ Bioelectrical impedance analysis<br>✓ Self-efficacy questionnaire<br>✓ End of program questionnaire<br>✓ 24 hour recall<br>✓ In first 58 participants (29 per group) having BMI≥35 kg/m2: RAGE/AGE/S100A8/A9 and inflammatory markers	

Following telephone screening, potential participants will attend a screening visit at the NYU NIH-CTSI-funded Clinical Research Center (CTSI-CRC) to verify eligibility. Study staff will measure height (cm) and weight (kg) before obtaining consent. If participants are below or above eligible BMI range (27-50 kg/m2), they will not be consented and will be dismissed with payment for visit.
Study staff will obtain consent before collecting blood for HbA1c and serum creatinine for eGFR.
Participants consented during the COVID-19 pandemic will be consented via Redcap e-consent framework as described in section 13.3.2. All participants will be provided with or emailed demographic questionnaires via Redcap to be filled out for the baseline measurement visit should they be eligible for the study based on their HbA1c and eGFR. Participants will also be asked to fill out a Redcap survey noting their availability for the WebEx intervention sessions (administered via email or in person on an iPad). The PNP app will be downloaded on all participants' smart phones, and participants will be trained to use the application. Participants will be told that their PNP app entries will be reviewed with study staff at baseline device visit. Participants will be excluded from participation if they have entered <2 meals/day during training observation period.

Before the baseline measurement visit, study staff will review PNP entries, and participants will be excluded from participation if they have entered <2 meals/day during training observation period. At the baseline measurement visit, eligible participants will return their questionnaires, as well as undergo additional bloodwork for fasting glucose and insulin and plasma metabolomics, and resting energy expenditure, anthropometrics. All participants will have an Abbott FreeStyle Libre Pro continuous glucose monitor (CGM, which captures interstitial glucose every 15 minutes) inserted into their arm. All participants will be provided with activity monitors to capture heart rate, continuous sleep and physical activity. After one week, participants remove the CGM sensor, place it into a sharps-proof container, and return it and the activity monitor to the investigators at the baseline device visit. If a participant's CGM falls off, they may be asked to join the next cohort. If a participant's CGM falls off more than one time, they may be excluded from participation.

In a subgroup of participants, we will measure temporal patterns of eating as well as physical activity and sleep. Participating in this subgroup is optional. If participants opt-in, we will ask them to wear an additional activity monitor for 8 days after baseline, 3 month, and 6 month visits in addition to the aforementioned measures. Participants will be required to charge the activity monitor each night. Study staff will contact participants daily to assure adherence to protocol.

Randomization will be unblinded to study staff, and PNP predictive algorithm profiling laboratory measurements (CBC), will be obtained from participants in the *personalized mHealth* group. Participants in group 2 will also be given a stool kit and directed to collect their stool sample preceding the baseline visit. Participants with BMI ≥35 kg/m^2 will also provide blood work for inflammatory markers, adipokines, WBCS, and platelets.

At the baseline device visit, all participants will have their CGMs and activity monitors collected. All participants will be asked to provide a 24 hour food recall, which will then be entered into their apps. Participants in group 2 will have their stool kits collected. At this visit, participants will also have the WebEx app downloaded on their phone or the study loaner phone, and will be trained on use of how to join intervention meetings.

- ✓ **In the last 3 cohorts, all study measures will be conducted remotely. No blood testing, bioelectrical impedance analysis, or resting energy expenditure will occur. Training done at baseline device visit will occur during screening and baseline visits. Prior to screening visit, participants will receive a point-of-care HbA1c testing kit (A1cNow Systems, PTS Diagnostics,**

Whitestown, IN) and will conduct a finger stick to test HbA1c for eligibility. Before baseline, 3, and 6 month visits, participants will receive a Renpho Bluetooth scale, a CGM, an activity monitor, a measuring tape, and a self-addressed prepaid mailing envelope for return. They will be trained on how to use these instruments and observed by study staff via Webex Participants in the personalized group will also receive a stool sample kit. No maintenance phase or 12 month visit will take place for these cohorts.

Stool samples for the personalized mHealth group are express shipped to the Weizmann Institute (see Section 6.2.4) for microbiome analysis (see section 6.2.4). Genomic DNA extraction purification will be carried out using PowerMag Soil DNA isolation kit (MoBio) optimized for Tecan automated platform. For shotgun sequencing, 100 ng of purified DNA will be sheared with a Covaris E220X sonicator. Illumina compatible libraries will be prepared as described[71]. For 16S rRNA sequencing, PCR amplification of the V3/4 region will be performed using the 515F/806R 16S. Computational analysis: Relative Abundance from 16s rRNA and metagenomics sequencing reads will be performed using USearch8.0 [72] and MetaPhiAn2 respectively according to default setting and peak-to-trough ratio (PTRs) will be extracted [73]. For all analyses samples with >10K reads of 16S rRNA and >10M metagenomics reads will be considered. The metagenomic reads containing Illumina adapters will be filtered for low quality reads and trimmed for low quality read edges. We will detect host DNA by mapping with GEM to the Human genome with inclusive parameters, and remove low quality reads. Furthermore, key microbiota-based features that are associated with improved glucose tolerance and glycemic responses to food will be captured. These will include: (a) **Bacterial abundances,** obtained by mapping the sequencing reads to a large reference bacterial genome database and counting the number of reads mapping to each bacteria. (b) **Bacterial diversity,** derived by computing several measures of the diversity of bacteria in a metagenome sample (e.g., Shannon entropy of the relative bacterial abundances), as sample diversity was shown to be associated with overall adiposity and insulin resistance[33]. (c) **Bacterial growth rates,** using a novel computational method that we developed based on analysis of the ratio between the peak and trough of read coverage across the genome. (d) **Gene abundances,** through mapping of the reads to a reference bacterial gene dataset consisting of over 3 million distinct genes[35]. (e) **Biological pathway abundances,** providing information at the functional level of the microbiota using the KEGG [74] data of biological pathways and the above gene abundances. (f) **SNPs and structural variations,** derived by comparison to reference bacterial genomes and generating for every sample a feature vector of SNPs, insertions, and deletions. We will extract these features from every microbiome sample and score it by the presence and absence of features that we identified as being associated with improved glucose tolerance and glycemic responses. The Weizmann Institute will combine gut microbiome profiles with other profiling data to develop an individualized predictive model of glycemic response. As described in section 3.b.3. Decision Tree machine learning will be used to generate each participant's algorithm, optimized in an iterative fashion using a leave-one-out cross validation scheme, with meal responses predicted from the data derived from Segal's preliminary studies. These data will be used to provide feedback on predicted glycemic response, which will appear on the PNP app at the time the participant enters a planned meal or snack. The algorithm will be used to generate meal plans and real-time feedback about planned meals as discussed below.

Participants in both groups will be provided with sample meals for their respective assigned diets. As noted above, meals that do not meet the participant's requirements will be filtered out. For each meal in the database, we will use the PNP algorithm to compute the predicted glycemic response at 2 hours ($iAUC_{pred}$), and rank order the meals according to the $iAUC_{pred}$. Rank-ordered meals will be shared with the participant during the one-on-one pre-intervention phone call with the study dietitian. At this time, a dietitian will discuss the participant's predicted glycemic response to the different meals and encourage them to consume meals that have an $iAUC_{pred}$ that is 1 SD below their average iAUC. In group sessions, participants will share PNP app feedback that they have received, their observations about foods that appear to be problematic for them, and changes they are making to their diet to remain under their target iAUC. While not a specific aim of the study, group sessions will be recorded using WebEx, and will be available for subsequent qualitative analysis to characterize the nature of dietary decisions made. Glycemic response feedback from the PNP app will be available only for those randomized to the *personalized-mHealth* arm.

Phase 1 active intervention begins following the device visit. Randomization arm interventions are described below. Participants will come back for measurements at 3 months and 6 months (and 12 months visits will occur for a subgroup of participants for which data collection ends October 2020) following the start of the intervention. Activities for each visit are listed in Table 2. During the period of active intervention, study staff will clarify PNP entries with participants by phone when possible; participants may also consent to have

their PNP diet and physical activity entries clarified by text. Texts may also be sent by the study team to remind participants about appointment times and locations. All texts generated by the study team will be sent to participants using a secure phone obtained from MCIT. After the 6-month measurement visit, Phase 2 maintenance and observation begins. During this time, participants in the *mHealth* group continue to self-monitor diet and physical activity using their cell phone and receive immediate feedback about targets, but no further intervention contacts by dietitian. Participants in the *personalized mHealth* group continue to self-monitor diet and physical activity using their cell phone, and receive immediate feedback about targets and predicted postprandial glycemic response, but no further intervention contacts by dietitian. Study staff will mail out monthly nutrition-based newsletters in order to retain interest.

Independent Variable: Randomization arm

Table 2: Intervention content

	**Education Materials (Video)**	**Social Cognitive Theory (Coaching)**
1	Overview of obesity risks and benefits of weight loss.	Goals for Life
2	Self-monitoring of diet and physical activity - making sense of the numbers.	Where am I? Establishing the relevance of behavior change.
3	Being a Calorie Detective. Portion control and empty calories.	Setting goals
4	Introducing physical activity into your life. Finding time for fitness. Exercise safety.	Self-Reward. Turning goals into habits.
5	Being a fat detective. Healthy and unhealthy fats, the contribution of fat to total calorie intake.	Social support. Developing and working your social support network
6	Building duration and intensity of aerobic exercise	Problem solving: Barriers and setbacks. Introduction to the problem solving model.
7	Changing seasons, special occasions, life events, and eating at restaurants	Problem solving: Behavioral triggers and stimulus-control
8	The role of sleep and stress in weight gain and loss.	Problem solving: Stress management.
9	Adding color and fiber to your diet.	Problem solving: Emotional eating
10	The role of breakfast and meal frequency in weight loss success.	Problem solving: Eliminating negative self-talk
11	Snacking and sugar-sweetened beverages. Empty calories	Problem solving: Food cravings, addictions, and habitual over-eating
12	Building muscles with strength training.	Problem solving: Anticipating high-risk situations
13	Weight loss plateaus.	Problem solving: Lapse and Relapse
14	Putting it all together; review of lifestyle recommendations	Problem solving: Coping with lapses and setting new goals

mHealth. Participants randomized to *mHealth* will be enrolled in an intensive technology-supported behavioral intervention program targeting a 7% weight loss. The behavioral goals of this program are adapted

from the Diabetes Prevention Program (DPP) trial, and include low-fat diet (<25% fat), moderate-intensity physical activity (150 mins/week), and self-monitoring (described below) [4].
The behavioral component of the *mHealth* intervention will be based on SCT[68, 69], which focuses on the role played by self-referent thought in the maintenance of behavior change. Self- efficacy (e.g., the participant's confidence in their ability to engage in healthier behavior) is derived from four major sources of information: mastery experiences, social modeling, verbal persuasion, and physiological states. **Mastery experiences** will include emphasizing past successes; setting incremental, easily achievable goals; identifying modifiable barriers to healthy behavior; receiving positive feedback on goal achievement; and practicing problem solving skills around barriers to adherence. With **social modeling**, mastery will be enhanced when participants share their successes and help each other problem-solve around barriers they encounter. **Verbal persuasion** will include emphasizing the participant's previous successes to demonstrate their capability (e.g., "As a result of your effort, you lost a pound last week. You can do it again.") We will assist participants in recognizing **physiologic benefits** they experience as a result of dietary changes and increased physical activity (e.g. looser clothing, more energy, better sleep, better glucose or BP control). Content to be delivered is outlined in Table 2.

Participants will be provided with examples of meals and snacks from a meal database developed by our group. Briefly, the menu database was designed to include mixed meals and snacks of varying nutrient composition by combining a variety of commonly consumed foods and beverages from different food groups. To achieve this, we first selected representative foods for a variety of food subgroups within each food group. Study dietitians will review the finalized list of example meals and snacks, and remove any of the suggested meals and snacks that contain non-food group items that contradict the general healthy eating recommendations of the study (e.g., soda). Items from different food groups (or non-food group items) were then combined to make meals with 3-5 food groups and snacks with 2-3 food groups, taking into consideration the food combinations that are common in the Western diet. For example, breakfast cereals are rarely consumed alone, so these meals all had either low- or full-fat milk, or milk alternatives such as soymilk with them. Other non-food group items were added to meals and snacks, as necessary (e.g., dressings for salads, butter for baked potatoes). The list of example meals and snacks will be tailored to the participant. As a first step, meals and snacks that do not meet the participant's personal tastes and requirements will be filtered-out (e.g. vegetarian, religious preferences, intolerances/allergies). Meals and snacks will be rank-ordered in terms of fat calories and classified as either compliant (≤25% of kcal from fat), or non-compliant (>25% of kcal from fat). Meal plans will be shared with the participant by mail, and a study dietitian will call to review the plans with the participants in each group.

In Phase 1, the schedule of contacts is consistent with Medicare provisions for 6 months of intensive behavioral weight loss counseling. If the intervention is found to be efficacious, such a schedule would support downstream implementation[70]. During the first month participants attend weekly *WebEx™* group sessions, which are reduced in frequency to biweekly in months 2-6. During this intensive phase, intervention sessions focus on new lifestyle behavior change. Participants who miss a WebEx™ session will be sent an email containing a link to a brief video summarizing key points from the session. Videos are linked to a BrainShark account that allows us to document the duration of exposure to content independent of intervention sessions. During Phase 1, participants also will receive feedback emails from staff making note of the extent to which participants are self-monitoring, physically active, and staying under their recommended ceiling for calories and fat. In Phase 2 participants will enter a 6-month observation period, during which time they will be encouraged to continue self-monitoring their diet and physical activity with the PNP app. If a participant does not have an email address, they will be provided with an MCIT generated NYU study email address.

Personalized-mHealth. Participants randomized to the *Personalized-mHealth* arm will receive the same education sessions and technology-supported behavioral intervention program as the *mHealth* arm, however, in place of a one-on-one phone call to review their dietary fat ceiling, participants will receive a phone call from the study dietitian to discuss personalized meal and snack plans and the use of real-time feedback on predicted glycemic response.

4.2 Study Endpoints

Unless otherwise specified below, all measurements will occur at baseline, 3, 6, and 12 months.

CONFIDENTIAL
This material is the property of the NYU School of Medicine and Langone Medical Center. Do not disclose or use except as authorized in writing by the study sponsor
Behavioral Intervention Template Version: 5 MAY 2017

4.2.1 Primary Study Endpoints

The primary endpoint will be the relative weight change as a percentage of body weight at baseline (for weight loss at 6-months) and 6-months (for weight loss maintenance at 12-months in a subgroup of participants for which data collection will end October 2020) using a calibrated scale, with the participant directed to empty pockets and remove shoes and outerwear[6].

4.2.2 Secondary Study Endpoints

The secondary endpoints will include changes in body composition and metabolic adaptation. Specifically, we will examine the absolute and relative changes in fat and lean body mass based on bioelectrical impedance analysis (BIA; Omron, Hoofddorp) from baseline to 6-months (Phase 1), and 6- to 12-months in a subgroup of participants for which data collection will end October 2020 (Phase 2), and baseline to 12-months in a subgroup of participants for which data collection will end October 2020 (Phases 1 and 2). Metabolic adaptation will be defined as the change in resting metabolic rate overall, and in relation to body weight and lean body mass from baseline to 6-months (Phase 1), 6- to 12-months in a subgroup of participants for which data collection will end October 2020 (Phase 2), and baseline to 12-months in a subgroup of participants for which data collection will end October 2020 (Phases 1 and 2). Resting metabolic rate will be estimated using indirect calorimetry with the participant in a fasting state (12 hours).

4.2.3 Exploratory Endpoints

Mediator: Self-efficacy. We will measure self-efficacy pertaining to dietary weight loss, dietary sodium restriction, and physical activity using 3 separate measures. *Self-efficacy for weight loss* will be assessed using the well-validated Weight Efficacy Lifestyle Questionnaire (WEL). The WEL includes 20 items with which respondents rate, using a 10-point Visual Numeric Scale (VNS) ranging from 0 (not confident) to 9 (very confident), their confidence to resist eating under various circumstances such as negative emotions, availability, social pressure, physical discomfort, and positive activities[75]. For analyses pertaining to the mediating effect of self-efficacy on the relationship between weight loss and randomization group, an overall score and subscale scores will be computed by summing relevant WEL items.

Mediator: Glycemic variability (GV). GV will be evaluated at baseline, 3, and 6 months only. GV will be obtained from CGM tracings collected with the Abbott FreeStyle Libre Pro, which will capture glucose readings every 15 minutes for up to two weeks at each measurement time point. Participants will be blinded to glycemia tracings. GV will be evaluated primarily using the mean amplitude of glycemic excursion (MAGE), which generates a value of variation around the mean by summating the absolute rises or falls encountered in a day, ignoring excursions of less than 1 SD.[76] To insure interpretability with respect to the literature on GV, we also will obtain other measures of GV, including: (1) standard deviation, (2) Continuous Overall Net Glycemic Action (CONGA)[77], (3) Mean area under the curve (AUC) of the blood glucose levels following meals; (4) Number of events and total time during the week in which glucose levels were out of range ($\leq$70 and $\geq$180mg/dl); and seriously out or range ($\leq$50 and $\geq$300mg/dl). All values will be calculated using EasyGV 8.6 software[78].

Mediators: Circulating Mediators of Inflammation. In the first 58 participants randomized to the study (29 in each group) having BMI ≥35 kg/m^2, at each measurement time point we will examine activation of the RAGE/AGE/S100A8/A9 pathway using measurements of sRAGE, AGE level by ELISA as developed by Dr. Schmidt[79 80], levels of S100A8/A9 by ELISA, circulating TNF-alpha, IL1-beta, IL4, IL10, and IL-17.

Mediators: Adipokines. We will measure leptin and high molecular weight adiponectin each measurement time point in the subsample of participants having BMI ≥35 kg/m^2.

Mediators: WBC Collection and Platelet Analysis. In the subsample of participants having BMI ≥35 kg/m^2, we will characterize the circulating WBC populations by clinical tests (complete blood count), flow cytometric analysis for neutrophil and monocyte subpopulations and selected leukocyte transcript expression of targets along the RAGE/AGE/S100A8/A9 pathway.

Covariates: Health and sociodemographics. We will collect baseline information on health

[6]In March 2020, all in-person research visits were halted due to COVID-19. Data collection for the primary outcome was transitioned to remote collection via participants' home scale. This will be taken into account during the final analysis and is not anticipated to have a significant effect on the primary outcome.

CONFIDENTIAL
This material is the property of the NYU School of Medicine and Langone Medical Center. Do not disclose or use except as authorized in writing by the study sponsor

characteristics including age, race, gender, comorbid conditions, living arrangement, education, employment, and income. At each measurement time point we will collect information on medication regimen, identify health events and treatments (e.g., use of antibiotics, diabetes medications).

Covariates: Habits and history that could influence gut microbiome. These data would include country of origin for self, parents, and grandparents; hunger experienced throughout the day, morning, midday, and evening; sleep quality; smoking; frequency, timing and quality of bowel movements; antibiotic use; weight loss history; use of artificial sweeteners; birth and breastfeeding history; and menstrual cycle.

5 Study Enrollment and Withdrawal

5.1 Inclusion Criteria

In order to be eligible to participate in this study, an individual must meet all of the following criteria:

1. healthy overweight or obese, pre-diabetic or early-stage T2D individuals
    a. Prediabetes is defined as a diagnosis code of pre-diabetes/abnormal glucose OR an HbA1C of 5.7-6.4%.
    b. Early-stage T2D is defined as individuals who have a diagnosis code of T2D OR HbA1c 6.5-8% (to exclude those for whom hyperglycemic exposure is driven by β-cell failure rather than dietary behaviors)[7] [66].
2. between the ages of 18 and 80 years old.
3. must also possess a smart phone or be willing to use a study loaner smart phone.

We will limit our sample to those with a BMI 27-50 kg/m^2. We will include subjects with prediabetes, and T2D treated with only Metformin.

Screening and enrollment will be performed under the same informed consent. Enrolled participants will not be asked to sign an additional informed consent after the screening process. An HbA1c test will be completed during the screening visit (visit 1).

5.2 Exclusion Criteria

An individual who meets any of the following criteria will be excluded from participation in this study:

1. unable or unwilling to provide informed consent
2. unable to participate meaningfully in an intervention that involves self-monitoring using software available in English (e.g., due to uncorrected sight impairment, illiterate, non-English-speaking, dementia)
3. unwilling to accept randomization assignment
4. women who are pregnant, or plan to become pregnant in the next 13 months, or who become pregnant during the study
5. institutionalized (e.g., in a nursing home or personal care facility, or those who are incarcerated and have limited control over diet)
6. has previously had bariatric surgery
7. unwilling to delay bariatric surgery for the next 12 months
8. unable to walk without a walker or cane for 2 city blocks

---

[7] In a landmark study of T2D patients treated with diet, with or without oral antihyperglycemic drugs, Monnier et al found that when patients had an HbA1c<7.3%, postprandial hyperglycemia played a greater role in overall glycemic exposure than fasting hyperglycemia; whereas the contribution of fasting hyperglycemia increased gradually with worsening glycemic control.[57] In a subsequent study, these findings were confirmed with continuous glucose monitoring (CGM). The investigators showed that glycemic control deteriorated with duration of T2D in a 3-step process: starting with loss of postprandial glucose control, followed by loss of glycemic control during the pre- and post-breakfast periods, and finally sustained hyperglycemia during the night followed by fasting hyperglycemia (step 3, or later disease).[58] Consequently we will focus our recruitment efforts on those with recently diagnosed T2D, not receiving insulin or GLP-1 agonist, with an HbA1c<8%.

CONFIDENTIAL
This material is the property of the NYU School of Medicine and Langone Medical Center. Do not disclose or use except as authorized in writing by the study sponsor

9. unwilling to receive medical clearance for physical activity from physician (if diagnosed with heart disease or has suffered a stroke)
10. diagnosed with a chronically active inflammatory or neoplastic disease in the past 3 years
11. diagnosed with any chronic disease that is known to affect energy/glucose metabolism or require special dietary management as determined by study investigators
12. diagnosed with a chronic gastrointestinal disorder (e.g. inflammatory bowel disease or celiac disease)
13. diagnosed with active infection requiring antibiotics or oral antifungals in the last 3 months or who develop an active infection requiring antibiotics or oral antifungals during the study
14. taking medications containing aspirin and are unwilling or unable to discontinue its use during the study (aspirin affects accuracy of the continuous glucose monitoring [CGM] device)
15. taking chronic immunosuppressive medications or used them in the 3 months prior to participation, or during the study
16. managing glycemia with insulin, GLP-I agonists (exenatide, liraglutide, lixisenatide, albiglutide, dulaglutide), insulin secretagogues (Glimepiride, Glipizide, Glyburide, Repaglinide, Nateglinide), or SGLT2 inhibitors (canagliflozin, dapagliflozin, empagliflozin, empagliflozin/metformin, dapagliflozin/metformin)
17. prescribed medications expected to result in weight loss such as Orlistat, Naltrexone, Bupropion, Lorcaserin, Phentermine, Topiramate, or Liraglutide, and who are unwilling to delay treatment with these medications for the next 12 months
18. taking other medications that could interfere with weight loss including steroids and anti-psychotics
19. +/- 5% body weight loss or gain within one month of screening
20. a eGFR <60 mL/min/1.73m^2
21. younger than 18 or older than 80 years old.

5.3 Vulnerable Subjects

No vulnerable populations are included in this study.

5.4 Strategies for Recruitment and Retention

We will recruit participants from the community and clinical settings using the following 7 strategies: (1) *News advertisements*. Advertisements will be placed in three news websites covering the local New York City area: Village Voice, Metro, and amNewYork. Advertisements for research studies also will be posted in hard copy versions of Metro, and amNewYork which are distributed at subway entrances throughout the city. (2) *Mass Transit Authority (bus and subway) advertisements*. Interior ads will be placed in busses and subway stations in Manhattan, the Bronx, Queens, Brooklyn, Staten Island, and Long Island. (3) *Direct mailings*. Direct mail lists generated from responses to marketing surveys targeting overweight individuals will be purchased from local marketing companies. (4) *Direct-to-persona marketing via social media*. Enov8 Mobile works with investigators to develop hyper-targeted recruitment campaigns via data mining of internet search terms and posts to Facebook, Twitter, blogs and forums. (5)*Local neighborhood business and organizations*. We will recruit via established partnerships with local churches, barber and beauty shops, and social service agencies. (6) *Local medical and surgical weight management programs*. We will have access to patients seeking weight management services at the NYU Langone Health affiliates, Weight Management Programs, and the Bellevue Hospital Comprehensive Obesity Center. (7) *Study Website*: A study website will be used as a recruitment tool sent as a link with recruitment messages providing general information on the study and answers to frequently-asked questions. Once participants are enrolled in the study, the study website will contain password-protected calendars and educational materials; no direct communications will be made with participants through the website and no PHI will be used or available within the study website. (8) *Study recruitment video:* A brief study recruitment video outlining information about the study will be featured on the study website and sent as a link with recruitment messages. Interested participants will contact study staff directly with contact information provided on approved recruitment materials. Study staff will describe the study and if the patient expresses an interest, conduct preliminary screening by telephone. Verbal consent is obtained by study staff prior to eligibility screening. Participants who have been diagnosed with heart disease or who have suffered a stroke in the last six months will need written medical clearance in order to participate in the study. Written informed consent will be obtained with subsequent face-to-face measurement visit to the CTSI for eligible participants. Participants

CONFIDENTIAL
This material is the property of the NYU School of Medicine and Langone Medical Center. Do not disclose or use except as authorized in writing by the study sponsor

consented during the COVID-19 pandemic will be consented via Redcap e-consent framework as described in section 13.3.2.

We will target recruitment to generate a sample that is representative of overweight or obese individuals living in New York. According to the 2012 Behavioral Risk Factor Surveillance Survey, the gender and race/ethnic distributions were as follows: 51.4% male, 34.2% non-Hispanic Black, 27.3% Hispanic, 25.2% non-Hispanic White, and 13.3% Other.[67] Special effort will be made to recruit a sample with a race and gender distribution consistent with New York City's Department of Health rates provided above, with 20-25% of individuals of Latino ethnicity. We have a history of successfully recruiting minorities to our studies. No individual will be excluded from the study on the basis of race or gender alone. It is possible that the study will have sufficient power to detect differences in intervention effect by gender and race. To our knowledge no research has been published showing a differential effect of interventions such as ours by race or gender.

5.4.1 Use of DataCore/Epic Information for Recruitment Purposes

We will use EPIC to identify potentially eligible patients seen in NYUMLC Faculty Group Practices and NYUMLC affiliates, based on DRG codes indicating presence of prediabetes and diabetes. We will use one of two methods described below to recruit eligible participants. The method choice for enrolling participants will be contingent on the treating physician's stated capacity for the quantity of their patients that they can review and recommend for study participation, and whether participants self-refer to the study:

Method 1: Clinical-Initiated contact with TP review prior to pre-screening

We will develop a roster of potentially eligible patients for each treating physician in the practice. Physicians will be asked to review the roster and indicate, for each patient, whether an intervention targeting weight loss is appropriate. Lists of patients deemed appropriate (i.e., "yes") by the physician will be generated. Following written permission from NYULMC treating physicians to recruit their patients to the study, patients will be approached and screened using the following process: (a) A letter signed by the treating physician and Dr. Sevick will be sent to the treating physician's patients notifying them of the study, and informing that an NYULMC clinical staff person will contact them to explore their willingness to consider participation, or that their patients can call directly to the study staff to ask about the study. (b) An NYULMC clinical staff person will contact these patients to briefly describe the study and request their permission (yes/no) for study staff to contact them directly by telephone. (c) Study staff will call patients who agree to be contacted, describe the study and if the patient expresses an interest, conduct preliminary screening by telephone. Verbal consent is obtained by study staff prior to eligibility screening. (d) Written informed consent will be obtained with subsequent face-to-face measurement visit to the CTSI for eligible participants. Participants consented during the COVID-19 pandemic will be consented via Redcap e-consent framework as described in section 13.3.2.

Method 2: Clinic-initiated contact with TP review post pre-screening

We will develop a roster of potentially eligible patients for each treating physician in the practice. Following written permission from NYULMC treating physicians to recruit their patients to the study, patients will be approached and screened using the following process: (a) A letter signed by the treating physician and Dr. Sevick will be sent to the treating physician's patients notifying them of the study, and informing that an NYULMC clinical staff person will contact them to explore their willingness to consider participation, or that their patients can call directly to the study staff to ask about the study. (b) An NYULMC clinical staff person will contact these patients to briefly describe the study and request their permission (yes/no) for study staff to contact them directly by telephone. (c) Study staff will call patients who agree to be contacted, describe the study and, if the patient expresses an interest, conduct preliminary screening by telephone. Verbal consent is obtained by the study staff prior for eligibility screening. (d) These participants will be told that the study staff needs to verify suitability for this study with their treating physician and will be getting another call with the TP's decision. (e) Physicians will be asked to review the roster of patients eligible after preliminary screening by telephone to indicate, for each patient, whether an intervention targeting weight loss is appropriate. (f) Those patients deemed appropriate (i.e., "yes") would be scheduled for subsequent face-to-face visit to the CTSI to obtain written informed consent, and subsequently, measurement for eligible participants.

Participants consented during the COVID-19 pandemic will be consented via Redcap e-consent framework as described in section 13.3.2.

Method 3: Research staff-initiated contact with TP review prior and post pre-screening

We will develop a roster of potentially eligible patients for each treating physician in the practice. Physicians will be asked to review the roster either prior (method 1) or post (method 2) pre-screening. Following written permission from NYULMC FGP physicians to recruit their patients to the study, patients will be approached and screened suing the following process: (a) A letter signed by the treating physician and Dr. Sevick will be sent to the treating physician's patients notifying them of the study, and informing that an NYULMC research staff person will contact them to describe the study, or that their patients can call directly to the study staff to ask about the study. These patients will also be informed of an alternative for opting out of research studies. (b) Research study staff (instead of clinic staff) will call patients who agree to be contacted, describe the study and, if the patient expresses an interest, conduct preliminary screening by telephone. Verbal consent is obtained by study staff prior to eligibility screening.

Thereafter, the steps for Method 3 follow either Method 1 or 2 depending whether treating physicians decide to screen their patient list for eligibility prior or after research staff calls their patients.

The PIs, research coordinators and research dietitians will all have access to the EPIC search results. The data points and PHI that will be used for the search will be: T2D diagnoses codes, HbA1C lab results, DOB. Frequency of EPIC queries will depend on recruitment success. We anticipate running EPIC queries every 6 months until target participant numbers are met.

Method 4: Participant self-referral through EPIC electronic health record MyChart alerts

We will provide the NYU Langone Health Epic Research Integration team with lists of potentially eligible patients provided to us by NYU Langone Health DataCore services using the method through a prior IRB-approved waiver of authorization. The Epic team will send these patients an alert notification to inform patients to check MyChart electronic record for a new message.

We will provide an NYU Langone Health Epic Research Integration team with an IRB-approved patient-facing message to be posted in the Epic electronic health record patient portal (MyChart) with a brief study description with information for patients to contact study staff for any questions or to express their interest to participate. At this point, study recruitment proceeds as per protocol: (a) study staff describe the study and, if the patient expresses an interest, conduct preliminary screening by telephone. Verbal consent is obtained by study staff prior to eligibility screening. (b) Written informed consent will be obtained with subsequent face-to-face measurement visit to the CTSI for eligible participants.

Participants consented during the COVID-19 pandemic will be consented via Redcap e-consent framework as described in section 13.3.2.

If a participant requests information regarding opting out of further recruitment for all research, participants will be directed to contact personal.diet@nyumc.org or 646-501-2606.

Method 5: Participant self-referral through send [SAFE] email messages

Given that many potential participants on the DataCore list are not MyChart active and/or have not opened their MyChart messages, potential participants will be sent the same IRB-approved recruitment message to their personal emails via send [SAFE], a NYU Health secure messaging server.

5.5 Duration of Study Participation

The duration of the study for each participant will be between a minimum of 12 months for the intervention and an additional 8-12 weeks for pre-randomization screening, and baseline measurements. All in all, a total duration of 14-16 months.

This material is the property of the NYU School of Medicine and Langone Medical Center. Do not disclose or use except as authorized in writing by the study sponsor

5.6 Total Number of Participants and Sites

Recruitment will end when approximately 210 participants are randomized. We will recruit approximately 12 cohorts of 15-20 participants each for the sample size of 210 participants. A new cohort will be randomized approximately every 8 weeks during a 34-month recruitment period.

5.7 Participant Withdrawal or Termination

5.7.1 Reasons for Withdrawal or Termination

Participants are free to withdraw from participation in the study at any time upon request. An investigator may terminate participation in the study if:

- Failure to respectfully participate in the intervention requirements
- Any clinical adverse event (AE), laboratory abnormality, or other medical condition or situation occurs such that continued participation in the study would not be in the best interest of the participant
- The participant meets an exclusion criterion (either newly developed or not previously recognized) that precludes further study participation.

5.7.2 Handling of Participant Withdrawals or Termination

In the event that involuntary withdrawal is required, participants will be referred back to their treating physician of record for evaluation and management. Data collection will cease at the time of withdrawal. Data up until the point of withdrawal will be used in the analysis.

AEs, *serious adverse events (SAEs), and unanticipated problems (Ups)* will be recorded, and the participant's treating physician will be notified. *We do not anticipate the safety of participants to be compromised upon voluntary termination. Replacements are not allowed In the event of participant withdraw from the intervention.*

5.8 Premature Termination or Suspension of Study

This study may be temporarily suspended or prematurely terminated if there is sufficient reasonable cause. Written notification, documenting the reason for study suspension or termination, will be provided by the suspending or terminating party to Sevick (PI) and the American Heart Association. If the study is prematurely terminated or suspended, the PI will promptly inform the IRB and will provide the reason(s) for the termination or suspension.

Circumstances that may warrant termination or suspension include, but are not limited to:

- Determination of unexpected, significant, or unacceptable risk to participants
- Demonstration of efficacy that would warrant stopping
- Insufficient compliance to protocol requirements
- Data that are not sufficiently complete and/or evaluable
- Determination of futility

Study may resume once concerns about safety, protocol compliance, data quality are addressed and satisfy the sponsor and/or IRB.

6 Behavioral/Social Intervention

6.1 Study Behavioral or Social Intervention(s) Description

The behavioral component of the mHealth and Personalized mHealth intervention will be based on SCT. Detail of this intervention component has been discussed in section 4.1 of the study design.

CONFIDENTIAL
This material is the property of the NYU School of Medicine and Langone Medical Center. Do not disclose or use except as authorized in writing by the study sponsor

6.1.1 Administration of Intervention

Interventions will be delivered via one-on-one phone calls and WebEx group sessions. Missed WebEx sessions will be stored for viewing later. Study dietitians will deliver phone calls and WebEx sessions.

Participants will sign Audio Visual consent, and will be informed that *WebEx* communications are recorded. Participants consented during the COVID-19 pandemic will be consented via Redcap e-consent framework as described in section 13.3.2.

They will be told that under certain circumstances, such as a court subpoena or communications suggesting that participants or others known to the participant are at risk of harm, these records may be released to others. Recorded WebEx sessions will be saved by the study interventionist in password-protected files on the NYULMC server until study completion.

6.1.2 Procedures for Training Interventionists and Monitoring Intervention Fidelity

The staff interventionist is a credentialed Registered Dietitian with training in social cognitive behavioral theory. The interventionist will be supervised by the PI and research dietitians. Intervention videos will be viewed by the PI and study team to determine fidelity to the intervention material. The interventionist will also train study staff to monitor participant use of the PNP app.

6.1.3 Assessment of Subject Compliance with Study Intervention

Not applicable

7 Study Procedures and Schedule

7.1 Study Procedures/Evaluations

7.1.1 Study Specific Procedures

- Medical history*:* Demographics, emergency contact information, background information (i.e., gender, DOB), income and General Health information will be collected by questionnaire (this will be send home to participants during screening visit and will be returned during the baseline measurement visit)

- Medication history: medication history will be completed during screening or prior to screening visit. A complete medication history (current and previous), that includes over-the-counter and prescription medications will be collected to assess for eligibility

- Physical examination: Height (centimeters) and weight (kilograms) will be collected to assess BMI at screening. Weight will be assessed again at 3-, and 6-months, and 12-months in a subgroup of participants for which data collection will end October 2020.

- Biological specimen collection and laboratory evaluations*: See Section 7.2 Laboratory*

- mHealth and Personalized mHealth counseling: Participants will use smart phones to connect to intervention applications. Participants will either use their own smart phone, or a study loaner smart phone with service plan. Participants will be trained in the use of the technology during a pre-intervention, one-on-one training session. To minimize participant burden of attending face-to-face group sessions, SCT-based counseling will be delivered with group sessions conducted via WebEx™ using their smart phone. WebEx™ is a communications application on the NYULMC Cisco Server, which is a highly secure, HIPAA-compliant, fully virtualized, behind-the-firewall conferencing program.

WebEx™ allows users to sign-in securely, and join meetings from mobile devices without requiring VPN access to the corporate network. WebEx™ can accommodate a nearly unlimited number of participants, but we will limit the group size to 10.

- Personalized Nutrition Program/Project (PNP) app: the PNP app will be used to self-monitor, and downloaded to the participants' smart phones for the duration of the intervention, participants will be directed to record everything that they eat or drink, hunger at the time meals are consumed (via visual analog scale), physical activity, stress, medications, sleep time and wake time. The PNP app will be pre-programmed with a: (1) weight loss target (-7% body weight); (2) hypocaloric energy target (-500 kcal/day, based on basal energy expenditure measurements from indirect calorimetry and a physical activity factor of 1.4 (lightly active); (3) dietary fat target of 25% of kcal; (4) physical activity target of 30-minutes per day. The PNP app interface will allow participants to review their records in relation to targets in real-time.

- Adherence: Study staff will routinely monitor participants' PNP dashboards, provide weekly feedback reports on self-monitoring adherence, and will be available for answering questions about meal entry to assure adherence to measurement protocol.

- Physical activity and meal timing: During baseline, 3, and 6 months, participants who choose to opt into a subgroup examining temporal eating patterns will be asked to wear activity monitors that measure physical activity, sleep, wrist-motion, and meal timing. Study staff will contact participants to assure adherence to protocol.

7.1.2 Standard of Care Study Procedures

During this study, we will monitor participants' weights and HbA1c as part of endocrinology standard of care. Participants will be reminded to continue to see their physician of record and that study procedures are not in lieu of standard medical care.

7.2 Laboratory Procedures/Evaluations

7.2.1 Clinical Laboratory Evaluations

Data are collected with visits to NYU Clinical and Translational Resource Center. Participants are contacted 2 days prior to their appointment to remind them of their measurement visit. Laboratory tests are collected, spun, refrigerated, batched and sent for processing in the CLIA-certified NYU CTSI Translational Research Laboratories by personnel blinded to group assignment. Clinically significant out of range laboratory and/or blood pressure values, as pre-defined by the safety steering committee, will be reviewed by a study clinician and the treating physician of record will be contacted for expedited notification. Participants will be asked to provide their physician's name and contact information on the informed consent form.

7.2.2 Other Assays or Procedures

Not applicable

7.2.3 Specimen Preparation, Handling, and Storage

For this study, we will be storing serum samples (two aliquots of 500µl / vial) for the first 58 participants (29 per group) having BMI≥35 kg/m2. We will not perform any genetic testing for this study. The participation for storing the samples will be optional and it is not mandatory requirement to be in research study. The samples will be stored at the Center for Biospecimen Research and Development (CBRD) research tissue bank, which is located within NYU Langone Medical Center's Office of Collaborative Science at 540 1st Avenue New York, NY 10016. The research coordinator will de-identify the name assign a unique identification number and store

it in a -80°C freezer. They will use the code number to connect subject sample to their health information that is stored in a computer database. The computer database is protected with a password.

One additional sample of plasma up to 10 ml will be collected in all groups at baseline and 6 months for future metabolomics analysis. These samples will be stored at the Laboratory of Translational Obesity Research (NYU Langone Medical Center, Science Building, 435 East 30th St, Room 623N, NY NY 10016) by Jose Aleman (and/or research team) in -80°C freezer under the discretion of the PI (Mary Sevick). Only samples from participants who have expressly given consent for future use will be included.

7.2.4 Specimen Shipment

Stool samples for the personalized mHealth group will be stored at CBRD in original OMNIgene•GUT (OMR-200) at --80°C until shipping. Personalized mHealth samples will be express shipped on dry ice via World Courier or FedEx to address listed below. We anticipate a stool shipment of 10-15 samples per month, depending on the number of participants in the cohort.

Shipping address:

Eran Segal
Wolfson Building, room 711
The Weizmann Institute of Science
234 Herzel Street, Rehovot 7610001
Israel

Labeling requirements:

1. Patient ID: PDXXX
2. PNP ID: 7 digit #
3. RegistrationCode: 5 or 6 digit #
4. TubeID: FIC_5 digit #
5. Stool collection time and date

7.3 Study Schedule

7.3.1 Screening

Telephone Screening (Day -28 to -42)

- A brief description of the study will be provided.
- Obtain verbal consent over the telephone.
- Screen participants over telephone to determine eligibility.
- If participants fail screening, they will be offered information about other studies in the department that may pertain to them.

Screening Visit (Visit 1, Day -21) at the NYU Clinical Research Center (CTSI-CRC)

- Measure height (cm) and weight (kg)
    - If participants are below or above eligible BMI range (27-50 kg/m2), participants will not be consented and will be dismissed with payment for visit.
- Obtain informed consent of potential participant verified by signature on written informed consent for screening form
    - Participants consented during the COVID-19 pandemic will be consented via Redcap e-consent framework as described in section 13.3.2.
- A copy of the informed consent will be given to eligible participants
- Review medical history to determine eligibility based on inclusion/exclusion criteria.
- Review medications history to determine eligibility based on inclusion/exclusion criteria.

CONFIDENTIAL
This material is the property of the NYU School of Medicine and Langone Medical Center. Do not disclose or use except as authorized in writing by the study sponsor

- Collect a maximum of 6.5 ml of blood to test HbA1c (<8%) and serum creatinine (eGFR >60 ml/min). This is a non-fasted visit.
- Load Personalized Nutrition Program (PNP) app onto phone and train participant in its use
- Participant enters dietary intake for 1 week into PNP app
- Provide questionnaires via Redcap sent to personal email or paper/pencil (personal habits, medical history, sociodemographic, self-efficacy)
- Obtain availability for Webex sessions
- Call participants if they are eligible for the study

7.3.2 Enrollment/Baseline

*days indicated are approximate; exact dates subject to vary up to 4 weeks pending participant availability and recruitment

Baseline Measurement Visit (Visit 2, Day -14) at the NYU Clinical and Translational Science Institute Clinical Research Center (CTSI-CRC)

- Staff reviews PNP entries with participant, exclude from further participation those who enter <2 meals/day, retrain as needed
- Collect questionnaires (if complete via paper and pencil).
- Perform a resting energy expenditure (REE) test and bioelectrical impedance analysis (BIA) test; participants will be asked if they have an implantable device- participants with implantable devices will skip the BIA measurement
- Measure waist, hip, and neck circumference.
- Insert continuous glucose monitoring device (CGM).
- Provide activity monitor.
- UNBLIND RANDOMIZATION ASSIGNMENT to determine whether testing glycemic profiling labs
    - Blood collection amounts will differ based on group allocation:
    - mHealth group
        - Collect a maximum of 10.0 ml of blood for testing measuring fasting glucose & insulin, and an additional 4.0 ml for future metabolomics analysis
    - Personalized mHealth group
        - Collect a maximum of 15.5 ml of blood for testing measuring fasting glucose, insulin and glycemic profiling labs (CBC), and an additional 4.0 ml for future metabolomics analysis
    - In first 58 participants (29 per group) having BMI≥35 kg/m2:
        - Collect an additional 27 ml of blood for testing receptor for advanced glycation end products (RAGE), advanced glycation end products (AGE), S100A8/A9 and inflammatory markers.
- Provide stool kit for participants randomized to group 2
- Schedule baseline device visit

Baseline Device Visit (Visit 3, Day -7) at the NYU Clinical and Translational Science Institute Clinical Research Center (CTSI-CRC)

- Collect CGMs
- Collect activity monitor
- Load WebEx app onto phone and train participant in joining WebEx teleconference calls via their smart phone
- 24 hour recall
- Personalized mHealth group ONLY:
    - Collect stool sample

Phase 1 Intervention period begins for all participants (Day 0)

- One-on-one phone call session with study dietitian to review intervention goals
    - mHealth: staying under recommended ceiling for calories and fat.
    - Personalized mHealth: personalized meal and snack plans and the use of real-time feedback on predicted glycemic response.

CONFIDENTIAL
This material is the property of the NYU School of Medicine and Langone Medical Center. Do not disclose or use except as authorized in writing by the study sponsor

7.3.3 Intermediate Visits

3-month visit

The 3-month visit will be identical to the baseline measurement visit will some minor modifications.

3-month visit (Visit 4, Day 91 +/-21 days at the NYU Clinical and Translational Science Institute Clinical Research Center (CTSI-CRC)

- For all participants:
  - Draw a maximum of 13.0 mL of blood to measure insulin, glucose, and HbA1c
  - Perform a resting energy expenditure (REE) test and bioelectrical impedance analysis (BIA) test; participants will be asked if they have an implantable device- participants with implantable devices will skip the BIA measurement
  - Measure waist, hip, and neck circumference.
  - Provide self-efficacy questionnaire.
  - 24 hour recall
  - Insert continuous glucose monitoring device (CGM).
  - Provide activity monitor.
  - Provide postage-paid return box for return of CGM and activity monitor to investigators.
- In first 58 participants (29 per group) having BMI≥35 kg/m2:
  - Collect an additional 27 mL of blood for testing receptor for advanced glycation end products (RAGE), advanced glycation end products (AGE), S100A8/A9 and inflammatory markers.

6-month visit

The 6-month visit will be identical to the 3-month visit with an additional blood sample collection.

6-month visit (Visit 5, Day 183 +/-21 days) at the NYU Clinical and Translational Science Institute Clinical Research Center (CTSI-CRC)

- For all participants:
  - Draw a maximum of 13.0 mL of blood to measure insulin, glucose, and HbA1c, and an additional 4.0 ml for future metabolomics analysis
  - Perform a resting energy expenditure (REE) test and bioelectrical impedance analysis (BIA) test; participants will be asked if they have an implantable device- participants with implantable devices will skip the BIA measurement
  - Measure waist, hip, and neck circumference.
  - Provide self-efficacy questionnaire.
  - 24 hour recall
  - Insert continuous glucose monitoring device (CGM).
  - Provide activity monitor
  - Provide postage-paid return box for return of CGM and activity monitor to investigators.

- In first 58 participants (29 per group) having BMI≥35 kg/m2:
  - Collect an additional 27 mL of blood for testing receptor for advanced glycation end products (RAGE), advanced glycation end products (AGE), S100A8/A9 and inflammatory markers.

7.3.4 Final Study Visit (Subgroup analysis; data collection will end October 2020)

12-month visit (Visit 6, Day 365 +/-21 days) at the NYU Clinical and Translational Science Institute Clinical Research Center (CTSI-CRC)

- For all participants:
  - Perform a resting energy expenditure (REE) test and bioelectrical impedance analysis (BIA) test; participants will be asked if they have an implantable device- participants with implantable devices will skip the BIA measurement
  - Measure waist, hip, and neck circumference.

  - Provide self-efficacy questionnaire and end of program questionnaire.
  - 24 hour recall
- In first 58 participants (29 per group) having BMI≥35 kg/m2:
  - Collect a maximum of 27 ml of blood for testing receptor for advanced glycation end products (RAGE), advanced glycation end products (AGE), S100A8/A9 and inflammatory markers.
- At the conclusion of the study or at the time of withdrawal, participants will be sent a one-page handout with their personal results, and a copy of the study primary outcomes paper.

7.3.5 Withdrawal Visit

See Handling of Participant Withdrawals or Termination

7.3.6 Unscheduled Visit

Unscheduled visits by participants will be instructed to return during scheduled visits for data collection. These visits will be documented.

7.4 Concomitant Medications, Treatments, and Procedures

All concomitant prescription medications and dosage amounts taken during study participation will be recorded on CRF. For this protocol, a prescription medication is defined as a medication that can be prescribed only by a properly authorized/licensed clinician. Medications to be reported in CRF are concomitant prescription medications, over-the-counter medications and non-prescription medications. Medication information will be collected at screening and measurement visits. Oral glycemic agents (OGAs) that are taken by participants in the study may directly or indirectly impact the primary and secondary outcomes of the study. Because all patients will continue to receive standard care, all that we can do is to monitor the medications and note any changes.

7.5 Justification for Sensitive Procedures

7.5.1 Precautionary Medications, Treatments, and Procedures

Not applicable

7.6 Prohibited Medications, Treatments, and Procedures

See exclusion criteria for list of prohibited medications

7.7 Prophylactic Medications, Treatments, and Procedures

No medications, treatments or procedures will be provided as prophylaxis

7.8 Participant Access to Study Intervention at Study Closure

Participants will receive the foods that resulted in the largest postprandial hyperglycemic excursions at conclusion of the study. They will be sent a copy of the study primary outcomes paper upon termination of the study.

CONFIDENTIAL
This material is the property of the NYU School of Medicine and Langone Medical Center. Do not disclose or use except as authorized in writing by the study sponsor

8 Assessment of Safety

8.1 Specification of Safety Parameters

8.1.1 Definition of Adverse Events (AE)

An ***adverse event*** (AE) is any symptom, sign, illness or experience that develops or worsens in severity during the course of the study. Intercurrent illnesses or injuries should be regarded as adverse events. Abnormal results of diagnostic procedures are considered to be adverse events if the abnormality:

- is associated with a serious adverse event
- is associated with clinical signs or symptoms
- leads to additional treatment or to further diagnostic tests
- is considered by the investigator to be of clinical significance

8.1.2 Definition of Serious Adverse Events (SAE)

Serious Adverse Event
Adverse events are classified as serious or non-serious. A ***serious adverse event*** is any AE that is:

- fatal
- life-threatening
- requires or prolongs hospital stay
- results in persistent or significant disability or incapacity
- a congenital anomaly or birth defect
- an important medical event

Important medical events are those that may not be immediately life threatening, but are clearly of major clinical significance. They may jeopardize the subject, and may require intervention to prevent one of the other serious outcomes noted above. For example, drug overdose or abuse, a seizure that did not result in in-patient hospitalization, or intensive treatment of bronchospasm in an emergency department would typically be considered serious.

All adverse events that do not meet any of the criteria for serious should be regarded as ***non-serious adverse events***.

8.1.3 Definition of Unanticipated Problems (UP)

Unanticipated Problems Involving Risk to Subjects or Others
Any incident, experience, or outcome that meets <u>all</u> of the following criteria:

- <u>Unexpected in nature, severity, or frequency</u> (i.e. not described in study-related documents such as the IRB-approved protocol or consent form, the investigators brochure, etc)
- <u>Related or possibly related to participation in the research</u> (i.e. possibly related means there is a reasonable possibility that the incident experience, or outcome may have been caused by the procedures involved in the research)
- <u>Suggests that the research places subjects or others at greater risk of harm</u> (including physical, psychological, economic, or social harm).

8.2 Classification of an Adverse Event

8.2.1 Severity of Event

For AEs not included in the protocol defined grading system, the following guidelines will be used to describe severity.

CONFIDENTIAL
This material is the property of the NYU School of Medicine and Langone Medical Center. Do not disclose or use except as authorized in writing by the study sponsor

- **Mild** – Events require minimal or no treatment and do not interfere with the participant's daily activities.
- **Moderate** – Events result in a low level of inconvenience or concern with the therapeutic measures. Moderate events may cause some interference with functioning.
- **Severe** – Events interrupt a participant's usual daily activity and may require systemic drug therapy or other treatment. Severe events are usually potentially life-threatening or incapacitating.

8.2.2 Relationship to Study Intervention

For all collected AEs, the clinician who examines and evaluates the participant will determine the AE's causality based on temporal relationship and his/her clinical judgment. The degree of certainty about causality will be graded using the categories below.

- **Definitely Related** – There is clear evidence to suggest a causal relationship, and other possible contributing factors can be ruled out. The clinical event, including an abnormal laboratory test result, occurs in a plausible time relationship to intervention administration and cannot be explained by concurrent disease or other drugs or chemicals. The response to withdrawal of the intervention (dechallenge) should be clinically plausible.
- **Probably Related** – There is evidence to suggest a causal relationship, and the influence of other factors is unlikely. The clinical event, including an abnormal laboratory test result, occurs within a reasonable time after administration of the intervention, is unlikely to be attributed to concurrent disease or other drugs or chemicals, and follows a clinically reasonable response on withdrawal (dechallenge). Rechallenge information is not required to fulfill this definition.
- **Possibly Related** – There is some evidence to suggest a causal relationship (e.g., the event occurred within a reasonable time after administration of the trial intervention). However, other factors may have contributed to the event (e.g., the participant's clinical condition, other concomitant events). Although an AE may rate only as "possibly related" soon after discovery, it can be flagged as requiring more information and later be upgraded to "probably related" or "definitely related," as appropriate.
- **Unlikely to be related** – A clinical event, including an abnormal laboratory test result, whose temporal relationship to intervention administration makes a causal relationship improbable (e.g., the event did not occur within a reasonable time after administration of the trial intervention) and in which other drugs or chemicals or underlying disease provides plausible explanations (e.g., the participant's clinical condition, other concomitant treatments).
- **Not Related** – The AE is completely independent of study intervention administration, and/or evidence exists that the event is definitely related to another etiology. There must be an alternative, definitive etiology documented by the clinician.

8.2.3 Expectedness

The PI (Sevick) will be responsible for determining whether an AE is expected or unexpected. An AE will be considered unexpected if the nature, severity, or frequency of the event is not consistent with the risk information previously described for the study intervention.

8.3 Time Period and Frequency for Event Assessment and Follow-Up

The occurrence of an AE or SAE may come to the attention of study personnel during study visits and interviews of a study participant presenting for medical care, or upon review by a study monitor. All AEs including local and systemic reactions not meeting the criteria for SAEs will be captured on the appropriate CRF. Information to be collected includes event description, time of onset, clinician's assessment of severity, relationship to study intervention (assessed only by those with the training and authority to make a diagnosis), and time of resolution/stabilization of the event. All AEs occurring while on study must be documented appropriately regardless of relationship. All AEs will be followed to adequate resolution.

Any medical condition that is present at the time that the participant is screened will be considered as baseline and not reported as an AE. However, if the study participant's condition deteriorates at any time

during the study, it will be recorded as an AE. UPs will be recorded in the data collection system throughout the study.

Changes in the severity of an AE will be documented to allow an assessment of the duration of the event at each level of severity to be performed. AEs characterized as intermittent require documentation of onset and duration of each episode.

The PI (Sevick) will record all reportable events with start dates occurring any time after informed consent is obtained until 7 (for non-serious AEs) or 30 days (for SAEs) after the last day of study participation. At each study visit, the investigator will inquire about the occurrence of AE/SAEs since the last visit. Events will be followed for outcome information until resolution or stabilization.

All unresolved adverse events should be followed by the investigator until the events are resolved, the subject is lost to follow-up, or the adverse event is otherwise explained. At the last scheduled visit, the investigator should instruct each subject to report any subsequent event(s) that the subject, or the subject's personal physician, believes might reasonably be related to participation in this study. The investigator should notify the study sponsor of any death or adverse event occurring at any time after a subject has discontinued or terminated study participation that may reasonably be related to this study.

8.4 Reporting Procedures – Notifying the IRB

8.4.1 Adverse Event Reporting

The PI will report an AE via Research Navigator to the IRB within 5 working days of learning about the event. The report will include a narrative summary of all events surrounding the AE.

8.4.2 Serious Adverse Event Reporting

The PI and Project Manager will oversee the study to ensure data being collected is accurate and relevant to the study question. The PI and Project Manager will monitor any safety issues that arise. Information on any office visits occurring between study visits, and changes made to medications will be carefully tracked and recorded in the appropriate sections of the Case Report Forms at the next scheduled study visit. Follow-up forms will be completed in accordance with standard AE/SAE reporting guidelines. All AE/SAEs will be evaluated for seriousness, severity, and causality by the PI, and bariatric surgeons. The IRB will be notified of reportable events on an annual basis or more often as required.

Should a patient experience a serious, unexpected adverse event (SAE), it will be reported to the IRB within 24 hours of learning of the event, using an SAE report form. As this is an exploratory study, there are no foreseeable stopping rules for the entire study at this point.

8.4.3. Unanticipated Problem Reporting

Incidents or events that meet the OHRP criteria for UPs require the creation and completion of an UP report form. It is the site investigator's responsibility to report UPs to their IRB and to the DCC/study sponsor. The UP report will include the following information:

- Protocol identifying information: protocol title and number, PI's name, and the IRB project number;
- A detailed description of the event, incident, experience, or outcome;
- An explanation of the basis for determining that the event, incident, experience, or outcome represents an UP;

This material is the property of the NYU School of Medicine and Langone Medical Center. Do not disclose or use except as authorized in writing by the study sponsor

- A description of any changes to the protocol or other corrective actions that have been taken or are proposed in response to the UP.

To satisfy the requirement for prompt reporting, UPs will be reported using the following timeline:

- UPs that are SAEs will be reported to the IRB and to the DCC/study sponsor immediately after the investigator becoming aware of the event.
- Any other UP will be reported to the IRB and to the DCC/study sponsor within 5 working days of the investigator becoming aware of the problem.
- All UPs should be reported to appropriate institutional officials (as required by an institution's written reporting procedures), the supporting agency head (or designee), and OHRP within 10 workings days of the IR's receipt of the report of the problem from the investigator.

8.4.4 Reporting of Pregnancy

If at any point during the duration of the study (i.e., screening, intervention) a participant becomes pregnant, they will be excluded from further continuation of the study.

8.5 Reporting Procedures – Notifying the Study Sponsor

The study clinician (Co-PI Bergman) will complete a SAE Form within the following timelines:

- All deaths and immediately life-threatening events, whether related or unrelated, will be recorded on the SAE Form and submitted to the DCC/study sponsor within 24 hours of site awareness. See Section 1, Key Roles for contact information.
- Other SAEs regardless of relationship will be submitted to the DCC/study sponsor within 72 hours of site awareness.

All SAEs will be followed until satisfactory resolution or until the site investigator deems the event to be chronic or the adherence to be stable. Other supporting documentation of the event may be requested by the DCC/study sponsor and should be provided as soon as possible.

As a follow-up to the initial report, within the following 48 hours of awareness of the event, the investigator shall provide further information, as applicable, on the unanticipated event or the unanticipated problem in the form of a written narrative. This should include a copy of the completed Unanticipated Problem form, and any other diagnostic information that will assist the understanding of the event. Significant new information on ongoing unanticipated adverse effects shall be provided promptly to the study sponsor.

8.6 Study Halting Rules

There are no predefined halting rules in place. We do not foresee temporary suspension of enrollment and/or study intervention due to the intent to treat nature of the study intervention.

8.7 Safety Oversight

It is the responsibility of the PI (Sevick) to oversee the safety of the study at her site. This safety monitoring will include careful assessment and appropriate reporting of adverse events as noted above, as well as the construction and implementation of a site data and safety-monitoring plan. Medical monitoring will include a regular assessment of the number and type of serious adverse events. Safety monitor reviews will be conducted a minimum of once per month and a summary report will be disseminated to the research team via hardcopy or email.

9 Clinical Monitoring

Clinical site monitoring is conducted to ensure that the rights and well-being of human subjects are protected, that the reported trial data are accurate, complete, and verifiable, and that the conduct of the trial is in compliance with the currently approved protocol/amendment(s), with GCP, and with applicable regulatory requirement(s).

- The PI (Sevick) and Project manager will monitor on-site beginning with the first subject enrolled and every week thereafter as needed.
- Independent audits will be conducted by study sponsor to ensure monitoring practices are performed correctly at the site

10 Statistical Considerations

10.1 Statistical and Analytical Plans

There is no formal SAP

10.2 Statistical Hypotheses

We expect that Weight $loss_{personalized\text{-}mHealth}$ > Weight $loss_{mHealth}$ at 6 months. We expect that these differences will be sustained at 12 months

10.3 Analysis Datasets

An "intent-to-treat" (ITT) approach will be used to address the specific aims. All participants will be included in the data analysis in the treatment arm to which they were randomized, regardless of compliance, treatment received, or deviation from protocol. Data from participants who withdraw will be used to the extent permitted by human subjects and privacy considerations

10.4 Description of Statistical Methods

10.4.1. General Approach

The formal study design is a 2 phase, 2 group randomized clinical trial. A descriptive analysis of all data collected will be performed using appropriate graphical and numerical exploratory data techniques. The information obtained from this preliminary investigation of the data will be used to: (1) assess data quality and completeness; (2) describe univariate and bivariate distributions at baseline, 3, 6 and 12 mos; and (3) identify associations between variables. We will identify features of the data that may necessitate special methods (e.g., excess zeros, missing data, departures from distributional assumptions). During preliminary analysis we will examine: (1) comparability of treatment arms at baseline (based on Chi-squared statistics or t-tests, as appropriate), (2) relationships between the response variables and potential covariates, and (3) predictors of missing data/drop-out.

The statistical modeling of the baseline, 3, 6 and 12 mos outcome variables will be based on linear mixed models. In all models, recruitment site, presence/absence of T2D, time (3 dummy variables), and intervention will be included as fixed effects, and participant will be the random effect. The intervention effect of interest is the treatment X time interaction in this model. Identified predictors of missing data will be included as covariates in this random effects framework, to provide unbiased estimates of the intervention effect under an assumption of missing at random (i.e., missingness depends on observed covariates but not on unobserved

covariates). We will conduct some sensitivity analyses to assess plausible departures from this assumption. Other demographic and clinical covariates will be included as necessary in adjusted analyses. Model assessment will be conducted using appropriate regression diagnostics. Post hoc multiple comparison tests such as Tukey or Tukey-Kramer method will be applied to adjust the pairwise comparisons across different treatment groups at single measurement time. For the longitudinal modeling we will use the resampling method to adjust the post hoc testing to address the correlated comparisons[83]. The primary and secondary analyses will be done using Stata and SAS, and MPlus will be used in the mediation analyses.

10.4.2 Analysis of the Primary Efficacy Endpoint(s)

Data analysis for primary endpoints: For hypotheses pertaining to weight loss, the outcome of interest is % of baseline body weight lost at 6 mos and whether or not these losses will be sustained at 12 mos. A random effects linear regression model will be used to test time-specific differences attributable to the intervention. We also will use the "lincom" command in Stata to estimate differences in time-specific changes from baseline. In additional analyses, we will adjust for other covariates (e.g., insulin secretion, insulin sensitivity, glycemic control, habits and history that could influence gut microbiome, and sociodemographics, and medication regimen) unbalanced between the treatment arms at baseline at p=0.10. We will use splined linear mixed models with repeated measures to compare changing trends in different periods: early intervention (0-3 months) and late intervention (3-6 mos). In this analysis, we will adjust for the covariates noted above.

10.4.3 Analysis of the Secondary Endpoint(s)

Data analysis for secondary endpoints 2.a-2.c: For hypotheses related to body fat distribution and metabolic adaptation at 6 and 12 months, and weight regain at 12 months, the random effects linear regression model will be used to test time-specific differences attributable to the intervention using a similar approach to that of analyses for the primary aim of weight loss.

Mediation analysis for secondary endpoints 3.a: Mediation analysis will be performed to assess whether, and by how much, self-efficacy mediates weight the relationship between randomization group and weight loss. We will use M-Plus to estimate appropriate structural equation models. We also will include important covariates (such as age, gender, race, and baseline T2D status) in the model and explore the possibility of multiple mediators.

Mediation analysis for secondary endpoints 3.b-3.d: Similar mediation analyses will be performed to examine the underlying biologic mechanisms (RAGE/S100A8/A9 and inflammatory markers) that influence weight loss/regain, metabolic adaptation, and fat distribution at 6 and 12 months.

10.4.4 Safety Analyses

Participants' percent weight change will be assessed at each measurement visit. Participants will be counseled by study dietitians to slow their rate of weight loss if their percent weight change is severe as defined as >7.5% in 3 months, >10% in 6 months, and >20% in one year.

All AEs will be assessed by study investigators and coded both by severity and relation to study intervention. Severity (1 = mild, 2 = moderate, 3 = severe); relation to study intervention (1 = not related, 2 = unlikely to be related, 3 = possibly related, 4 = probably related, 5 = definitely related).

10.4.5 Adherence and Retention Analyses

This material is the property of the NYU School of Medicine and Langone Medical Center. Do not disclose or use except as authorized in writing by the study sponsor

Adherence to the study intervention will be monitored by attendance to measurement visits, WebEx intervention meetings, and frequency of self-monitoring using the PNP app by the study interventionist.

10.4.6 Baseline Descriptive Statistics

Baseline characteristics between groups will be examined using descriptive statistics for sociodemographic, medical, and anthropometrics factors including age, height/weight/BMI/waist circumference, hip circumference, neck circumference, BIA output, REE, HbA1c.

10.4.7 Planned Interim Analysis

No interim analysis is planned as the trial treatment is nontoxic and based on our previous experience we don't expect the high variability of the efficacy of treatment.

10.4.7.1 Safety Review

There are no predefined halting rules in place. We do not foresee temporary suspension of enrollment and/or study intervention due to the intent to treat nature of the study intervention.

10.4.7.2 Efficacy Review

Not applicable

10.4.8 Additional Sub-Group Analyses

We will conduct additional sub-group analyses defined by gender and race/ethnicity separately. Within each subgroup, we will conduct similar analyses on both the primary and secondary endpoints as we described in section 10.4.1.

10.4.9 Multiple Comparison/Multiplicity

Post hoc multiple comparison tests such as Tukey or Tukey-Kramer method will be applied to adjust the pairwise comparisons across different treatment groups at single measurement time. For the longitudinal modeling we will use the resampling method to adjust the post hoc testing to address the correlated comparisons.

10.4.10 Tabulation of Individual Response Data

Individual participant data will be listed by time point.

10.4.11 Exploratory Analyses

Mediation analysis will be performed to assess whether, and by how much, self-efficacy mediates weight the relationship between randomization group and weight loss. We will use M-Plus to estimate appropriate structural equation models. We also will include important covariates (such as age, gender, race, and baseline T2D status) in the model and explore the possibility of multiple mediators.

Similar mediation analyses will be performed to examine the underlying biologic mechanisms (Glycemic variability, RAGE/S100A8/A9 and inflammatory markers) that influence weight loss/regain, metabolic adaptation, and fat distribution at 6 and 12 months.

CONFIDENTIAL
This material is the property of the NYU School of Medicine and Langone Medical Center. Do not disclose or use except as authorized in writing by the study sponsor

10.5 Sample Size

We will recruit 280 participants, randomize 210, and expect to retain 180. The study is powered to test the hypothesis that, at 6 months, Weight $loss_{personalized-mHealth}$ > Weight $loss_{mHealth}$. The final required sample of 164 (82 per group) is based on the assumption that we will achieve weight losses in the *mHealth* group consistent with those observed in the HHK Study (5.94%; SD=4.54%). With p=0.05, $SD_{weight\ loss}$ =4.54%, and a power of 80%, we can detect a difference between Weight $loss_{personalized-mHealth}$ and Weight $loss_{mHealth}$ as small as 2%. To account for a potential loss of about 25% of participants to drop-out prior to randomization, we will recruit 280 to the trial. To account for a potential loss of 25% after randomization, we will randomize 210 participants.

10.6 Measures to Minimize Bias

10.6.1 Enrollment/Randomization/Masking Procedures

Participants will be randomized using computer-generated permuted blocks and equal allocation to *mHealth* or *Personalized mHealth*. Rolling enrollment will occur with the goal of 12 cohorts of 15-20 participants until a sample size of 210 participants have been randomized. A new cohort will be randomized every 8 weeks during a 34-month recruitment period. Those running the statistical analysis (Huilin Li) will be blinded to participant allocation.

All study staff (except Huilin Li) will be unblinded to randomization to determine whether testing glycemic profiling. The NYU CTSI Translational Research Laboratories personnel will be blinded to group assignment. Participants will also be blinded to group assignments until the start of their respective interventions.

10.6.2 Evaluation of Success of Blinding

Not applicable

10.6.3 Breaking the Study Blind/Participant Code

Not applicable

11 Source Documents and Access to Source Data/Documents

Source data is all information, original records of clinical findings, observations, or other activities in a clinical trial necessary for the reconstruction and evaluation of the trial. Source data are contained in source documents. Examples of these original documents, and data records include: hospital records, clinical and office charts, laboratory notes, memoranda, subjects' diaries or evaluation checklists, pharmacy dispensing records, recorded data from automated instruments, copies or transcriptions certified after verification as being accurate and complete, microfiches, photographic negatives, microfilm or magnetic media, x-rays, subject files, and records kept at the pharmacy, at the laboratories, and at medico-technical departments involved in the clinical trial.

The study case report form (CRF) is the primary data collection instrument for the study. All data requested on the CRF must be recorded. All missing data must be explained. If a space on the CRF is left blank because the procedure was not done or the question was not asked, write "N/D". If the item is not applicable to the individual case, write "N/A". All entries should be printed legibly in black ink. If any entry error has been made, to correct such an error, draw a single straight line through the incorrect entry and enter the correct data above it. All such changes must be initialed and dated. DO NOT ERASE OR WHITE OUT

ERRORS. For clarification of illegible or uncertain entries, print the clarification above the item, then initial and date it.

Access to study records will be limited to IRB-approved members of the study team. The investigator will permit study-related monitoring, audits, and inspections by the IRB/EC, the sponsor, government regulatory bodies, and University compliance and quality assurance groups of all study related documents (e.g. source documents, regulatory documents, data collection instruments, study data etc.). The investigator will ensure the capability for inspections of applicable study-related facilities (e.g. pharmacy, diagnostic laboratory, etc.).

Participation as an investigator in this study implies acceptance of potential inspection by government regulatory authorities and applicable University compliance and quality assurance offices.

12 Quality Assurance and Quality Control

QC procedures will be implemented beginning with the data entry system and data QC checks that will be run on the database will be generated. Any missing data or data anomalies will be communicated to the site(s) for clarification/resolution.

Following written SOPs, the monitors will verify that the clinical trial is conducted and data are generated, documented (recorded), and reported in compliance with the protocol, GCP, and the applicable regulatory requirements (e.g., Good Laboratory Practices (GLP), Good Manufacturing Practices (GMP)).

The investigational site will provide direct access to all trial related sites, source data/documents, and reports for the purpose of monitoring and auditing by the sponsor, and inspection by local and regulatory authorities.

13 Ethics/Protection of Human Subjects

13.1 Ethical Standard

The investigator will ensure that this study is conducted in full conformity with Regulations for the Protection of Human Subjects of Research codified in 45 CFR Part 46.

13.2 Institutional Review Board

The protocol, informed consent form, recruitment materials, and all participant materials will be submitted to the IRB for review and approval. Approval of both the protocol and the consent form must be obtained before any participant is enrolled. Any amendment to the protocol will require review and approval by the IRB before the changes are implemented to the study. All changes to the consent form will be IRB approved; a determination will be made regarding whether previously consented participants need to be re-consented.

13.3 Informed Consent Process

13.3.1 Consent/Assent and Other Informational Documents Provided to Participants

Consent forms describing in detail the study intervention, study procedures, and risks are given to the participant and written documentation of informed consent is required prior to starting intervention. The following consent materials are submitted with this protocol: Telephone Consent and Informed Consent.

13.3.2 Consent Procedures and Documentation

A brief description of the study will be provided via telephone at the time of screening for the study. In order to minimize participant burden, a waiver of signed informed consent is requested for screening. We will verify eligibility. Signed informed consent will be obtained from remaining eligible participants and will apply to both phases of the study. Due to the COVID-19 pandemic, once approved by the IRB, participant will be consented via Redcap e-consent framework. Participants will be emailed a Redcap link containing both the informed consent and audio-visual consent (see attached items for links) and a Webex meeting will be scheduled to review these documents. At this time, we will review the consents with the participants and participant will electronically sign and date consents, to be followed by electronic signature and date by the consenter. All questions will be answered. A copy of both the informed consent form and audio-visual consent form electronically signed by both participant and consenter will be given to the participant and they will be encouraged to contact the PI with any and all questions that occur at any time during the conduct of the study. We will follow SOP: Informed Consent Process for Research (HRP-090). At this time, we will enroll only English-speaking participants, because the proposed software is currently only available in English.

Regardless of recruitment approach used, signed informed consent will be obtained at the screening visit, prior to obtaining measurements. Participants will be provided ample time to review the consent form. An investigator or study staff person will view each section with the participant, ask the participant if they understand each section, and clarify any questions they may have. The participant and the person obtaining consent will sign and date the consent form. A copy of the consent will be placed in the participant's medical record. Informed consent will be considered an ongoing process throughout the study, and participants' questions regarding their rights and responsibilities will be addressed whenever they occur.\

13.4 Participant and Data Confidentiality

Information to be obtained from participants includes glucose measurements from CGMS, physical activity and sleep from activity monitor, height and weight, blood tests, blood pressure, investigator-administered questionnaires (sociodemographics, comorbid conditions, and psychometric instruments), physical activity, and dietary data. None of the data to be collected is considered sensitive in nature. Some of the participant data such as laboratory results will be linked to the participant's name. Other data (e.g., height, weight, and surveys) will be linked to the participant through an ID number. We will maintain separate files for identified and de-identified data in locked file cabinets in a locked office. Access to these data will be restricted to the PI (Sevick), the medical co-Is (Dr. Bergman), the project manager, study interventionists, study staff responsible for gathering data and maintaining research files. Data will be entered into a centralized database maintained on a secure server. Data in these files will be linked to participants only through their ID number. All data collected will be used expressly for the purpose of the proposed study. Weizmann Institute of Science personnel will only have access to de-identified datasets shared through NYU's HIPAA-compliant OneDrive cloud drive. De-identified data related to actigraphy for the temporal eating pattern subgroup will be shared through Clemson University's HIPAA-compliant Box cloud drive. This will be accessed by Dr. Adam Hoover, a professor in Clemson University's Electrical and Computer Engineering Department, and his study team. The purpose of this subgroup analysis is explained in section 7.1.1.

Measures to be used to protect subjects' privacy interests are described above. Data collection will occur in a private setting where there is no opportunity for the participants' responses to be overheard. Participants will be told that they can refuse to respond to any questions that make them uncomfortable, and that they can withdraw from the study at any time.

A variety of measures will be used to prevent breaches of confidentiality. First, all study staff will be trained in the NYULMC Research Practice Fundamentals, which include training in issues of confidentiality. As private information is collected as part of this study, there is a risk to participants' privacy and confidentiality. The research staff will take every precaution to protect participants' identity and the confidentiality of the information collected.

Personalized Nutrition Project (the online dietary self-monitoring program) will be programmed by study staff with participants' age, gender, height, weight, dietary recommendations, a study ID number and a password.

Participants will be asked to enter their meals and physical activities into Personalized Nutrition Project. The app was specifically designed for study purposes, and is based on a machine-learning algorithm that integrates blood parameters, dietary habits, anthropometrics, physical activity, and gut microbiota. The Personalized Nutrition Project app is secured and data encrypted. The Personalized Nutrition Project app does not collect to transfer: a) patient-related health information or personal identifiers; b) mobile phone numbers, serial numbers or any other information that can be used to identify the user; c) GPS tracking or locality information. Participants will be directed to enter their lifestyle logs into PNP app for the next 6 months.

14 Data Handling and Record Keeping

14.1 Data Collection and Management Responsibilities

Data collection is the responsibility of the clinical trial staff at the site under the supervision of the site PI (Sevick). The investigator is responsible for ensuring the accuracy, completeness, legibility, and timeliness of the data reported.

All source documents should be completed in a neat, legible manner to ensure accurate interpretation of data. Black ink is required to ensure clarity of reproduced copies. When making changes or corrections, cross out the original entry with a single line, and initial and date the change. DO NOT ERASE, OVERWRITE, OR USE CORRECTION FLUID OR TAPE ON THE ORIGINAL.

Copies of the electronic CRF (eCRF) will be provided for use as source documents and maintained for recording data for each participant enrolled in the study. Data reported in the eCRF derived from source documents should be consistent with the source documents or the discrepancies should be explained and captured in a progress note and maintained in the participant's official electronic study record.

Clinical data (including AEs, concomitant medications, and expected adverse reactions data) and clinical laboratory data will be entered into RedCap, a data capture system provided by the NYU HHC CTSI. The data system includes password protection and internal quality checks, such as automatic range checks, to identify data that appear inconsistent, incomplete, or inaccurate. Clinical data will be entered directly from the source documents.

Some of the participant data such as laboratory results will be linked to the participant's name in Epic. Other data (e.g., height, weight, and surveys) will be linked to the participant through an ID number. We will maintain separate files for identified and de-identified data in locked file cabinets in a locked office. Access to these data will be restricted to the PI (Sevick) and her staff - all of whom will be trained in ethical research practices. Data will be entered into a centralized database maintained on a secure, password- protected server. Data in these files will be linked to participants only through their ID number. All data collected will be used expressly for the purpose of the proposed study. The specimens and/or associated data will be banked. Serum samples will be hand-delivered by study staff who travel by foot to NYULMC laboratories. Serum samples will be analyzed and then stored in the bio-repository center under the direction of Dr. Rachel Brody. Results of lab testing will be available on Epic. No one other than study staff, NYULMC laboratory personnel, and providers of record will have access to the specimens or their results.

14.2 Study Records Retention

Study documents will be retained for the longer of 3 years after close out or 5 years after final reporting/publication. These documents should be retained for a longer period, however, if required by local regulations. No records will be destroyed without the written consent of the sponsor, if applicable. It is the responsibility of the sponsor to inform the investigator when these documents no longer need to be retained.

CONFIDENTIAL
This material is the property of the NYU School of Medicine and Langone Medical Center. Do not disclose or use except as authorized in writing by the study sponsor

14.3 Protocol Deviations

A protocol deviation is any noncompliance with the clinical trial protocol, GCP, or MOP requirements. The noncompliance may be either on the part of the participant, the investigator, or the study site staff. As a result of deviations, corrective actions are to be developed by the site and implemented promptly.

These practices are consistent with ICH E6:

- 4.5 Compliance with Protocol, sections 4.5.1, 4.5.2, and 4.5.3
- 5.1 Quality Assurance and Quality Control, section 5.1.1
- 5.20 Noncompliance, sections 5.20.1, and 5.20.2.

It is the responsibility of the site to use continuous vigilance to identify and report deviations within 30 working days of identification of the protocol deviation, or within 30 working days of the scheduled protocol-required activity. All deviations must be addressed in study source documents, reported to AHA Program Official, and the NYU HHC CTSI. Protocol deviations must be reported to the local IRB per their guidelines. The site PI/study staff is responsible for knowing and adhering to their IRB requirements. Further details about the handling of protocol deviations will be included in the MOP.

14.4 Publication and Data Sharing Policy

This study will comply with the NIH Public Access Policy, which ensures that the public has access to the published results of NIH funded research. It requires scientists to submit final peer-reviewed journal manuscripts that arise from NIH funds to the digital archive PubMed Central upon acceptance for publication.

The International Committee of Medical Journal Editors (ICMJE) member journals have adopted a clinical trials registration policy as a condition for publication. The ICMJE defines a clinical trial as any research project that prospectively assigns human subjects to intervention or concurrent comparison or control groups to study the cause-and-effect relationship between a medical intervention and a health outcome. Medical interventions include drugs, surgical procedures, devices, behavioral treatments, process-of-care changes, and the like. Health outcomes include any biomedical or health-related measures obtained in patients or participants, including pharmacokinetic measures and adverse events. The ICMJE policy, and the Section 801 of the Food and Drug Administration Amendments Act of 2007, requires that all clinical trials be registered in a public trials registry such as ClinicalTrials.gov, which is sponsored by the National Library of Medicine. Other biomedical journals are considering adopting similar policies. For interventional clinical trials performed under NIH IC grants and cooperative agreements, it is the grantee’s responsibility to register the trial in an acceptable registry, so the research results may be considered for publication in ICMJE member journals. The ICMJE does not review specific studies to determine whether registration is necessary; instead, the committee recommends that researchers who have questions about the need to register err on the side of registration or consult the editorial office of the journal in which they wish to publish.

FDAAA mandates that a "responsible party" (i.e., the sponsor or designated principal investigator) register and report results of certain "applicable clinical trials":

- Trials of Drugs and Biologics: Controlled, clinical investigations, other than Phase I investigations of a product subject to FDA regulation;
- Trials of Devices: Controlled trials with health outcomes of a product subject to FDA regulation (other than small feasibility studies) and pediatric postmarket surveillance studies.
- NIH grantees must take specific steps to ensure compliance with NIH implementation of FDAAA.

15 Study Finances

15.1 Funding Source

This study is financed through a grant from the American Heart Association. The temporal eating pattern ancillary study is funded through NYU's CTSI-CRC.

15.2 Costs to the Participant

Neither the patient nor the patient's health insurance will be billed for any study activities, tests, or procedures. The cost of all procedures and tests will be covered by funds received from the American Heart Association.

15.3 Participant Reimbursements or Payments

Participants will be paid $30.00 for each of the 6 screening and measurement visits as compensation for their time. As reimbursement for cell phone data usage, participants will also be paid $5.00 for each of the 14 WebEx educational sessions they attend, for a total of up to $70.00 at their final visit. If a participant's CGM falls off, they will be reimbursed $5.50 for their round trip public transportation. If participants opt in to the subgroup examining temporal eating patterns, they will receive $5 for opting in, and an additional $10 per baseline, 3 and 6-month measurement visits, for a total of an additional $35.

16 Study Administration

16.1 Study Leadership

Not applicable

17 Conflict of Interest Policy

The independence of this study from any actual or perceived influence, such as by the pharmaceutical industry, is critical. Therefore, any actual conflict of interest of persons who have a role in the design, conduct, analysis, publication, or any aspect of this trial will be disclosed and managed. Furthermore, persons who have a perceived conflict of interest will be required to have such conflicts managed in a way that is appropriate to their participation in the trial. The study leadership in conjunction with the AHA has established policies and procedures for all study group members to disclose all conflicts of interest and will establish a mechanism for the management of all reported dualities of interest.

Any investigator who has a conflict of interest with this study (patent ownership, royalties, or financial gain greater than the minimum allowable by their institution, etc.) must have the conflict reviewed by the NYU Langone Conflict of Interest Committee with a Committee-sanctioned conflict management plan that has been reviewed and approved by the study sponsor prior to participation in this study. All NYULMC investigators will follow the applicable conflict of interest policies.

18 References

1. Flegal KM, Carroll MD, Kit BK, Ogden CL. Prevalence of obesity and trends in the distribution of body mass index among US adults, 1999-2010. *Jama* 2012; **307**(5): 491-7.
2. Cawley J, Meyerhoefer C. The medical care costs of obesity: an instrumental variables approach. *Journal of health economics* 2012; **31**(1): 219-30.
3. Hardy OT, Czech MP, Corvera S. What causes the insulin resistance underlying obesity? *Current opinion in endocrinology, diabetes, and obesity* 2012; **19**(2): 81-7.
4. Knowler WC, Barrett-Connor E, Fowler SE, et al. Reduction in the incidence of type 2 diabetes with lifestyle intervention or metformin. *The New England journal of medicine* 2002; **346**(6): 393-403.
5. Wing RR, Lang W, Wadden TA, et al. Benefits of Modest Weight Loss in Improving Cardiovascular Risk Factors in Overweight and Obese Individuals With Type 2 Diabetes. *Diabetes Care* 2011; **34**(7): 1481-6.
6. Lazo M, Solga SF, Horska A, et al. Effect of a 12-month intensive lifestyle intervention on hepatic steatosis in adults with type 2 diabetes. *Diabetes Care* 2010; **33**(10): 2156-63.
7. The Look ARG, Knowler WC, Bahnson JL, et al. Effect of a Long-Term Behavioral Weight Loss Intervention on Nephropathy in Overweight or Obese Adults with Type 2 Diabetes: the Look AHEAD Randomized Clinical Trial. *The lancet Diabetes & endocrinology* 2014; **2**(10): 801-9.
8. Rubin RR, Wadden TA, Bahnson JL, et al. Impact of intensive lifestyle intervention on depression and health-related quality of life in type 2 diabetes: the Look AHEAD Trial. *Diabetes Care* 2014; **37**(6): 1544-53.
9. Foster GD, Borradaile KE, Sanders MH, et al. A randomized study on the effect of weight loss on obstructive sleep apnea among obese patients with type 2 diabetes: the Sleep AHEAD study. *Archives of internal medicine* 2009; **169**(17): 1619-26.
10. Redmon JB, Bertoni AG, Connelly S, et al. Effect of the look AHEAD study intervention on medication use and related cost to treat cardiovascular disease risk factors in individuals with type 2 diabetes. *Diabetes Care* 2010; **33**(6): 1153-8.
11. Goldstein DJ. Beneficial health effects of modest weight loss. *International journal of obesity and related metabolic disorders : journal of the International Association for the Study of Obesity* 1992; **16**(6): 397-415.
12. Feldstein AC, Nichols GA, Smith DH, et al. Weight change in diabetes and glycemic and blood pressure control. *Diabetes Care* 2008; **31**(10): 1960-5.
13. Shah K, Villareal DT. Weight loss and improved fitness slow down the decline in mobility in obese adults with type 2 diabetes. *Evidence-Based Medicine* 2013; **18**(2): e18-e.
14. Wing RR, Rosen RC, Fava JL, et al. Effects of weight loss intervention on erectile function in older men with type 2 diabetes in the Look AHEAD trial. *The journal of sexual medicine* 2010; **7**(1 Pt 1): 156-65.
15. Guyenet S. Testing the Insulin Model: A Response to Dr. Ludwig. Whole Health Source. Nutrition & Health Science. *http://wholehealthsourceblogspotcom/2016/01/testing-insulin-model-response-to-drhtml* 2016.
16. Ludwig D. Ludwig Responds to Whole Health Source Article. *https://mediumcom/@davidludwigmd/ludwig-responds-to-whole-health-source-article-93d8e1667477#y6tdz6j07* 2016.
17. Karpe F, Dickmann JR, Frayn KN. Fatty acids, obesity, and insulin resistance: time for a reevaluation. *Diabetes* 2011; **60**(10): 2441-9.
18. Blaak EE, Antoine JM, Benton D, et al. Impact of postprandial glycaemia on health and prevention of disease. *Obesity reviews : an official journal of the International Association for the Study of Obesity* 2012; **13**(10): 923-84.
19. Chiasson JL, Josse RG, Gomis R, Hanefeld M, Karasik A, Laakso M. Acarbose for prevention of type 2 diabetes mellitus: the STOP-NIDDM randomised trial. *Lancet (London, England)* 2002; **359**(9323): 2072-7.
20. Chiasson JL, Josse RG, Gomis R, Hanefeld M, Karasik A, Laakso M. Acarbose treatment and the risk of cardiovascular disease and hypertension in patients with impaired glucose tolerance: the STOP-NIDDM trial. *Jama* 2003; **290**(4): 486-94.
21. Hanefeld M, Chiasson JL, Koehler C, Henkel E, Schaper F, Temelkova-Kurktschiev T. Acarbose slows progression of intima-media thickness of the carotid arteries in subjects with impaired glucose tolerance. *Stroke; a journal of cerebral circulation* 2004; **35**(5): 1073-8.
22. Esposito K, Giugliano D, Nappo F, Marfella R. Regression of carotid atherosclerosis by control of postprandial hyperglycemia in type 2 diabetes mellitus. *Circulation* 2004; **110**(2): 214-9.

CONFIDENTIAL
This material is the property of the NYU School of Medicine and Langone Medical Center. Do not disclose or use except as authorized in writing by the study sponsor

23. Jenkins DJ, Wolever TM, Taylor RH, et al. Glycemic index of foods: a physiological basis for carbohydrate exchange. *The American journal of clinical nutrition* 1981; **34**(3): 362-6.
24. Jensen MD, Ryan DH, Apovian CM, et al. 2013 AHA/ACC/TOS Guideline for the Management of Overweight and Obesity in Adults. *A Report of the American College of Cardiology/American Heart Association Task Force on Practice Guidelines and The Obesity Society* 2013.
25. Freedhoff Y, Hall KD. Weight loss diet studies: we need help not hype. *Lancet (London, England)* 2016; **388**(10047): 849-51.
26. Vega-Lopez S, Ausman LM, Griffith JL, Lichtenstein AH. Interindividual variability and intra-individual reproducibility of glycemic index values for commercial white bread. *Diabetes Care* 2007; **30**(6): 1412-7.
27. Wolever TM, Vorster HH, Bjorck I, et al. Determination of the glycaemic index of foods: interlaboratory study. *European journal of clinical nutrition* 2003; **57**(3): 475-82.
28. Williams SM, Venn BJ, Perry T, et al. Another approach to estimating the reliability of glycaemic index. *The British journal of nutrition* 2008; **100**(2): 364-72.
29. Backhed F, Ding H, Wang T, et al. The gut microbiota as an environmental factor that regulates fat storage. *Proceedings of the National Academy of Sciences of the United States of America* 2004; **101**(44): 15718-23.
30. Turnbaugh PJ, Ley RE, Mahowald MA, Magrini V, Mardis ER, Gordon JI. An obesity-associated gut microbiome with increased capacity for energy harvest. *Nature* 2006; **444**(7122): 1027-131.
31. Suez J, Korem T, Zeevi D, et al. Artificial sweeteners induce glucose intolerance by altering the gut microbiota. *Nature* 2014; **514**(7521): 181-6.
32. Zhang X, Shen D, Fang Z, et al. Human Gut Microbiota Changes Reveal the Progression of Glucose Intolerance. *PLoS ONE* 2013; **8**(8): e71108.
33. Le Chatelier E, Nielsen T, Qin J, et al. Richness of human gut microbiome correlates with metabolic markers. *Nature* 2013; **500**(7464): 541-6.
34. Karlsson FH, Tremaroli V, Nookaew I, et al. Gut metagenome in European women with normal, impaired and diabetic glucose control. *Nature* 2013; **498**(7452): 99-103.
35. Qin J, Li Y, Cai Z, et al. A metagenome-wide association study of gut microbiota in type 2 diabetes. *Nature* 2012; **490**(7418): 55-60.
36. Vrieze A, Van Nood E, Holleman F, et al. Transfer of intestinal microbiota from lean donors increases insulin sensitivity in individuals with metabolic syndrome. *Gastroenterology* 2012; **143**(4): 913-6.e7.
37. Bray MS, Loos RJ, McCaffery JM, et al. NIH working group report-using genomic information to guide weight management: From universal to precision treatment. *Obesity (Silver Spring, Md)* 2016; **24**(1): 14-22.
38. Fothergill E GJ, Howard L, Kerns JC, Knuth ND, Brychta R, Chen KY, Skarulis MC, Walter M, Walter PJ, Hall KD. . Persistent metabolic adapttation 6 years after "The Biggest Loser" competition. *Obesity* 2016; **24**(8).
39. Brand-Miller JC HS, Pawlak DB, McMillan J. Glycemic index and obesity. *The American journal of clinical nutrition* 2002; **76**((suppl)): 281S-5S.
40. Bandura A. Social foundations of thought and action: A social cognitive theory: Prentice-Hall, Inc; 1986.
41. Bandura A. Self-efficacy: The exercise of control: Macmillan; 1997.
42. Shiffman S, Stone AA, Hufford MR. Ecological momentary assessment. *Annual review of clinical psychology* 2008; **4**: 1-32.
43. Heron KE, Smyth JM. Ecological momentary interventions: incorporating mobile technology into psychosocial and health behaviour treatments. *British journal of health psychology* 2010; **15**(Pt 1): 1-39.
44. Cain AE, Depp CA, Jeste DV. Ecological Momentary Assessment in Aging Research: A Critical Review. *Journal of psychiatric research* 2009; **43**(11): 987-96.
45. Zhang L, Chen B, Tang L. Metabolic memory: mechanisms and implications for diabetic retinopathy. *Diabetes research and clinical practice* 2012; **96**(3): 286-93.
46. Monnier L, Mas E, Ginet C, et al. Activation of oxidative stress by acute glucose fluctuations compared with sustained chronic hyperglycemia in patients with type 2 diabetes. *Jama* 2006; **295**(14): 1681-7.
47. Fukami K, Yamagishi S, Okuda S. Role of AGEs-RAGE system in cardiovascular disease. *Current pharmaceutical design* 2014; **20**(14): 2395-402.
48. Yamagishi S, Matsui T. Advanced glycation end products, oxidative stress and diabetic nephropathy. *Oxidative medicine and cellular longevity* 2010; **3**(2): 101-8.
49. Nemet I, Turk Z, Duvnjak L, Car N, Varga-Defterdarovic L. Humoral methylglyoxal level reflects glycemic fluctuation. *Clinical biochemistry* 2005; **38**(4): 379-83.

50. Jethwani K, Kvedar J, Kvedar J. Behavioral phenotyping: a tool for personalized medicine. *Personalized Medicine* 2010; **7**(6): 689-93.
51. Sevick MA, Zickmund S, Korytkowski M, et al. Design, feasibility, and acceptability of an intervention using personal digital assistant-based self-monitoring in managing type 2 diabetes. *Contemporary clinical trials* 2008; **29**(3): 396-409.
52. Hausmann LRM, Ren D, Sevick MA. Racial differences in diabetes-related psychosocial factors and glycemic control in patients with type 2 diabetes. *Patient preference and adherence* 2010; **4**: 291-9.
53. Sevick MA, Korytkowski M, Stone RA, et al. Biophysiologic outcomes of the Enhancing Adherence in Type 2 Diabetes (ENHANCE) trial. *Journal of the Academy of Nutrition and Dietetics* 2012; **112**(8): 1147-57.
54. Sevick MA, Stone RA, Zickmund S, Wang Y, Korytkowski M, Burke LE. Factors associated with probability of personal digital assistant-based dietary self-monitoring in those with type 2 diabetes. *Journal of behavioral medicine* 2010; **33**(4): 315-25.
55. Sevick MA, Stone RA, Novak M, et al. A PDA-based dietary self-monitoring intervention to reduce sodium intake in an in-center hemodialysis patient. *Patient preference and adherence* 2008; **2**: 177-84.
56. Hausmann L, Ren D, Sevick MA. Access to Chronic Illness Resources and their Relevance to Racial Differences in Glycemic Control. *Diabetes* 2008; **1850-P**.
57. Wang J, Burke LE, Steenkiste A, Sevick M. Technology-supported diabetes self-management intervention improved weight loss through enhancing self-efficacy. *Diabetes* 2011; **60(Supplement 1): A62.**
58. Provenzano LF, Piraino B, Steenkiste A, Sevick MA. 91 Sodium Intake in Type 2 DM Is High. *American Journal of Kidney Diseases* 2011; **57**(4): B39.
59. Hu L, Woolf K, David S-J, et al. Multicomponent Lifestyle Intervention in Obese Patients with Diabetes and Chronic Kidney Disease. *Society for Behavioral Medicine [accepted]* 2016.
60. Zeevi D, Korem T, Rothschild D, et al. Personalized Nutrition by Prediction of Glycemic Responses. *Cell* 2015; **163**(5): 1079-94.
61. Santacruz A, Marcos A, Warnberg J, et al. Interplay between weight loss and gut microbiota composition in overweight adolescents. *Obesity (Silver Spring, Md)* 2009; **17**(10): 1906-15.
62. Ridaura VK, Faith JJ, Rey FE, et al. Gut microbiota from twins discordant for obesity modulate metabolism in mice. *Science* 2013; **341**(6150): 1241214.
63. Turnbaugh PJ, Hamady M, Yatsunenko T, et al. A core gut microbiome in obese and lean twins. *Nature* 2009; **457**(7228): 480-4.
64. Everard A, Lazarevic V, Gaia N, et al. Microbiome of prebiotic-treated mice reveals novel targets involved in host response during obesity. *The ISME journal* 2014; **8**(10): 2116-30.
65. Monnier L, Colette C, Dunseath GJ, Owens DR. The loss of postprandial glycemic control precedes stepwise deterioration of fasting with worsening diabetes. *Diabetes Care* 2007; **30**(2): 263-9.
66. Monnier L LH, Collette C. Contributions of fasting and postprandial plasma glucose increments to the overall diurnal hyperglycemia of type 2 diabetic patients: variations with increasing levels of HbA(1c). *Diabetes Care* 2003; **26**(3): 881-5.
67. CDC. Overweight and Obesity New York State Adults 2012. *https://wwwhealthnygov/statistics/brfss/reports/docs/1401_overweight_and_obesitypdf* 2012.
68. Bandura A. Social cognitive theory: An agentic perspective. *Asian journal of social Psychology* 1999; **2**(1): 21-41.
69. Bandura A. Self-efficacy: Wiley Online Library.
70. Hoerger TJ, Crouse WL, Zhuo X, Gregg EW, Albright AL, Zhang P. Medicare's intensive behavioral therapy for obesity: an exploratory cost-effectiveness analysis. *American journal of preventive medicine* 2015; **48**(4): 419-25.
71. Suez J, Korem T, Zeevi D, et al. Artificial sweeteners induce glucose intolerance by altering the gut microbiota. *Nature* 2014; **514**(7521): 181-6.
72. Edgar RC. UPARSE: highly accurate OTU sequences from microbial amplicon reads. *Nature methods* 2013.
73. Korem T, Zeevi D, Suez J, et al. Growth dynamics of gut microbiota in health and disease inferred from single metagenomic samples. *Science (New York, NY)* 2015; **349**(6252): 1101-6.
74. Kanehisa M, Goto S, Kawashima S, Nakaya A. The KEGG databases at GenomeNet. *Nucleic acids research* 2002; **30**(1): 42-6.
75. Clark MM, Abrams DB, Niaura RS, Eaton CA, Rossi JS. Self-efficacy in weight management. *Journal of consulting and clinical psychology* 1991; **59**(5): 739-44.

76. Clarke W, Kovatchev B. Statistical Tools to Analyze Continuous Glucose Monitor Data. *Diabetes Technology & Therapeutics* 2009; **11**(Suppl 1): S-45-S-54.
77. McDonnell CM, Donath SM, Vidmar SI, Werther GA, Cameron FJ. A novel approach to continuous glucose analysis utilizing glycemic variation. *Diabetes Technol Ther* 2005; **7**(2): 253-63.
78. Hill NR, Oliver NS, Choudhary P, Levy JC, Hindmarsh P, Matthews DR. Normal Reference Range for Mean Tissue Glucose and Glycemic Variability Derived from Continuous Glucose Monitoring for Subjects Without Diabetes in Different Ethnic Groups. *Diabetes Technology & Therapeutics* 2011; **13**(9): 921-8.
79. Parikh M, Chung M, Sheth S, et al. Randomized pilot trial of bariatric surgery versus intensive medical weight management on diabetes remission in type 2 diabetic patients who do NOT meet NIH criteria for surgery and the role of soluble RAGE as a novel biomarker of success. *Annals of surgery* 2014; **260**(4): 617-22; discussion 22-4.
80. Nagareddy PR, Kraakman M, Masters SL, et al. Adipose tissue macrophages promote myelopoiesis and monocytosis in obesity. *Cell metabolism* 2014; **19**(5): 821-35.
81. McAuley KA, Mann JI, Chase JG, Lotz TF, Shaw GM. Point: HOMA--satisfactory for the time being: HOMA: the best bet for the simple determination of insulin sensitivity, until something better comes along. *Diabetes Care* 2007; **30**(9): 2411-3.
82. Hockaday D, Sayyad M, Yajnik C. Counterpoint: appreciating homeostasis model assessment: more useful earlier rather than later. *Diabetes Care* 2007; **30**(9): 2414-8.
83. Liu C, Cripe TP, Kim MO. Statistical issues in longitudinal data analysis for treatment efficacy studies in the biomedical sciences. *Molecular therapy : the journal of the American Society of Gene Therapy* 2010; **18**(9): 1724-30.
84. Vlassara H, Cai W, Tripp E, et al. Oral AGE restriction ameliorates insulin resistance in obese individuals with the metabolic syndrome: a randomised controlled trial. *Diabetologia* 2016; **59**(10): 2181-92.

19 Attachments

These documents are relevant to the protocol, but they are not considered part of the protocol. They are stored and modified separately. As such, modifications to these documents do not require protocol amendments.

- Telephone consent form
- Consent form
- Waiver of consent
- Abbreviated informed consent
- AV consent form
- Data stored for future use appendix
- Recruitment advertisements
- Weight Efficacy Life-Style Questionnaire
- Questionnaires
- Supporting documents/manuals for device and apps
- Physician letter for physical activity clearance

This material is the property of the NYU School of Medicine and Langone Medical Center. Do not disclose or use except as authorized in writing by the study sponsor
