# Supplementary material for: Effect of a Personalized Diet to Reduce Postprandial Glycemic Response vs a Low-fat Diet on Weight Loss in Adults With Abnormal Glucose Metabolism and Obesity: A Randomized Clinical Trial
Source: JAMA Netw Open. 2022 Sep 28;5(9):e2233760. doi: 10.1001/jamanetworkopen.2022.33760 (PMC9520362; doi:10.1001/jamanetworkopen.2022.33760)
Supplement: Supplement 2. — eMethods. Study Procedures and Measures eTable 1. Baseline Demographics, Anthropometrics, and Metabolic Variables Between Study Completers and Dropouts eTable 2. Group Comparisons of Weight Loss Within Participants Enrolled Before and After the COVID-19 Pandemic eTable 3. Baseline Dietary Intake Between the Standardized and Personalized Groups eTable 4. Changes in Dietary Measures From Baseline (Month 0) to 3 and 6 Months eTable 5. End-of-Study Questionnaire eFigure 1. Schematic of Study Design eFigure 2. Weight Loss Variability Between the Standardized (Top) and Personalized (Bottom) Groups eReferences [file jamanetwopen-e2233760-s002.pdf]

Supplementary Online Content

Popp CJ, Hu L, Kharmats AY, et al. Effect of a personalized diet to reduce postprandial glycemic response vs a low-fat diet on weight loss in adults with abnormal glucose metabolism and obesity: a randomized clinical trial. *JAMA Netw Open*. 2022;5(9):e2233760. doi:10.1001/jamanetworkopen.2022.33760

eMethods. Study Procedures and Measures

eTable 1. Baseline Demographics, Anthropometrics, and Metabolic Variables Between Study Completers and Dropouts

eTable 2. Group Comparisons of Weight Loss Within Participants Enrolled Before and After the COVID-19 Pandemic

eTable 3. Baseline Dietary Intake Between the Standardized and Personalized Groups

eTable 4. Changes in Dietary Measures From Baseline (Month 0) to 3 and 6 Months

eTable 5. End-of-Study Questionnaire

eFigure 1. Schematic of Study Design

eFigure 2. Weight Loss Variability Between the Standardized (Top) and Personalized (Bottom) Groups

eReferences

This supplementary material has been provided by the authors to give readers additional information about their work.

© 2022 Popp CJ et al. *JAMA Network Open*.

eMethods. Study Procedures and Measures

1.1 Recruitment procedure

Detailed methods regarding the protocol for the Personal Diet Study have been published elsewhere.[1] Participants were recruited primarily via the (NYU Langone Health) NYULH MyChart alerts. An IRB-approved patient-facing message was sent to potentially eligible patients' MyCharts, including a brief study description with directions to contact study staff for questions or to express their interest in participation. Self-referrals were also received from other sources including ClinicalTrials.gov and a NYULH supported website iConnect, where patients can explore active clinical trials conducted at NYULH and self-refer to the study. Study staff contacted self-referred potential participants by telephone to confirm interest, and screen for eligibility under an IRB-approved waiver of consent. Written informed consent was subsequently obtained from all participants.

1.2 Modifications to the Personal Diet Study in response to COVID-19

Prior to COVID-19, recruitment, phone-based pre-screening, and the study intervention as were performed remotely, but broader eligibility screenings, consents, measurements (baseline, 3 months, 6 months) and technology trainings were conducted in-person. After COVID-19, all study activities were modified for remote performance. In particular, measures of fat mass (FM) and fat free mass (FFM) using bioelectrical impedance analysis and resting energy expenditure (REE) using indirect calorimetry were discontinued. Energy requirements were calculated using the Mifflin St-Jeor equation with an activity factor of 1.4.[2]

Recruitment and enrollment: Self-referred individuals who were potentially eligible and interested in joining the study were scheduled for a WebEx videoconference appointment for in-depth eligibility screening and informed consent with the RDA. A WebEx link for their appointment was sent to the participant's personal email address. Potential participants joined the appointment by clicking on the link and downloading the software to their device (smartphone, personal computer, or tablet), after which they were connected to the videoconference. If deemed eligible, during the videoconference, an NYULH RedCap link to the study consent form was emailed to them. The participant was directed to open the link and read along during the consent process. Those who agreed to enroll in the study were guided to share their screen with the RDA, who witnessed the signing of the consent as the participant affixed their electronic signature. A PDF copy of the signed consent was downloaded from RedCap and sent to the participant via email.

Remote Training: In-person PNP app training was adapted for delivery via WebEx. To accomplish this, the RDA initiated a WebEx meeting with the participant via the RDA's computer. The RDA simultaneously joined the WebEx meeting using their smartphone, sharing the smartphone screen to demonstrate how to maneuver through the PNP app.

Remote measurement visit of body weight, waist and hip circumferences: As previously mentioned, remote measurement visits were delivered via WebEx in place of in-person visits at the CTSI. At each remote measurement visit, under the guidance of a Research Data Associate, the participants we instructed to remove excess clothing, jewelry, and shoes. Participants stepped on the Bluetooth-enabled Renpho Scale (RENPHO, Anaheim, CA) and their body weight was recorded, automatically uploaded to their Renpho account and the RDA confirmed the body weight was uploaded. Study staff remotely monitored body weights over the course of the study. For waist and hip measurements, we applied the same methods as in-person; however, each participant performed the circumference measurements (waist and hip) on their own with verbal and visual guidance from the RDA via WebEx.

1.3 Feedback reports

Feedback reports were sent via email to participants weekly for the first month and then every other week for the next 5 months. Reports included visuals on the number of days the participant was adherent to self-monitoring (defined as days when the participant logged at least 50% of their caloric goal), and graphical presentation of observed versus predicted individual weight loss trajectories.[3] Participants in the Personalized arm received a weekly meal score summary as a percent of total meals logged (displayed as a pie chart per week).

1.4 Calculation of Adaptive thermogenesis

Adaptive thermogenesis (AT) was calculated using previous methods taking the difference between measured REE and REE predicted (see below).[4]

© 2022 Popp CJ et al. *JAMA Network Open*.

AT = (REE measured at 3 and 6 mos) – (REE predicted at 3 and 6 mos)

Predicted REE (kcal/d) = 1000.53 – (4.421*age) - (306.712*gender [0=male; 1=female] + (8.079*FM [kg]) + (16.516*FFM [kg])

Adjusted R^2=0.7301; Residual SE ± 203.2 kcal/d; p <0.0001

1.5 Dietary Intake

At baseline, 3 and 6-month timepoints, a single Automated Self-Administered 24-hour (ASA24) Dietary was collecting in participants from both arms. Participants reported dietary intake from midnight to midnight of the previous day. Food and beverage items reported were automatically converted to energy and nutrient values using the USDA's Food and Nutrient Database for Dietary Studies.

© 2022 Popp CJ et al. *JAMA Network Open*.

eTable 1. Baseline Demographics, Anthropometrics, and Metabolic Variables Between Study Completers and Dropouts

Variable	Completers (n=155)	Dropouts (n=44)	P value
Arm, No. (%)			0.168
Standardized	71 (45.8%)	26 (59.1%)	
Personalized	84 (54.2%)	18 (40.9%)	
Sex, No. (%)			
Female	103 (66.5%)	30 (68.2%)	0.973
Male	52 (33.5%)	14 (31.8%)	
Age, mean (SD), y	59 (10)	54 (12)	**0.02**
Race, No. (%)			0.554
White	87 (56.1%)	21 (47.7%)	
African American	36 (23.2%)	13 (29.5%)	
Other	30 (19.4%)	10 (22.7%)	
Ethnicity			0.26
Non-Hispanic	130 (83.9%)	33 (77.0%)	
Hispanic	25 (16.1%)	11 (25.0%)	
Highest level of education achieved, No (%)			0. 319
Less than High	20 (12.9%	9 (20.5%)	
School	10 (6.5%)	4 (9.1%)	
High School	10 (6.5%)	3 (6.8%)	
Associate Degree	36 (23.2%)	14 (31.8%)	
Technical			
Degree/certificate	55 (35.5%)	8 (18.2%)	
Master's degree	15 (9.7%)	5 (11.4%)	
Doctoral or			
Professional	9 (5.8%)	1 (2.3%)	
Missing			
Income, per year, No (%)			0.152
<$10,000	0 (0%)	1 (2.3%)	
$10,000 - $19,000	4 (2.6%)	0 (0%)	
$20,000 - $29,999	2 (1.3%)	2 (4.5%)	
$30,000 - $39,999	9 (5.8%)	1 (2.3%)	
$40,000 - $49,999	9 (5.8%)	0 (0%)	
$50,000 - $74,999	28 (18.1%)	11 (25.0%)	
$75,000 - $99,999	24 (15.5%)	6 (13.6%)	
>$100,000	57 (36.8%)	19 (43.2%)	
Missing	22 (14.2%)	4 (9.1%)	
Body weight, kg	92.8 (16.4)	99.5 (18.2)	**0.03**
Body mass index, kg/m^2	33.4 (4.61)	35.7 (5.16)	**0.009**
Body fat, %	40.4 (7.71)	42.0 (8.39)	0.291
FM, kg	37.4 (9.34)	42.7 (12.0)	**0.04**
FFM, kg	55.3 (12.1)	57.2 (12.4)	0.407
REE, kcal/d	1750 (387)	1850 (397)	0.172
RQ	0.83 (0.116)	0.84 (0.10)	0.675
HbA1c, %	5.80 (0.588)	5.77 (0.521)	0.693
Metformin use, No. (%)	32 (20.6%)	10 (22.7%)	0.929

Abbreviations: AT, adaptive thermogenesis; BMI, body mass index; FFM, fat free mass; FM, fat mass; REE, resting energy expenditure; RQ, respiratory quotient.
*Significant between Completers and Drop outs are in bold; p< 0.05

© 2022 Popp CJ et al. *JAMA Network Open*.

eTable 2. Group Comparisons of Weight Loss Within Participants Enrolled Before and After the COVID-19 Pandemic

	Pre-COVID[a] Mean (SD)	Post-COVID[b] Mean (SD)	p-value
Weight loss (kg)			
0 to 3 mo	-2.02 (3.87)	-2.31 (4.31)	0.692
3 to 6 mo	-1.31 (4.02)	-0.533 (2.57)	0.241
0 to 6 mo	-3.65 (5.17)	-2.77 (5.49)	0.374
Weight loss (%)			
0 to 3 mo	-2.17 (4.31)	-2.52 (4.62)	0.666
3 to 6 mo	-1.35 (4.11)	-0.589 (2.81)	0.271
0 to 6 mo	-3.92 (5.09)	-3.00 (5.79)	0.362

Participants in both arms were collapsed into a single group and dichotomized based on if they were enrolled before or after March 2020

[a] n=77

[b] n=82

[c]*p*-values for between-group differences were calculated using t-tests

© 2022 Popp CJ et al. *JAMA Network Open*.

eTable 3. Baseline Dietary Intake Between the Standardized and Personalized Groups

Dietary measures	Overall	Standardized[a]	Personalized[b]	
	Mean (SD)	Mean (SD)	Mean (SD)	*p*-value[c]
Energy, kilocalories	1826 (778)	1690 (669)	1960 (856)	0.051
Carbohydrate, grams	201 (98.4)	189 (84.6)	213 (110.0)	0.16
Carbohydrate, % energy	44.3 (11.0)	44.8 (11.1)	43.8 (10.9)	0.61
Total sugars, grams	83.9 (53.0)	76.6 (38.7)	91.0 (63.5)	0.12
Total sugars, % energy	18.4 (8.1)	18.3 (6.55)	18.4 (9.44)	0.95
Fiber, grams	17.5 (10.6)	16.2 (9.20)	18.8 (11.8)	0.17
Fiber, grams/1000 kilocalories	10.1 (5.8)	10.0 (4.9)	10.1 (6.6)	0.95
Total fat, grams	74.3 (39.1)	66.6 (34.1)	82.0 (42.3)	0.02
Total fat, % energy	36.0 (9.2)	35.0 (9.46)	37.1 (8.95)	0.20
Saturated fat, grams	23.6 (15.0)	21.4 (13.9)	25.7 (15.9)	0.11
Saturated fat, % energy	11.3 (3.9)	11.1 (3.87)	11.5 (4.03)	0.49
Monounsaturated fat, grams	26.4 (14.7)	23.3 (11.7)	29.5 (16.7)	0.02
Monounsaturated fat, % energy	12.8 (4.3)	12.3 (4.00)	13.2 (4.57)	0.25
Polyunsaturated fat, grams	18.0 (11.0)	16.3 (9.8)	19.8 (12.0)	0.07
Polyunsaturated fat, % energy	8.9 (4.2)	8.7 (4.08)	9.1 (4.35)	0.59
Protein, grams	87.3 (40.6)	82.9 (36.9)	91.5 (43.8)	0.23
Protein, % energy	19.7 (6.8)	20.2 (6.53)	19.1 (7.09)	0.35

[a]Standardized n=64
[b]Personalized n=65
[c]*p*-values for between-group differences were calculated using t-tests

© 2022 Popp CJ et al. *JAMA Network Open*.

eTable 4. Changes in Dietary Measures From Baseline (Month 0) to 3 and 6 Months

**Dietary measures**	**Standardized**[a]	**Personalized**[b]	
	Mean (SD)	Mean (SD)	*p*-value[c]
**Energy, kilocalories**			
0 to 3 months	94 (742)	-341 (938)	0.02
0 to 6 months	-86 (741)	-371 (872)	0.09
**Carbohydrate, grams**			
0 to 3 months	5.8 (90.9)	-47.0 (113)	0.02
0 to 6 months	-18.3 (99.7)	-51.6 (105)	0.12
**Carbohydrate, % energy**			
0 to 3 months	0.7 (18.2)	-4.0 (13.4)	0.19
0 to 6 months	-1.3 (13.7)	-2.6 (12.2)	0.64
**Total sugars, grams**			
0 to 3 months	11.8 (63.4)	-19.6 (72.2)	0.04
0 to 6 months	-5.7 (46.4)	-22.6 (58.3)	0.12
**Total sugars, % energy**			
0 to 3 months	2.7 (11.8)	-1.9 (9.4)	0.06
0 to 6 months	-0.1 (8.2)	-1.3 (8.3)	0.51
**Fiber, grams**			
0 to 3 months	0.5 (8.50)	-0.2 (11.7)	0.75
0 to 6 months	0.6 (10.9)	-3.1 (14.3)	0.16
**Fiber, grams/1000 kilocalories**			
0 to 3 months	0.4 (6.4)	1.4 (6.9)	0.50
0 to 6 months	1.0 (5.5)	0.1 (8.4)	0.55
**Total fat, grams**			
0 to 3 months	5.9 (43.1)	-9.5 (42.9)	0.10
0 to 6 months	-1.3 (37.8)	-13.7 (50.2)	0.18
**Total fat, % energy**			
0 to 3 months	0.3 (11.3)	3.2 (10.2)	0.23
0 to 6 months	1.0 (12.6)	0.8 (14.2)	0.93
**Saturated fat, grams**			
0 to 3 months	-0.4 (13.9)	-5.5 (17.0)	0.13
0 to 6 months	-1.0 (16.9)	-4.44 (19.3)	0.36
**Saturated fat, % energy**			
0 to 3 months	-0.9 (4.5)	0.03 (4.6)	0.38
0 to 6 months	0.3 (5.9)	-0.2 (5.2)	0.64
**Monounsaturated fat, grams**			
0 to 3 months	3.0 (14.5)	-1.4 (18.1)	0.22
0 to 6 months	-0.3 (13.7)	-4.6 (20.3)	0.24
**Monounsaturated fat, % energy**			
0 to 3 months	0.8 (5.1)	2.1 (5.1)	0.24
0 to 6 months	0.3 (5.4)	0.8 (7.1)	0.69
**Polyunsaturated fat, grams**			
0 to 3 months	2.7 (17.0)	-1.3 (11.8)	0.22
0 to 6 months	0.04 (11.8)	-3.3 (12.8)	0.20
**Polyunsaturated fat, % energy**			
0 to 3 months	0.3 (5.3)	1.1 (5.9)	0.54
0 to 6 months	0.2 (5.3)	0.2 (5.5)	0.99
**Protein, grams**			
0 to 3 months	-1.0 (48.1)	-11.3 (56.7)	0.36
0 to 6 months	-4.4 (44.0)	-14.3 (41.0)	0.27
**Protein, % energy**			

© 2022 Popp CJ et al. *JAMA Network Open*.

0 to 3 months	-2.1 (10.9)	1.7 (7.5)	0.08
0 to 6 months	-0.5 (7.8)	1.2 (7.0)	0.25

[a]Standardized: n=36 at 3 months; n=45 at 6 months
[b]Personalized: n=50 at 3 months; n=48 at 6 months
[c]*p*-values for between-group differences were calculated using t-tests

© 2022 Popp CJ et al. *JAMA Network Open*.

eTable 5. End-of-Study Questionnaire

	Combined		Standardized		Personalized		P-value
**Variable**	**mean (sd)**	**n**	**mean (sd)**	**n**	**mean (sd)**	**n**	
1. I changed the meals I ate based on the feedback that the app gave me.	3.5 (1.4)	135	3.4 (1.4)	65	3.7 (1.3)	70	0.158
2. I changed the types of foods I ate based on the feedback that the app gave me	3.5 (1.3)	135	3.3 (1.5)	65	3.7 (1.2)	70	0.192
3. The use of the app kept me engaged and motivated to participate in the program	3.2 (1.6)	135	3.0 (1.6)	65	3.3 (1.6)	70	0.284
4. I will continue using the app after the program is over.	2.3 (1.6)	134	2.2 (1.6)	65	2.5 (1.7)	69	0.255
5. I used a different app to track my food during the program.	2.4 (1.7)	121	2.5 (1.8)	60	2.3 (1.6)	61	0.650
6. The topic of the Webex sessions were applicable to me personally.	4.4 (0.9)	135	4.4 (0.8)	65	4.4 (0.9)	70	0.422
7. I did not know a lot of the information that the Webex sessions contained.	3.2 (1.3)	135	3.2 (1.2)	65	3.1 (1.3)	70	0.377
8. I felt connected to my group members in the Webex sessions.	3.9 (1.3)	134	3.7 (1.3)	65	4.1 (1.2)	69	0.050
9. The Webex sessions motivated me to make lifestyle changes.	4.1 (1.0)	135	4.3 (0.8)	65	3.9 (1.2)	70	0.147
10. I found Webex easy to use.	4.4 (1.0)	135	4.3 (1.1)	65	4.5 (0.9)	70	0.355
11. The group to which I was randomly assigned (low-fat or personalized) negatively affected my motivation	1.6 (1.0)	135	1.8 (1.1)	65	1.5 (0.9)	70	0.055

End of study questionnaire was administered at 12-month visit and included a Likert scale from 1 to 5 (1,2 = disagree; 3 = neutral; 4,5 = agree). Data reported are means (standard deviation). T-tests were run between the Standardized and Personalized arms; $p<0.05$

© 2022 Popp CJ et al. *JAMA Network Open*.

eFigure 1. Schematic of Study Design

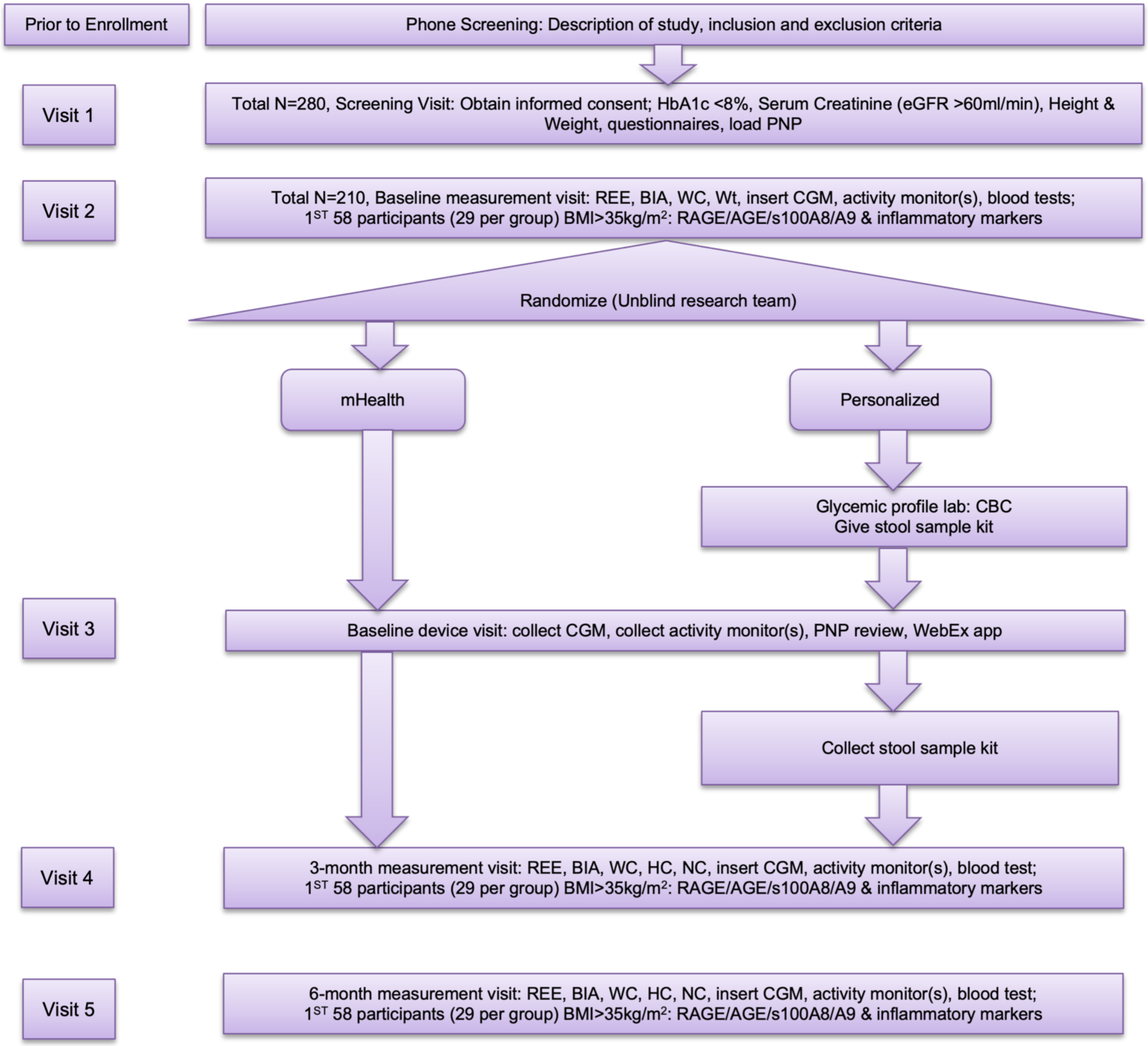

In the original application, the Standardized group was labeled as *mHealth*.

© 2022 Popp CJ et al. *JAMA Network Open*.

eFigure 2. Weight Loss Variability Between the Standardized (Top) and Personalized (Bottom) Groups

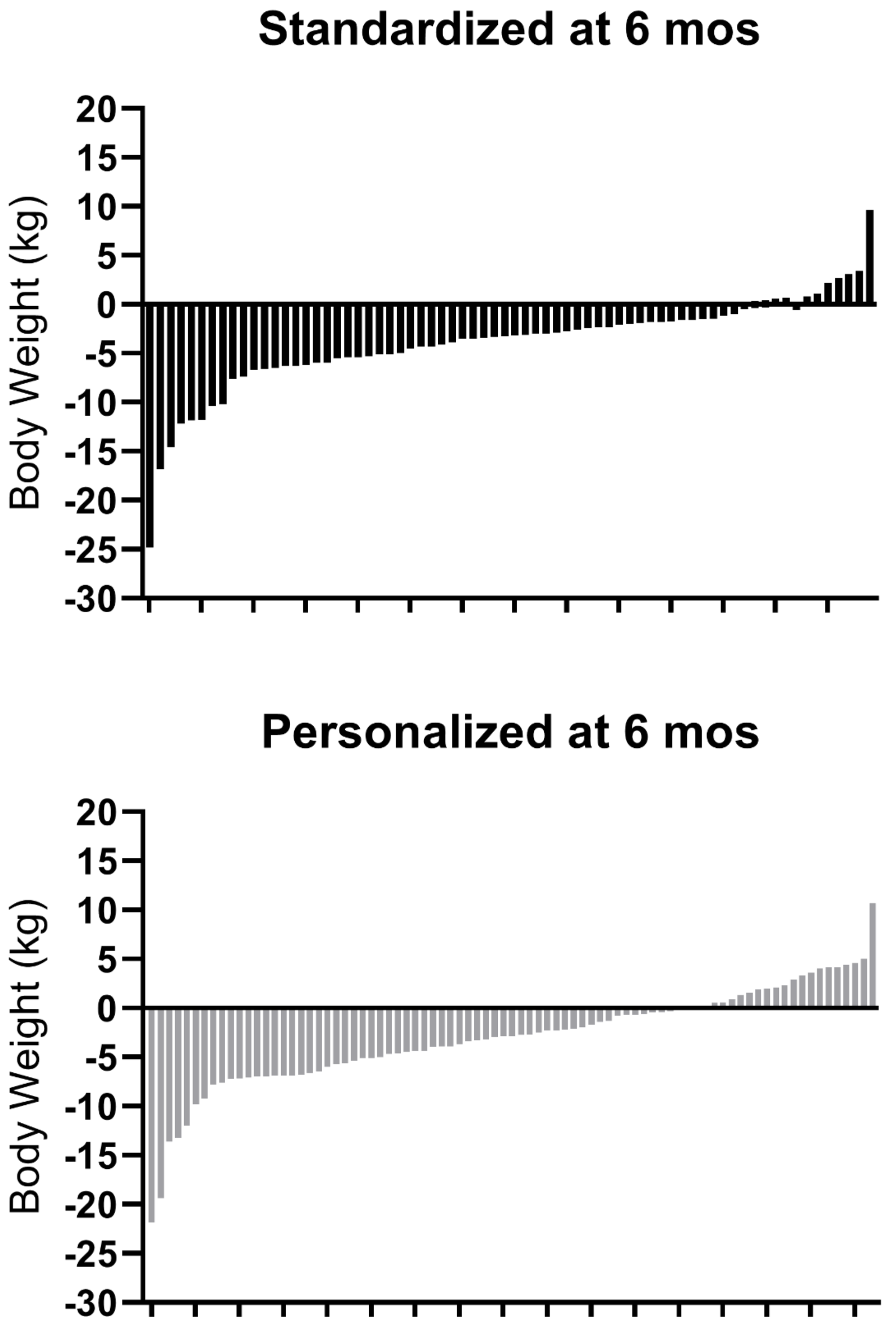

Data included are participants with baseline and 6 mos body weights.

© 2022 Popp CJ et al. *JAMA Network Open*.

eReferences

1. Popp CJ, St-Jules DE, Hu L, et al. The rationale and design of the personal diet study, a randomized clinical trial evaluating a personalized approach to weight loss in individuals with pre-diabetes and early-stage type 2 diabetes. *Contemporary Clinical Trials*. 2019;79. doi:10.1016/j.cct.2019.03.001
2. Mifflin MD, St Jeor ST, Hill LA, Scott BJ, Daugherty SA, Koh YO. A new predictive equation for resting energy expenditure in healthy individuals. *Am J Clin Nutr*. 1990;51(2):241-247. doi:10.1093/ajcn/51.2.241
3. Thomas DM, Gonzalez MC, Pereira AZ, Redman LM, Heymsfield SB. Time to correctly predict the amount of weight loss with dieting. *J Acad Nutr Diet*. 2014;114(6):857-861. doi:10.1016/J.JAND.2014.02.003
4. Nunes CL, Jesus F, Francisco R, et al. Adaptive thermogenesis after moderate weight loss: magnitude and methodological issues. *Eur J Nutr*. Published online 2021. doi:10.1007/S00394-021-02742-6

© 2022 Popp CJ et al. *JAMA Network Open*.
