# Supplementary material for: Effect of a Personalized Diet to Reduce Postprandial Glycemic Response vs a Low-fat Diet on Weight Loss in Adults With Abnormal Glucose Metabolism and Obesity: A Randomized Clinical Trial
Source: JAMA Netw Open. 2022 Sep 28;5(9):e2233760. doi: 10.1001/jamanetworkopen.2022.33760 (PMC9520362; doi:10.1001/jamanetworkopen.2022.33760)
Supplement: Supplement 3. — Data Sharing Statement [file jamanetwopen-e2233760-s003.pdf]

Data Sharing Statement

Popp. Effect of a Personalized Diet to Reduce Postprandial Glycemic Response vs a Low-fat Diet on Weight Loss in Adults With Abnormal Glucose Metabolism and Obesity. *JAMA Netw Open*. Published September 28, 2022. doi:10.1001/jamanetworkopen.2022.33760

Data
Data available: Yes
Data types: Deidentified participant data, Data dictionary
How to access data: Data will be made available upon request to the Principal Investigator (Mary.Sevick@nyulangone.org)
When available: With publication

Supporting Documents
Document types: None

Additional Information
Who can access the data: Anyone requesting the data
Types of analyses: For any purpose
Mechanisms of data availability: After approval of a proposal by the Principal Investigator
